# An ensemble learning approach to reverse-engineering transcriptional regulatory networks from time-series gene expression data

**DOI:** 10.1186/1471-2164-10-S1-S8

**Published:** 2009-07-07

**Authors:** Jianhua Ruan, Youping Deng, Edward J Perkins, Weixiong Zhang

**Affiliations:** 1 Department of Computer Science, The University of Texas at San Antonio, San Antonio, TX 78249, USA; 2 SpecPro Inc., Vicksburg, MS 39180, USA; 3 Department of Biological Sciences, University of Southern Mississippi, Hattiesburg, MS 39406, USA; 4 Environmental Laboratory, U.S. Army Engineer Research and Development Center, Vicksburg, MS 39180, USA; 5 Department of Computer Science and Engineering, Washington University in St Louis, St Louis, MO 63130, USA; 6 Department of Genetics, Washington University School of Medicine, St Louis, MO 63110, USA

## Abstract

**Background:**

One of the most challenging tasks in the post-genomic era is to reconstruct the transcriptional regulatory networks. The goal is to reveal, for each gene that responds to a certain biological event, which transcription factors affect its expression, and how a set of transcription factors coordinate to accomplish temporal and spatial specific regulations.

**Results:**

Here we propose a supervised machine learning approach to address these questions. We focus our study on the gene transcriptional regulation of the cell cycle in the budding yeast, thanks to the large amount of data available and relatively well-understood biology, although the main ideas of our method can be applied to other data as well. Our method starts with building an ensemble of decision trees for each microarray data to capture the association between the expression levels of yeast genes and the binding of transcription factors to gene promoter regions, as determined by chromatin immunoprecipitation microarray (ChIP-chip) experiment. Cross-validation experiments show that the method is more accurate and reliable than the naive decision tree algorithm and several other ensemble learning methods. From the decision tree ensembles, we extract logical rules that explain how a set of transcription factors act in concert to regulate the expression of their targets. We further compute a profile for each rule to show its regulation strengths at different time points. We also propose a spline interpolation method to integrate the rule profiles learned from several time series expression data sets that measure the same biological process. We then combine these rule profiles to build a transcriptional regulatory network for the yeast cell cycle. Compared to the results in the literature, our method correctly identifies all major known yeast cell cycle transcription factors, and assigns them into appropriate cell cycle phases. Our method also identifies many interesting synergetic relationships among these transcription factors, most of which are well known, while many of the rest can also be supported by other evidences.

**Conclusion:**

The high accuracy of our method indicates that our method is valid and robust. As more gene expression and transcription factor binding data become available, we believe that our method is useful for reconstructing large-scale transcriptional regulatory networks in other species as well.

## Background

A major challenge in computational biology is to reveal the *cis*-regulatory logics of gene expression through analysis of high-throughput genomic data, for example, genomic sequences and gene expression data. A common practice is to first identify putatively co-regulated genes by clustering gene expression patterns [1-3], and then search for common motifs from the promoter sequences of the genes in the same cluster [4-7]. Such enriched motifs, if identified, are often believed to be the binding motifs of a common transcription factor (TF). This strategy has been successful on small datasets, but is limited by its strong assumptions that co-expression means co-regulation and vice versa [8,9]. Furthermore, in higher eukaryotes, genes are typically regulated by combinations of TFs, and TF binding motifs are often organized into modular units [10]. Although some progress has been made [11-14], it is still difficult to precisely identify combinatorial motifs. Finally, these methods by themselves do not reveal the actual TFs that bind to the sequence motifs.

Recently, several studies have attempted to build quantitative or qualitative models to predict gene expression levels from the regulatory motifs on their promoter sequences. Bussemaker et al. [15] and others [9,16] proposed to model gene expression levels as a linear regression of binding motif scores, and applied feature selection techniques to find the most significant motifs. These methods have been shown effective for discovering conserved short motifs related to several biological processes in *S. cerevisiae *[9,15,16]. However, they are limited by the assumption of a linear additivity of binding motifs, and therefore is unable to represent complex *cis*-regulatory logics such as AND and OR relations [17,18]. Furthermore, such linear models are often difficult to interpret.

In order to obtain more realistic models with better interpretability, several classification models have been proposed. Decision tree methods have been successfully applied to find motif combinations that best separate two classes of genes [19,20]. Other tree-based methods such as multivariate regression trees and bi-dimensional regression trees have been developed to model the transcriptional regulation of gene expressions over several time points simultaneously [21,22]. Simonis et al. [23] combined a string-based motif finding method and linear discriminant analysis to identify motif combinations that can separate true regulons from false ones. Middendorf et al. [24] used an ensemble of decision trees to model gene expression levels by combining putative binding motifs and the expression levels of putative TFs. Segal et al. [13] and Beer and Tavazoie [18] built Bayesian networks to explain gene expression patterns from motifs. In these models, the predictors (features) are the matching scores of promoter sequences to putative binding motifs, and the predictions (responses) can be continuous or discrete gene expression levels or categorical cluster labels. A common goal of these methods is to derive simple and intuitive rules from the classification models, for example, in the form of "if a gene has motif A and motif B, it will be up-regulated under condition c".

The features used in the above models are usually de novo sequence motifs that are identified from the promoters of the genes under study [13,16,18,19,23], or existing motifs that are obtained independently [21,24]. One can also enumerate all possible words up to a certain length [9,15]. The problem with these types of features is that they generally have low quality, are incomplete, and may contain many variations for the same motif. To address this issue, chromatin immunoprecipitation microarray (ChIP-chip) data [25], which represent the relative binding strengths of TFs to the promoter regions of their target genes, have been used as a substitute of motif scores. For example, Banerjee and Zhang [26] directly applied the method of Pilpel et al. [12] to ChIP-chip data to identify TF combinations; Gao et al. [27] replaced the variables in the linear model of Bussemaker et al. [15] with ChIP-chip data and identified significant regulators for many experimental conditions. We have recently compared several types of features using decision tree models and showed that using ChIP-chip data as features generally result in better classification accuracy that using other types of features, when gene expression and ChIP-chip data are obtained from similar conditions (e.g., normal growth conditions) [20]. An additional advantage of using ChIP-chip data than binding motifs is that the former directly creates a link between a TF and its target genes. While discovering binding motifs of TFs is still important, it can be separated from the learning of transcriptional regulatory networks.

In this paper, we propose a novel approach that combines the strengths of several recent methods in order to learn a highly accurate and reliable transcriptional regulation model, and combine the models learned from different time points and/or different experiments to construct a dynamic transcriptional regulatory network. We use TF binding data rather than binding motifs as predictor variables. We use decision trees as our underlying model, because decision trees are efficient to learn, easy to understand, can capture complex regulatory logics, and have feature selection built in [28-32]. In order to improve the accuracy and robustness of our model, we use an ensemble learning approach to learn multiple decision trees for each training set. From these learned decision trees, we then extract rules in the form of, for example, "if a gene can be bound by TF A and TF B, it will be up-regulated under condition c at time point t". Furthermore, we propose a profile approach to reveal the condition-specific or time-dependent effects of TFs. We also propose a spline interpolation method to combine results from multiple time series data. Such an integrated approach can substantially eliminate noises in individual data sources and improve modeling accuracy. To validate our approach, we apply it to three sets of yeast cell cycle gene expression data [33,34] and whole-genome yeast TF binding data [25]. It is known that nine TFs – Mbp1, Swi4, Swi6, Mcm1, Fkh1, Fkh2, Ndd1, Swi5 and Ace2 – regulate a large number of yeast cell cycle dependent genes [35,36]. Specifically, MBF (a complex of Mbp1 and Swi6) and SBF (a complex of Swi4 and Swi6) control late G1 genes; Mcm1, together with Fkh1 or Fkh2, recruits Ndd1 in late G2 and controls the transcription of G2/M genes; and Swi5 and Ace2 regulate genes at the end of M and early G1. This model was developed using a small set of genes and was recently confirmed by a number of computational studies [25,37]. We thus applied our method to the cell cycle data to verify the accuracy of our method. In addition, by performing a large-scale analysis, we expect to construct a more detailed transcriptional regulatory network as well as capturing new, testable hypotheses for yeast cell cycle regulations.

We demonstrate that our method is able to identify biologically significant time-dependent regulatory rules, and the learned regulatory rules can be used as the basic building elements of a dynamic transcriptional regulatory network. Statistical evaluation indicates that the rules are robust and reliable. The transcriptional regulatory network constructed by our method for the yeast cell cycle genes agrees very well with the existing knowledge. Many transcriptional regulatory rules for yeast cell cycle genes discovered by our approach have been confirmed by the literature, while the other rules may yield additional insights into the biological process.

## Results

### Overview of the approach

**Figure 1:**
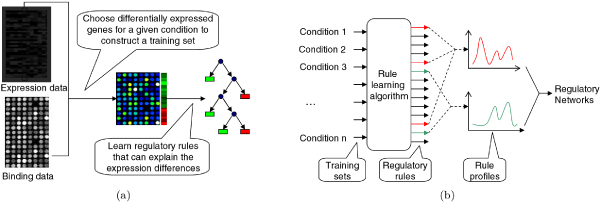
**Overview of our approach**. (a) Constructing the training set and learning a set of regulatory rules for each condition. (b) Generating rule profiles and combining them into transcriptional regulatory networks.

Our method takes as input the expression data and TF binding data of a set of genes and proceeds in two stages (Figure 1). In the first stage, we construct a training data set for each experimental condition of the expression data, and obtain a set of regulatory rules using a decision tree ensemble learning approach. In the second stage, we generate profiles for the regulatory rules, integrate results from multiple data sets, and combine rules into a transcriptional regulatory network.

A training set contains a set of instances (genes), each of which is represented by a vector. The vector corresponding to the *j*th gene is defined as <*B*_1*j*_, *B*_2*j*_,..., *B*_*nj*_, *C*_*kj*_>, where *B*_*ij *_is the strength of the *i*th TF binding to the promoter of the *j*th gene, and *C*_*kj *_specifies the expression state of the *j*th gene under condition *k*. For simplicity, we consider only binary states: "up-regulated" and "not-up-regulated", while it can be easily generalized to any number of states. In this paper, we refer to up-regulated and not-up-regulated genes as positive and negative genes, respectively (see Materials and Methods). The strength of a TF binding to a promoter sequence is represented by the negative logarithm of the binding p-value.

Once we have constructed the training set, we then learn a set of decision trees to classify gene expression states based on TF binding data. A decision tree is a rooted tree consisting of internal nodes and leaf nodes. Each internal node corresponds to a test of the binding of a selected TF to a gene, for example, "can TF A bind to gene g?". Each leaf is a prediction of the state of that gene, for example, "gene g is up-regulated". An internal node has two branches: the right branch is chosen when the test succeeds; and the left branch is chosen when it fails. Therefore, a path from the root to a leaf defines a possible regulatory rule, for example, " if a gene can be bound by TF A and TF B, it will be up-regulated at time t".

As decision tree learning algorithms are typically greedy, they are not guaranteed to find the optimal tree [28,29]. Furthermore, in many cases, since some features may be highly correlated with some other features and are considered redundant, they may not be selected by the tree even if they are as good as the other features. Therefore, a single decision tree may not identify all possible regulatory logics. To address these issues, we use an ensemble learning approach to construct multiple decision trees. This will not only improve the modeling accuracies, but also provides many alternative models, which can then be compared and combined to give a more complete and accurate set of rules.

From decision trees, we extract regulatory rules, and calculate a significance score (p-value) for each rule (see Materials and Methods). Only significant rules (p-values < 0.001) are retained. For a rule that appears in multiple decision trees corresponding to the same training data, the most significant p-value is taken. Furthermore, a regulatory rule may very often be discovered at multiple time points. The negative logarithm of the p-value of a rule at a given time point reflects the regulation strength of the rule at that time. Thus it is informative to plot the negative logarithm of the p-value of a rule as a function of time; such a plot is referred to as a rule profile. Finally, when two or more microarray time series are available for the same biological process, we combine the rule profiles learned from different time series. As different experiments may have different sampling rates, we approximate each rule profile with a spline interpolation, and combine the profiles for the same rule from different time series to construct a single integrated profile. In the last step, we identify for each rule the most probable experimental conditions under which it functions and the set of genes that it regulates, and organize this information into a transcriptional regulatory network.

### Regulatory rules learned by the simple decision tree approach

Gene expression during the yeast cell cycles has been measured with several different synchronization methods. We applied our method to three data sets obtained from the methods of CDC28, CDC15 and *α*-factor [33,34]. To illustrate the learning of decision trees and regulatory rules, our discussion will first focus on the rules learned from the CDC28 data set. In this subsection, we only learned a single decision tree for each time point of the CDC28 data set. We will later present results on decision tree ensembles, and combine the results obtained from all three data sets.

**Figure 2:**
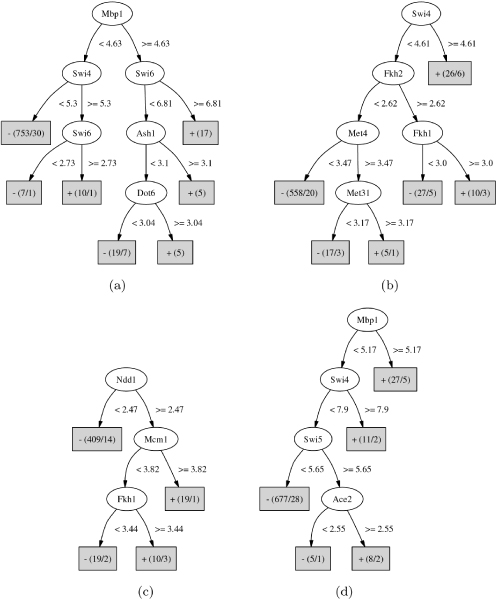
**Decision trees learned from the CDC28 cell cycle data set**. (a) 20 minutes. (b) 40 minutes. (c) 70 minutes. (d) 100 minutes. Each oval represents an internal node and each box represents a leaf node. The text inside an internal nodes is a regulator, while the text associated with an edge is a test on a DNA binding p-value. The text inside a leaf node is a prediction of the state of a gene. The numbers of positive and negative genes are included in parentheses. For example, "+(19/1)" in the 40-minute tree means that the rule will predict positive and there are 19 genes satisfying this rule, of which 18 are true positive and the remaining one is false positive.

Figure 2 shows the decision trees learned from the 20-, 40-, 70- and 100-minute time points data, corresponding to late G1, S, G2/M and early G1 phases, respectively. The method rediscovered all nine known TFs in appropriate cell cycle phases. As can be seen, Swi4, Swi6 and Mbp1 appeared in 20 and 100 minute. Ndd1, Mcm1, Fkh1 and Fkh2 appeared in 40 and 70 minute. Swi5 and Ace2 appeared in 100 minute.

We then extracted regulatory rules from the trees by a depth-first search from the root node to all leaf nodes labeled as positive. A node was included in a rule only if its right branch was taken by the path. For example, we extracted the following two rules from the 70-minute tree (Figure 2(c)): (Ndd1 ≥ 2.47) ∩ (Mcm1 ≥ 3.82), and (Ndd1 ≥ 2.47) ∩ (Fkh1 ≥ 3.44), where n represents logical AND. According to the first rule, genes that can be bound by Ndd1 with a p-value less than *e*^-2.47 ^and by Mcm1 with a p-value less than *e*^-3.82 ^are up-regulated at the 70-minute time point. For simplicity, we omit the p-value thresholds of binding data in later discussions, and simplify the two rules as Ndd1 ∩ Mcm1 and Ndd1 ∩ Fkh1, respectively. It is worth noting, however, that the thresholds are learned automatically and may be different in different rules.

Each rule has some number of supporting genes in the training set, from which a p-value can be calculated. For example, the rule Ndd1 ∩ Mcm1 in the 70-minute tree is supported by 18 positive and 1 negative genes out of a total of 41 positive and 416 negative genes. This corresponds to a p-value ≈ 10^-20^. (For the detail of calculating the p-value of a rule, see Materials and Methods.)

The most significant rule identified from the 20-minute time point is Mbp1 ∩ Swi6 (*p *= 10^-19^). The other three significant rules are Swi4 ∩ Swi6 (*p *= 10^-9^), Mbp1 ∩ Dot6 (*p *= 10^-5^) and Mbp1 ∩ Ash1 (*p *= 10^-5^) (Figure 2(a)). Ash1 is known to accumulate in the daughter cell throughout the G1 phase, inhibiting transcription of the HO endonuclease, thereby preventing mating-type switching [38]. Dot6 has been shown to affect pseudohyphal differentiation [39]. Genes up-regulated at 40 minute are described by three significant rules: Swi4 (*p *= 10^-17^), Fkh1 ∩ Fkh2 (*p *= 10^-6^), and Met4 ∩ Met31 (*p *= 10^-4^) (Figure 2(b)). Met4 and Met31 cooperate to regulate the sulfur amino acid pathway [40]. A cluster of genes involved in the biosynthesis of methionine has previously been reported as being regulated by the yeast cell cycle [33]. The two significant rules for 70-minute time point (Figure 2(c)) are both well-known: Ndd1 ∩ Mcm1 (*p *= 10^-20^) and Ndd1 ∩ Fkh1 (*p *= 10^-6^). Rules identified for 100-minute time point include early G1 phase TFs, Swi5 ∩ Ace2 (*p *= 10^-5^), as well as late G1 phase TFs Mbp1 (*p *= 10^-20^) and Swi4 (*p *= 10^-8^).

### Decision trees and regulatory rules learned by an ensemble approach

The above examples illustrated the ability of the single decision tree approach in identifying the known TFs and associating them with appropriate cell cycle phases. However, as there might be other decision trees that explain the data as well, we may have missed some interesting TFs or TF combinations. In this subsection, we show how an ensemble learning approach can be used to extract alternative regulatory rules, thus providing a more complete image of the transcriptional regulation for the yeast cell cycle.

Many machine learning approaches have been developed for learning tree ensembles (for review, see [41]), including Bagging [42] and Boosting [43]. One basic idea in these methods is to repeatedly perturb the original data (e.g., change weights of instances, or sample instances with replacement), and learn a decision tree from each perturbed data set. Each decision tree stands for an alternative model. To make a prediction, an instance is passed to individual decision trees and their predictions are combined by voting [41]. We adopt the basic idea, while also considering a unique feature of our data set: the number of negative instances is much larger than the number of positive ones. Such a skewed class distribution deteriorates the learning ability of most machine learning algorithms [44], including decision trees. To overcome this difficulty, we split negative instances into smaller subsets and combine each subset of negative instances with all positive instances to form a training set, from which a decision tree is learned (see Materials and Methods). We refer to this method as Splitting. By this approach, we effectively adjust the class distribution to a preferred value without losing any information in the original data set. The prominent regulatory rules will likely be present in many trees and stand out when the trees are combined. A similar approach has been successfully applied to learn decision trees for detecting credit-card frauds [45]. Table 1 shows a selected list of significant rules discovered by the Splitting approach when applying to the 20-, 40-, 70- and 100-minute CDC28 data set. A complete list is included in Additional File 1. As can be seen, the Splitting approach discovered not only all the rules identified by the simple decision tree approach, but also several additional synergetic relationships among the known cell cycle TFs, such as Mbp1 ∩ Swi4 and Fkh2 ∩ Ndd1. Furthermore, several rules involving additional cell-cycle related TFs were discovered. For example, Stb1 and Ecm22 were found in 20 minute, Cbf1, Hsf1, Rgm1 and Mth1 in the 40-minute data, Nrg1 and Smp1 in 70-minute, Ste12, Hir2 and Mss11 in 100-minute data. Among them, Stb1 is known to regulate in G1 [46]; Cbf1 binds to centromere and is involved in DNA replication and methionine biosynthesis together with Met4 [40,47]; Nrg1 and Smp1 were recently found to regulate filamentous growth [48].

**Table 1:** Regulatory rules learned by the Splitting approach

20 min		40 min		70 min		100 min	
Rule	*p*-value	Rule	*p*-value	Rule	*p*-value	Rule	*p*-value

Mbp1	10^-31^	Swi4	10^-17^	Mcm1 ∩ Ndd1	10^-25^	Mbp1	10^-20^
Mbp1 ∩ Swi6	10^-26^	Mth1 ∩ Swi4	10^-11^	Fkh2	10^-21^	Swi4	10^-18^
Stb1 ∩ Swi4	10^-15^	Fkh2	10^-10^	Ndd1	10^-17^	Swi4 ∩ Swi6	10^-14^
Swi4 ∩ Swi6	10^-9^	Fkh1 ∩ Fkh2	10^-6^	Fkh2 ∩ Ndd1	10^-15^	Ste12 ∩ Swi4	10^-8^
Swi4	10^-7^	Met4	10^-5^	Fkh1 ∩ Ndd1	10^-6^	Hir2 ∩ Swi4	10^-5^
Mbp1 ∩ Swi4	10^-6^	Fkh2 ∩ Msn1	10^-5^	Fkh1 ∩ Fkh2	10^-4^	Mbp1 ∩ Mss11	10^-5^
Dot6 ∩ Mbp1	10^-5^	Hsf1	10^-5^	Mcm1	10^-4^	Ace2 ∩ Swi5	10^-5^
Ash1 ∩ Mbp1	10^-5^	Met4 ∩ Met31	10^-5^	Nrg1 ∩ Smp1	10^-3^	Mbp1 ∩ Stb1	10^-5^
Ecm22 ∩ Mbp1	10^-3^	Met4 ∩ Cbf1	10^-5^			Swi5	10^-4^

We repeated the learning method on the CDC15 and *α*-factor data sets, and the resulting regulatory rules are listed in Additional Files 2 and 3, respectively. Not unexpected, most of the significant rules involve at least one of the nine well-known TFs. Two significant rules identified in the *α*-factor data set involve novel transcription factors: Yap5 (*p *= 10^-10 ^at the 14-minute time point and 10^-8 ^at the 77-minute time point) and Gat3 (*p *= 10^-9 ^at the 14-minute time point and 10^-8 ^at the 77-minute time point). The roles of these two TFs in G1 are still unknown and may deserve further investigation. Later, we will introduce a method for combining the rules learned from the three data sets.

### Estimating the model accuracy

A critical issue of classification algorithms is generalization, i.e., how well a learned model can be applied to new data that has not been seen by the learning algorithm? When the number of features is large, a classifier is often over-fitted, in that it performs very well on training data, while performs poorly on unseen data. Therefore, it is important to evaluate the accuracy of a classifier on unseen data, which is typically done by a cross-validation procedure (see Materials and Methods). In this work we used 10-fold cross-validation.

A straightforward measurement of classification accuracy is the percentage of correctly classified instances (denoted as *A*). However, this tends to underestimate the true error, especially when the ratio of positive and negative instances is skewed. For example, if there are 990 negative and 10 positive instances, simply predicting everything as negative will achieve 99% accuracy. Therefore, we compute the kappa statistic *K *to measure accuracy. *K *is a better estimation of the true classification accuracy, and is guaranteed to be no greater than *A *(See Materials and Methods). Furthermore, it has been suggested that *K *< 0.4 indicates a poor classifier, *K *> 0.75 implies an excellent classifier, and 0.4 <*K *< 0.75 means a reasonably good classifier [49].

**Figure 3:**
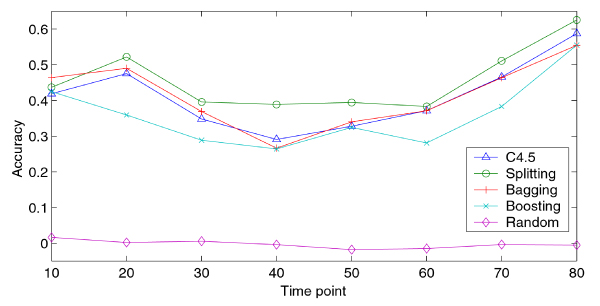
**Ten-fold cross-validation accuracy of C4.5, Bagging, Boosting and Splitting**. Experiments were done on eight different time points of CDC28 data set. Implementation of Bagging, Boosting and C4.5 were obtained from the WEKA package [52]. C4.5 was also used as the base level classifier for Bagging, Boosting and Splitting. Default parameters were used for C4.5, Bagging and Boosting. Splitting were done according to Materials and Methods.

Figure 3 shows the cross-validation kappa statistics of the single decision tree approach (C4.5) and three ensemble approaches (Bagging, Boosting and Splitting) on eight time points of the CDC28 data set (see Methods). The training and test data sets used by different methods are exactly the same. The Splitting method has the best *K *under almost all conditions, with a value at least 0.4 in essentially all time points. Furthermore, when we randomized the training set by randomly exchanging positive and negative labels, the Splitting method yield kappa statistics smaller than 0.02 in all cases (average = -0.002). This confirms that the rules learned are not random.

### Obtaining rule profiles and integrating results from multiple experiments

**Figure 4:**
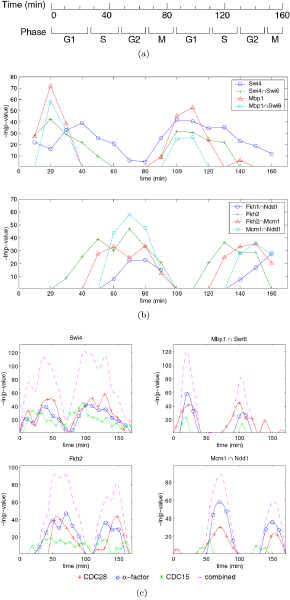
**Example rule profiles**. (a) Approximate cell cycle phases in CDC28 data set. (b) Rule profiles obtained from CDC28 data set alone. (c) Integrating rule profiles obtained from CDC28, CDC15 and *α*-factor data sets, aligned on the basis of CDC28 cell cycles.

The negative logarithm of the p-value of a rule under a given condition reflects the significance of the rule. We obtained the profile of each rule by plotting its -log *p *as a function of time. Such a plot can be used for several purposes. First, the wave form shows the change of significance score of a regulatory rule over time. Therefore it reveals the most probable period of time during which the rule regulates. Second, the pattern of rule profiles in a time series reveals certain properties of the biological process (for example, critical time point for a phase transition or length of a cell cycle). Third, comparing the profile of a rule with the expression pattern of the corresponding TFs indicates the direction of the regulation (see Discussion). Figure 4(b) illustrates rule profiles of G1 and G2/M TFs Mbp1, Swi4, Swi6, Ndd1, Mcm1, Fkh1 and Fkh2 obtained from the CDC28 data set. These profiles all showed clear periodicity. Their peaks agree very well to cell cycle phases determined by phenotypes and gene expression data [34] (Figure 4(a)): Swi4, Swi6 and Mbp1 peak in G1, and Ndd1, Mcm1, Fkh1 and Fkh2 peak in G2/M. The rule profiles also show that there is a significant lag between the peaks of Mbp1 and Swi4, which was also discovered by previous studies [15,25,33]. We also found a lag between the peaks of Fkh2 and Mcm1, which is different from an antagonistic (out-of-phase) relationship suggested by Bussemaker *et al. *[15], but similar to the results reported by Lee *et al*. [25]. Our results also show a significant lag between Fkh2 and Fkh1, similar to what was reported previously [25].

Since all three data sets, CDC15, CDC28, and *α*-factor, measured gene expression levels during yeast cell cycle, the gene expression patterns in them should be similar; so should the inferred profiles of regulatory rules. Therefore, it should be possible to combine the rule profiles learned from them. However, the length of a cell cycle and the sampling rates are different in these three data sets, which makes a direct point-to-point addition invalid. Previous studies have shown that it is possible to convert the time scales of the CDC15 and *α*-factor data sets to the time scale in CDC28 [50]. They found that, after conversion, expression curves in the three data sets can be aligned together very well. We used the same conversion and took the parameters from their results. As we expected, the rule profiles from different data sets can often be aligned together accurately (Figure 4(c)). We then used spline interpolation in MATLAB (the MathWorks Inc.) to convert rule profiles to continuous curves, which were then added together to obtain a combined profile for each rule. Figure 4(c) shows the integrated profiles of several rules. As shown, the integrated profiles show prominent cell cycle dependencies (period ≈ 85 minutes). Additional File 4 contains integrated rule profiles with notable cell cycle dependencies, and Additional File 5 shows integrated rule profiles that do not show clear cell cycle dependencies.

### A model for the yeast cell cycle transcriptional regulatory network

**Figure 5:**
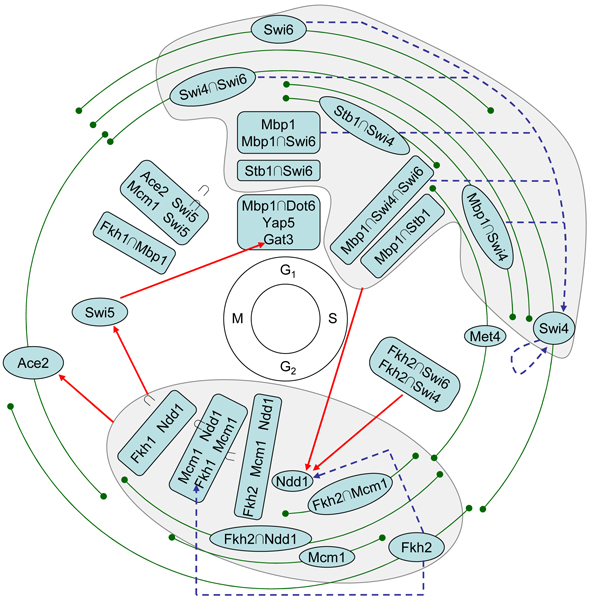
**A model for the yeast cell cycle transcriptional regulatory network learned by our method**. The text inside each dark node (ellipse or rounded rectangle) represents one or more regulatory rules. The position of a node, together with the green arc crossing it if there is one, represents the period during which the rules inside are functioning. The gray area on the top contains a module of rules that regulate late G1/S phase, and the one on the bottom encloses a module of rules that regulate G2/M phase. A solid red line represents that a set of regulatory rules regulates a TF outside the module, while a dashed blue line represents that a set of regulatory rules regulates a TF within the same module.

From the cell cycle dependent rule profiles in Additional File 4, we constructed a model of yeast cell cycle transcriptional regulatory network (Figure 5). We first determined for each rule the most probable period of time during which the rule functions, and plotted the rule in the corresponding phase of the cell cycle. We then determined the genes that each rule regulates, and created a link from the rule to a gene if the gene also appears in a regulatory rule (see Materials and Methods). We grouped most rules into two large modules (gray area), where the rules in each module share a lot of common target genes. One module is in G1/S and has Mbp1, Swi4, Swi6 and Stb1 in the rules. The other module is in G2/M and involves Fkh1, Fkh2, Ndd1 and Mcm1.

We found that the rules functioning in one phase of the cell cycle regulate TFs functioning in the next phase (solid red lines in Figure 5). This result is consistent with previous studies [25,37]. We identified two new such relations: Swi5 regulates Gat3, and Stb1 regulates Ndd1. We also found that, within each phase, rules that function earlier often regulate TFs that function later (dashed blue lines in Figure 5). For example, we found that the earliest TF in G2, Fkh2, regulates Ndd1 and Fkh1. As to our knowledge, this result has not been reported previously.

In addition, two rules combining G1 and G2 TFs (Fkh2 ∩ Swi4 and Fkh2 ∩ Swi6) function in S phase and regulate Ndd1. Another such combination, Fkh1 ∩ Mbp1, functions in M phase. We also identified several novel TFs for yeast cell cycle: Dot6, Yap5 and Gat3 in G1, and Met4 in S. Yap5 and Gat3 may be suspicious since the rules were only learned in the *α*-factor data sets, although their profiles show very clear cell cycle dependencies. Gat3 was found to be regulated by Swi5 in our network.

## Discussion

Reconstructing gene regulatory networks from gene expression data is a promising but challenging task for the post-genomic era. Traditional methods typically use a two-phase approach. The first phase groups genes into clusters according to their expression similarities [1-3]. The second phase searches for single or composite motifs that are enriched in the promoter regions of clustered genes [4,5,11-13,51]. These methods, however, are limited by their over-reliance on expression similarities. Furthermore, computational motif finding is a difficult task, while the mapping from binding motifs to corresponding TFs still remains an open problem. Statistical learning methods consider individual expression experiments separately, and fit linear models to describe the additive effects of motifs on the expression levels of individual genes [9,15,16]. These methods did not, however, explicitly take combinatorial effects of TFs into account. In this paper we proposed a supervised machine learning approach to discover regulatory rules that can be used for constructing transcriptional regulatory networks. We used decision trees to model the relationship between the expression level of a gene at a particular time point and the TFs that can bind to the promoter region of the gene, and extracted easy-to-interpret regulatory rules from decision trees. We applied an ensemble learning approach to explore alternative models and increase the modeling accuracy. We also proposed a spline interpolation approach for integrating the results obtained from multiple time series expression data sets.

Using the cell cycle data sets as examples, we demonstrated that our method is able to identify biologically significant regulatory rules from genome-wide TF binding data and gene expression data. The process of deriving all predictions in our method was unbiased by any computational or experimental knowledge. Without pre-clustering genes based on global similarity of expression patterns, we re-discovered all nine known TFs that are relevant to the yeast cell cycle and assigned them into appropriate cell cycle phases. Most regulatory rules in our results involve two or more TFs, suggesting synergetic relationships among them. For example, we have identified the collaboration of many well-known TF pairs, such as Mbp1-Swi6, Swi4-Swi6, Stb1-Swi6, Fkh1-Mcm1, Fkh1-Ndd1, Fkh2-Ndd1, Ace2-Swi5 and Met4-Met31, as well as the recently reported Met4-Cbf1 and Nrg1-Smp1 complexes. The test of other yet unverified rules may yield additional insights to the biological process.

Our method has some limitations. Although statistically significant rules often reflect biological significance, the opposite is not always true. As a result, our method may miss regulatory rules that regulate only a few genes. For example, our method failed to discover Skn7, a TF functioning in S phase, since the number of genes regulated by Skn7 is small in the given data sets to be considered statistically significant. However, this limitation is probably common to most large-scale analysis methods.

Another limitation of our method is that regulatory rules do not specify whether a participating TF contributes inductively or repressively. This is because concentrations of TF proteins are not taken into account. For example, if a rule states that "if gene g can be bound by TF f, then it can be up-regulated at time t", it is possible that g is up-regulated at t due to a reduced concentration of f, which actually implies a repressive role of f. This ambiguity may be resolved by comparing rule profiles with expression patterns of TFs. For example, the rule profile of Swi4 reaches its peak at 40 minute, while expression of Swi4 peaks at about the same time. This suggests that Swi4 is a transcriptional activator. However, the correlation does not always hold, since there may be a lag of time between the expression of a TF and its functioning, and many TFs may be modified post-transcriptionally. For example, the mRNA level of Mbp1 is almost constant during the cell cycle [33,34], although its rule profile peaks at 20 minute. We note that the same limitation exists for linear regression approaches [9,15,16].

It is also worth noting that there are alternative ways to label genes with expression states. Here we labeled a gene according to its expression level under a single condition relative to an initial condition. Alternatively, we may label a gene according to its expression level relative to the previous time point, or relative to its mean expression level in a time series. It may also be advisable to consider several consecutive time points simultaneously. We have tested some of these ideas (data not shown), and the conclusion is that all these labeling methods are valid to a certain extent (in terms of cross-validation accuracy), and there is no single method that is the best for all data sets. The labeling method we chose has the best cross-validation accuracy on average. The decision trees learned with different labeling methods are often different. Nevertheless, when the ensemble approach is used, the most significant regulatory rules tend to be stable regardless of which labeling method was used.

## Conclusion

We have proposed a decision tree ensemble approach for discovering transcriptional regulatory rules. By integrating multiple data sources, we are able to achieve high modeling accuracy. Statistical evaluation and literature validation indicate that the results are robust and reliable. We have also shown that the regulatory rules can be used as the basic building elements of a transcriptional regulatory network. As more gene expression data and TF binding data become available, we believe that our method will be useful for reconstructing large-scale transcriptional regulatory networks.

## Materials and methods

### Gene expression and TF binding data

We used *S. cerevisiae *cell cycle data synchronized with CDC28 [34], CDC15 [33] and *α*-factor [33]. For CDC28 data set, we used a 3-fold induction as the threshold for selecting positive genes. That is, a gene is positive at time point *t *if *E*_*t*_/*E*_0 _= 3, where *E*_*t *_is its expression level at time *t *and *E*_0 _is its expression level at the starting point of the time series. To have a clear separation of positive and negative genes, we chose a gene as negative only if *E*_*t*_/*E*_0 _= 1.2. Since expression levels in CDC15 and *α*-factor were normalized by a *log*_2 _ratio, we chose positive genes so that *E*_*t *_- *E*_0 _= *log*_2_3 and negative *E*_*t *_- *E*_0 _= *log*_2_1.2. Furthermore, in all three data sets, we required the expression levels of positive genes and negative genes to be greater than and less than their average expression values, respectively. We used genome-wide binding data of 113 *S. cerevisiae *TFs from Lee *et al. *[25]. We used a less stringent threshold (*p *< 0.1) than the suggested threshold (*p *< 0.001) to reduce false negatives, and depended on the learning algorithm to automatically determine an optimal threshold for each TF.

### Learning decision trees and tree ensembles

We modified a standard algorithm C4.5 for learning decision trees [28]. The implementation of the algorithm was adapted from the WEKA machine learning package [52]. In our modification, we required that when a feature was selected to split the training data, the presence of the feature must be associated with the positive data, since we think it is not biologically meaningful to attempt to find common motifs/TFs for the negative genes. As a result, a leaf node with a positive label always appears as a right child of its parent. To learn transcriptional regulatory models, we constructed a training data set for each time point using the TF binding data of the positive and negative genes as described above. We first learned a single decision tree for each time point. To improve the model accuracy, we also learned decision tree ensembles using several methods. Briefly, a decision tree ensemble is set of decision trees, each of which is learned from a modified version of the original training data set. For prediction, an instance is fed to all decision trees and the results from the individual trees are combined by a simple weighted voting scheme, where the weight is the probability of the prediction made by a tree. We used the Bagging [42] and AdaBoost [43] approaches implemented in WEKA [52], using our modified version of C4.5 as the base classifier. As the ratio of negative genes to positive genes is often very large in our case, we also developed a method, called Splitting, to learn decision tree ensembles, again using our modified C4.5 algorithm as the base classifier. The splitting approach works as follows. Given the training data, we first separated it into positive set and negative set. Instances in the negative set were randomly partitioned into *n *subsets, where *n *was chosen so that the size of each negative subset is 3 – 4 times the size of the positive set. This was then repeated 5 times with different random seeds, giving a total of 5*n *negative subsets. We combined each negative subset with the positive set to form a training set and learned a single decision tree for each training set. We combined these trees and the tree learned from the complete training data set to give a total of 5*n*+1 trees to form an ensemble. Overall, each positive gene was used 5*n*+1 times and each negative gene was used 6 times in training, effectively reducing the ratio of negative to positive genes.

### Estimating the model accuracy

A 10-fold cross-validation was used to estimate the accuracy of our method. In other words, we randomly partitioned the training data into 10 subsets of equal size, and then combined 9 subsets for training and the remaining one for testing. The process was repeated 10 times so that each subset was used as a test set once. Furthermore, we repeated the cross-validation procedure 10 times with different random partitioning and calculated the average performance. Denote *TP*, *TN*, *FP*, and *FN *as the numbers of true positive, true negative, false positive and false negative predictions, respectively. The overall accuracy *A *is defined as *A *= (*TP *+ *TN*)/(*TP *+ *FP *+ *TN *+ *FN*). The kappa static *K *[49] is defined as

(1)

where *C *is the expected accuracy that a classifier can achieve by chance, and can be calculated by

(2)

### Extracting significant regulatory rules

For each learned decision tree, we extracted rules by following the branches from the root node to leaf nodes labeled as positive. A node was included in a rule only if its right branch was taken to reach the leaf node of the rule. For example, given a path "Ndd1 ≥ 2.47 ∩ Mcm1 < 3.82 ∩ Fkh1 ≥ 3.44 ⇒ Positive", we will omit the second term and extract a rule "Ndd1 ≥ 2.47 ∩ Fkh1 ≥ 3.44 ⇒ Positive". The reason is that the biological meaning of the second term is ambiguous. We calculated a p-value for each rule with a hypergeometric distribution, and we considered a rule to be significant if its p-value is smaller than 10^-3^. If there are totally *M *positive genes and *N *negative genes, and a rule is supported by *m *positive and *n *negative genes (*m *> *n*), we calculated the p-value for the rule as the probability that we would select at least *m *positive genes if we randomly pick *m *+ *n *gene. This can be calculated as:

(3)

### Combining rule profiles

We converted the time scale for the three expression data sets to a common scale. We used a linear function *T *(*s*) = a * *s *+ *b *for the conversion, where *s *is the actual time in an experiment and *T *(*s*) is its converted time. The coefficients *a*, *b *were obtained from [50]. Using the cell cycle length of CDC28 as a reference, the coefficients are *a *= 0.70 and *b *= -1.58 for CDC15, and *a *= 1.37 and *b *= 5.71 for *α*-factor, meaning that the length of a cell cycle in CDC28 is 0.70-fold of the cell cycle length in CDC15 and 1.37-fold of that in *α*-factor, and the cell cycle in CDC28 starts 1.58 minutes earlier than in CDC15. We then approximated each rule profile with piecewise polynomial functions using the spline function in the MATLAB software. An integrated profile was obtained for each rule by summing its three splines from CDC28, CDC15 and *α*-factor experiments. A rule was considered cell cycle dependent if its integrated profile has two peaks and the distance between the two peaks is approximately 80 – 100 minutes.

### Constructing regulatory networks

The rules with notable cell cycle dependency (in Additional File 4) were used to construct a regulatory network for the yeast cell cycle. By calculating the average distance between two peaks of all the profiles, we estimated the length of a cell cycle to be 85 minutes with CDC28 data set as reference. The period that each rule functions was determined by finding the time points left and right to the peak where the y axis values were two thirds that of the peak. We then plotted the rules in their corresponding functional phases. Next, a subset of the training data with only the genes that are part of some rules in the network were constructed and passed to the decision tree ensembles. If a gene is predicted to be positive, the rules used for the prediction were extracted, and links were created between the rules and the gene.

## Competing interests

The authors declare that they have no competing interests.

## Authors' contributions

JR and WZ conceived of the research. JR designed the study and carried out the computational analysis. JR wrote the paper and WZ helped with the manuscript preparation. YD and EJP helped to improve the algorithm and the manuscript. All authors read and approved the final manuscript.

## Supplementary Material

Additional file 1 This PDF file contains all the significant regulatory rules learned from the CDC28 data set using the ensemble approach. Click here for file

Additional file 2 This PDF file contains all the significant regulatory rules learned from the *α*-factor data set using the ensemble approach. Click here for file

Additional file 3 This PDF file contains all the significant regulatory rules learned from the CDC15 data set using the ensemble approach. Click here for file

Additional file 4 This PDF file contains the integrated rule profiles that show a cell-cycle dependency. Click here for file

Additional file 5 This PDF file contains the integrated rule profiles that do not show a clear cell-cycle dependency. Click here for file
