# Supplementary material for: An ensemble learning approach to reverse-engineering transcriptional regulatory networks from time-series gene expression data
Source: BMC Genomics. 2009 Jul 7;10(Suppl 1):S8. doi: 10.1186/1471-2164-10-S1-S8 (PMC2709269; doi:10.1186/1471-2164-10-S1-S8)
Supplement: Additional file 1 — This PDF file contains all the significant regulatory rules learned from the CDC28 data set using the ensemble approach. [file 1471-2164-10-S1-S8-S1.pdf]

Supplementary Table 1. Regulatory rules learned from CDC28 data set.

10min	p
Swi4 $\geq$ 2.86 $\cap$ Swi6 $\geq$ 3.77	8.45E-13
Mbp1 $\geq$ 4.63	1.82E-12
Swi6 $\geq$ 5.71	2.04E-12
Mbp1 $\geq$ 2.96 $\cap$ Swi4 $\geq$ 4.34	2.41E-11
Swi4 $\geq$ 2.86	2.82E-10

20min	p
Mbp1 $\geq$ 4.63	5.43E-32
Mbp1 $\geq$ 4.63 $\cap$ Swi6 $\geq$ 4.27	4.89E-26
Swi6 $\geq$ 2.67	6.94E-22
Swi4 $\geq$ 2.36 $\cap$ Swi6 $\geq$ 2.96	3.28E-19
Mbp1 $\geq$ 2.3 $\cap$ Swi4 $\geq$ 3.69	2.63E-18
Stb1 $\geq$ 2.43 $\cap$ Swi4 $\geq$ 2.36	3.49E-15
Fkh2 $\geq$ 2.69 $\cap$ Mbp1 $\geq$ 2.3	1.04E-8
Swi4 $\geq$ 3.73	1.15E-7
Dot6 $\geq$ 3.04 $\cap$ Mbp1 $\geq$ 4.63	5.4E-6
Ash1 $\geq$ 3.1 $\cap$ Mbp1 $\geq$ 4.63	5.4E-6
Mbp1 $\geq$ 2.3 $\cap$ Stb1 $\geq$ 2.53	2.7E-5
Hsf1 $\geq$ 3.61 $\cap$ Mbp1 $\geq$ 2.3	2.7E-5
Ecm22 $\geq$ 2.43 $\cap$ Mbp1 $\geq$ 2.3	2.75E-5
Stb1 $\geq$ 2.81	5.21E-4
Mbp1 $\geq$ 2.3 $\cap$ Sok2 $\geq$ 2.5	5.76E-4

30min	p
Mbp1 $\geq$ 4.63	2.19E-17
Swi4 $\geq$ 2.3	6.42E-15
Mbp1 $\geq$ 4.63 $\cap$ Swi6 $\geq$ 4.71	7.16E-15
Swi4 $\geq$ 2.3 $\cap$ Swi6 $\geq$ 2.67	2.73E-13
Mbp1 $\geq$ 2.72 $\cap$ Swi4 $\geq$ 2.94	2.73E-13
Stb1 $\geq$ 2.43 $\cap$ Swi4 $\geq$ 3.69	1.31E-12
Swi6 $\geq$ 4.71	1.74E-12
Fkh2 $\geq$ 2.4 $\cap$ Mbp1 $\geq$ 4.63	1.76E-10
Mbp1 $\geq$ 4.63 $\cap$ Stb1 $\geq$ 2.43	1.91E-10
Mbp1 $\geq$ 6.21 $\cap$ Swi4 $\geq$ 2.3 $\cap$ Swi6 $\geq$ 4.71	2.26E-10
Ash1 $\geq$ 2.73 $\cap$ Swi4 $\geq$ 2.94	4.06E-8
Ash1 $\geq$ 3.41 $\cap$ Mbp1 $\geq$ 4.63	4.56E-7
Stb1 $\geq$ 2.43 $\cap$ Swi4 $\geq$ 2.3 $\cap$ Swi6 $\geq$ 4.71	5.29E-6
Fkh2 $\geq$ 4.51 $\cap$ Swi4 $\geq$ 2.3	9.26E-6
Mbp1 $\geq$ 5.17 $\cap$ Yap6 $\geq$ 2.36	6.25E-5
Fkh2 $\geq$ 3.61	1.22E-4
Met4 $\geq$ 4.14	1.73E-4
Ino2 $\geq$ 2.53 $\cap$ Met4 $\geq$ 4.42	2.92E-4
Cbf1 $\geq$ 3.08 $\cap$ Ino4 $\geq$ 2.3	5.59E-4
Cbf1 $\geq$ 2.63 $\cap$ Mal33 $\geq$ 3.15	5.7E-4
Ino2 $\geq$ 2.44 $\cap$ Swi4 $\geq$ 2.3	8.09E-4

40min	p
Swi4 $\geq$ 4.61	9.94E-18
Fkh2 $\geq$ 3.24	1.02E-11
Mth1 $\geq$ 2.9 $\cap$ Swi4 $\geq$ 4.61	1.97E-11
Swi4 $\geq$ 2.3 $\cap$ Swi6 $\geq$ 2.72	3.58E-10
Fkh2 $\geq$ 2.62 $\cap$ Swi6 $\geq$ 2.35	2.06E-8
Rgm1 $\geq$ 2.3 $\cap$ Swi4 $\geq$ 4.61	3.9E-8
Fkh2 $\geq$ 3.24 $\cap$ Swi4 $\geq$ 3.32	2.43E-7
Mbp1 $\geq$ 5.52 $\cap$ Swi4 $\geq$ 4.61	4.66E-7
Fkh1 $\geq$ 3.0 $\cap$ Fkh2 $\geq$ 2.62	3.76E-6
Ino2 $\geq$ 2.53 $\cap$ Met4 $\geq$ 4.42	5.4E-6
Met4 $\geq$ 4.87	5.45E-6
Fkh2 $\geq$ 2.62 $\cap$ Msn1 $\geq$ 2.32	5.56E-6
Ecm22 $\geq$ 2.34 $\cap$ Swi4 $\geq$ 2.3	5.96E-6
Hsf1 $\geq$ 2.45	2.42E-5
Cbf1 $\geq$ 2.44 $\cap$ Met4 $\geq$ 4.42	6.31E-5
Met31 $\geq$ 3.17 $\cap$ Met4 $\geq$ 3.47	6.44E-5
Cbf1 $\geq$ 3.41 $\cap$ Met31 $\geq$ 3.17 $\cap$ Met4 $\geq$ 3.04	6.57E-5
Stb1 $\geq$ 2.81 $\cap$ Swi4 $\geq$ 2.3	1.31E-4
Fkh2 $\geq$ 4.27 $\cap$ Ndd1 $\geq$ 3.41	2.92E-4
Cbf1 $\geq$ 2.55 $\cap$ Mal33 $\geq$ 3.15	5.93E-4

50min	p
Fkh2 $\geq$ 4.51	1.11E-17
Fkh2 $\geq$ 4.27 $\cap$ Ndd1 $\geq$ 4.34	5.1E-15
Ndd1 $\geq$ 9.12	7.43E-14
Fkh2 $\geq$ 3.35 $\cap$ Swi6 $\geq$ 3.19	7.66E-14
Fkh2 $\geq$ 3.35 $\cap$ Mcm1 $\geq$ 5.01	1.08E-12
Swi6 $\geq$ 3.0	5.65E-12
Swi4 $\geq$ 3.32	5.65E-12
Fkh1 $\geq$ 3.73 $\cap$ Fkh2 $\geq$ 3.35	1.42E-11
Fkh2 $\geq$ 3.35 $\cap$ Swi4 $\geq$ 2.3	1.08E-5
Swi4 $\geq$ 3.32 $\cap$ Swi6 $\geq$ 7.45	6.57E-5
Met4 $\geq$ 3.47	1.23E-4
Cbf1 $\geq$ 2.44 $\cap$ Met4 $\geq$ 3.47	2.95E-4

60min	p
Mcm1 $\geq$ 2.39 $\cap$ Ndd1 $\geq$ 2.47	7.86E-20
Fkh2 $\geq$ 4.2 $\cap$ Ndd1 $\geq$ 2.47	1.31E-16
Ndd1 $\geq$ 2.47	1.17E-15
Fkh2 $\geq$ 4.2 $\cap$ Mcm1 $\geq$ 4.77	3.93E-15
Mcm1 $\geq$ 2.39	8.4E-15
Fkh2 $\geq$ 2.81	5.79E-14
Swi4 $\geq$ 3.73	9.54E-10
Mcm1 $\geq$ 2.44 $\cap$ Swi4 $\geq$ 4.66	5.55E-5
Fkh1 $\geq$ 3.44 $\cap$ Ndd1 $\geq$ 2.47	2.67E-4
Mbp1 $\geq$ 2.32 $\cap$ Swi4 $\geq$ 3.73	2.72E-4
Fkh1 $\geq$ 3.77 $\cap$ Fkh2 $\geq$ 2.3	5.21E-4
Sfl1 $\geq$ 2.58	5.37E-4
Uga3 $\geq$ 2.35	6.43E-4

70min	p
Mcm1 $\geq$ 2.39 $\cap$ Ndd1 $\geq$ 2.3	6.22E-26
Fkh2 $\geq$ 4.07	3.69E-21
Ndd1 $\geq$ 2.3	1.22E-19
Fkh2 $\geq$ 2.3 $\cap$ Ndd1 $\geq$ 2.3	2.9E-17
Mcm1 $\geq$ 2.39	9.15E-17
Fkh2 $\geq$ 3.58 $\cap$ Mcm1 $\geq$ 3.44	2.24E-11
Fkh1 $\geq$ 3.41 $\cap$ Ndd1 $\geq$ 2.3	1.49E-10
Fkh1 $\geq$ 7.24 $\cap$ Fkh2 $\geq$ 4.07	5.55E-5
Nrg1 $\geq$ 2.4 $\cap$ Smp1 $\geq$ 3.12	2.33E-3

80min	p
Mcm1 $\geq$ 2.39 $\cap$ Ndd1 $\geq$ 2.49	2.26E-21
Ndd1 $\geq$ 6.92	7.59E-18
Mcm1 $\geq$ 2.39	4.36E-16
Fkh2 $\geq$ 4.2 $\cap$ Mcm1 $\geq$ 4.77	2.38E-15
Fkh2 $\geq$ 3.24	3.93E-15
Fkh2 $\geq$ 2.6 $\cap$ Ndd1 $\geq$ 2.3	3.93E-15
Fkh1 $\geq$ 2.66 $\cap$ Ndd1 $\geq$ 2.47	9.96E-11
Fkh2 $\geq$ 4.2 $\cap$ Gat1 $\geq$ 2.63	7.03E-4
Fkh2 $\geq$ 3.24 $\cap$ Hsf1 $\geq$ 4.76	7.1E-4
Cbf1 $\geq$ 2.58 $\cap$ Ndd1 $\geq$ 2.3	7.1E-4
Dal82 $\geq$ 2.54 $\cap$ Ndd1 $\geq$ 2.3	7.25E-4

90min	p
Swi4 $\geq$ 2.8	6.24E-12
Ndd1 $\geq$ 2.3	1.04E-11
Fkh2 $\geq$ 3.24 $\cap$ Ndd1 $\geq$ 2.3	1.01E-10
Hsf1 $\geq$ 2.4 $\cap$ Swi4 $\geq$ 2.8	1.44E-10
Fkh2 $\geq$ 3.24	8.21E-10
Fkh2 $\geq$ 3.96 $\cap$ Ino4 $\geq$ 2.3	3.67E-8
Pdr1 $\geq$ 2.33 $\cap$ Smp1 $\geq$ 2.83	4.76E-8
Fhl1 $\geq$ 8.8	4.76E-8
Cup9 $\geq$ 2.81	2.27E-7
Mcm1 $\geq$ 2.3 $\cap$ Ndd1 $\geq$ 2.49	2.36E-7
Fkh1 $\geq$ 2.66 $\cap$ Ndd1 $\geq$ 2.49	2.36E-7
Ash1 $\geq$ 2.36 $\cap$ Swi4 $\geq$ 2.8	2.36E-7
Ndd1 $\geq$ 4.07 $\cap$ Swi4 $\geq$ 2.8	4.26E-7
Fhl1 $\geq$ 8.8 $\cap$ Smp1 $\geq$ 3.02	4.26E-7
Fkh2 $\geq$ 3.96 $\cap$ Rap1 $\geq$ 2.47	4.38E-7
Fhl1 $\geq$ 8.8 $\cap$ Hal9 $\geq$ 2.3	4.43E-7
Ash1 $\geq$ 2.44 $\cap$ Ndd1 $\geq$ 2.81	1.12E-6
Swi5 $\geq$ 5.28	1.17E-6
Smp1 $\geq$ 4.2	1.2E-6
Fkh2 $\geq$ 3.96 $\cap$ Mcm1 $\geq$ 2.38	3.65E-6
Fhl1 $\geq$ 8.8 $\cap$ Hap2 $\geq$ 2.3	5.08E-6
Fkh2 $\geq$ 5.17 $\cap$ Swi4 $\geq$ 2.8	5.34E-6
Cup9 $\geq$ 2.81 $\cap$ Fhl1 $\geq$ 8.8	5.34E-6
Fkh2 $\geq$ 3.96 $\cap$ Met4 $\geq$ 3.38	5.4E-6
Grf10(pho2) $\geq$ 2.32 $\cap$ Ndd1 $\geq$ 2.3	2.47E-5
Phd1 $\geq$ 2.6 $\cap$ Swi4 $\geq$ 2.8	2.52E-5
Mbp1 $\geq$ 5.36 $\cap$ Swi4 $\geq$ 2.8	2.52E-5
Cin5 $\geq$ 2.3 $\cap$ Ndd1 $\geq$ 2.3	2.59E-5
Pdr1 $\geq$ 4.07	2.67E-5
Ino4 $\geq$ 2.3 $\cap$ Ndd1 $\geq$ 2.3	2.67E-5
Mbp1 $\geq$ 3.41 $\cap$ Ndd1 $\geq$ 2.3	2.73E-5
Fkh1 $\geq$ 3.41 $\cap$ Mcm1 $\geq$ 3.12	6.19E-5
Fhl1 $\geq$ 9.25 $\cap$ Ndd1 $\geq$ 2.3	6.19E-5
Ndd1 $\geq$ 4.07 $\cap$ Put3 $\geq$ 2.45	6.25E-5
Hsf1 $\geq$ 4.2 $\cap$ Ndd1 $\geq$ 2.3	6.25E-5
Hsf1 $\geq$ 2.9 $\cap$ Phd1 $\geq$ 2.76 $\cap$ Swi4 $\geq$ 2.8	9.71E-5
Swi4 $\geq$ 2.8 $\cap$ Swi6 $\geq$ 5.71	1.16E-4
Ndd1 $\geq$ 2.3 $\cap$ Swi5 $\geq$ 2.47	1.22E-4
Mbp1 $\geq$ 5.36	1.22E-4
Rap1 $\geq$ 2.51 $\cap$ Smp1 $\geq$ 3.02	1.25E-4
Ndd1 $\geq$ 2.3 $\cap$ Swi6 $\geq$ 3.58	1.25E-4
Mcm1 $\geq$ 4.34	1.7E-4
Cup9 $\geq$ 2.81 $\cap$ Ndd1 $\geq$ 2.3	1.7E-4
Hsf1 $\geq$ 2.9 $\cap$ Phd1 $\geq$ 2.6	5.7E-4
Hap2 $\geq$ 2.67 $\cap$ Pdr1 $\geq$ 2.33	5.7E-4
Hap2 $\geq$ 2.3 $\cap$ Smp1 $\geq$ 2.83	5.76E-4
Fhl1 $\geq$ 8.8 $\cap$ Pdr1 $\geq$ 2.45	5.76E-4
Rap1 $\geq$ 5.26	6.05E-4
Hir2 $\geq$ 2.48	7.93E-4

100min	p
Mbp1 $\geq$ 5.17	1.81E-20
Swi4 $\geq$ 2.47	5.98E-19
Swi6 $\geq$ 3.91	1.32E-15
Swi4 $\geq$ 2.47 $\cap$ Swi6 $\geq$ 3.91	1.46E-14
Mbp1 $\geq$ 2.36 $\cap$ Swi4 $\geq$ 2.92	2.54E-12
Mbp1 $\geq$ 2.3 $\cap$ Swi6 $\geq$ 5.71	1.37E-11
Mal13 $\geq$ 2.83 $\cap$ Mbp1 $\geq$ 5.17	3.11E-9
Stb1 $\geq$ 2.44 $\cap$ Swi4 $\geq$ 2.3	9.52E-9
Ste12 $\geq$ 2.65 $\cap$ Swi4 $\geq$ 2.92	3.75E-8
Fkh2 $\geq$ 2.4 $\cap$ Swi4 $\geq$ 2.92	4.96E-8
Hir2 $\geq$ 2.3 $\cap$ Swi4 $\geq$ 2.92	5.45E-6
Mbp1 $\geq$ 4.83 $\cap$ Mss11 $\geq$ 3.02	5.5E-6
Swi5 $\geq$ 5.65	5.56E-6
Phd1 $\geq$ 4.02 $\cap$ Swi4 $\geq$ 2.92	5.73E-6
Ace2 $\geq$ 2.55 $\cap$ Swi4 $\geq$ 2.3	9.45E-6
Ace2 $\geq$ 2.55 $\cap$ Swi5 $\geq$ 5.65	1.09E-5
Ino4 $\geq$ 2.72 $\cap$ Swi4 $\geq$ 2.3	1.11E-5
Fkh1 $\geq$ 2.56 $\cap$ Swi4 $\geq$ 2.92	2.57E-5
Mbp1 $\geq$ 4.83 $\cap$ Stb1 $\geq$ 2.59	3.07E-5
Hsf1 $\geq$ 2.9 $\cap$ Swi4 $\geq$ 2.92	3.87E-5
Rlm1 $\geq$ 2.43 $\cap$ Swi4 $\geq$ 2.3	1.28E-4
Met4 $\geq$ 3.77	1.75E-4
Mbp1 $\geq$ 4.83 $\cap$ Met4 $\geq$ 2.44	2.98E-4
Met4 $\geq$ 3.77 $\cap$ Swi4 $\geq$ 2.3	5.7E-4
Hir2 $\geq$ 2.3 $\cap$ Phd1 $\geq$ 2.76	5.7E-4
Mbp1 $\geq$ 2.36 $\cap$ Phd1 $\geq$ 3.51	5.81E-4
Gat1 $\geq$ 2.66 $\cap$ Swi4 $\geq$ 2.3	5.93E-4

110min	p
Mbp1 $\geq$ 5.17	1.22E-23
Swi4 $\geq$ 2.3	1.93E-18
Swi6 $\geq$ 2.35	9.55E-18
Mbp1 $\geq$ 2.36 $\cap$ Swi4 $\geq$ 2.34	5.61E-17
Swi4 $\geq$ 2.3 $\cap$ Swi6 $\geq$ 2.35	3.96E-14
Mbp1 $\geq$ 2.96 $\cap$ Swi6 $\geq$ 2.56	2.67E-12
Ash1 $\geq$ 2.54 $\cap$ Mbp1 $\geq$ 5.17	2.65E-10
Stb1 $\geq$ 2.43 $\cap$ Swi4 $\geq$ 2.3	1.79E-9
Mbp1 $\geq$ 2.36 $\cap$ Stb1 $\geq$ 2.43	4.91E-8
Dot6 $\geq$ 2.43 $\cap$ Mbp1 $\geq$ 5.17	5.62E-6
Phd1 $\geq$ 4.02	4.23E-5
Gcn4 $\geq$ 2.54 $\cap$ Phd1 $\geq$ 4.2	6.31E-5
Phd1 $\geq$ 4.02 $\cap$ Swi4 $\geq$ 2.3	1.25E-4
Hsf1 $\geq$ 2.9 $\cap$ Swi4 $\geq$ 2.59	1.31E-4
Mbp1 $\geq$ 2.36 $\cap$ Phd1 $\geq$ 4.02 $\cap$ Swi4 $\geq$ 2.3	3.04E-4
Mbp1 $\geq$ 2.56 $\cap$ Mth1 $\geq$ 2.7	5.81E-4
Met4 $\geq$ 4.42	5.93E-4
Met4 $\geq$ 3.08 $\cap$ Swi4 $\geq$ 2.3	5.93E-4
Mbp1 $\geq$ 2.3 $\cap$ Phd1 $\geq$ 4.2	5.93E-4
Ash1 $\geq$ 2.36 $\cap$ Swi4 $\geq$ 2.92	6.05E-4

120min	p
Swi4 $\geq$ 4.66	1.01E-15
Swi6 $\geq$ 3.0	4.4E-12
Mbp1 $\geq$ 4.14	2.94E-11
Swi4 $\geq$ 2.3 $\cap$ Swi6 $\geq$ 2.35	5.3E-11
Mbp1 $\geq$ 3.12 $\cap$ Swi4 $\geq$ 2.3	9.54E-10
Fkh2 $\geq$ 4.07	9.54E-10
Stb1 $\geq$ 2.43 $\cap$ Swi4 $\geq$ 4.42	2.39E-9
Fkh2 $\geq$ 3.51 $\cap$ Swi4 $\geq$ 2.3	3.16E-8
Ino4 $\geq$ 2.3 $\cap$ Swi4 $\geq$ 4.66	1.61E-7
Mth1 $\geq$ 2.92 $\cap$ Swi4 $\geq$ 4.42	2.22E-7
Ash1 $\geq$ 3.3 $\cap$ Swi4 $\geq$ 2.3	4.55E-6
Hsf1 $\geq$ 3.19 $\cap$ Swi4 $\geq$ 2.3	2.1E-5
Met4 $\geq$ 4.42	2.19E-5
Gcn4 $\geq$ 2.38	3.5E-5
Ino4 $\geq$ 2.43 $\cap$ Met4 $\geq$ 4.42	5.6E-5
Rgm1 $\geq$ 2.38 $\cap$ Swi4 $\geq$ 4.42	5.89E-5
Mcm1 $\geq$ 2.76 $\cap$ Swi4 $\geq$ 2.3	1.12E-4
Ndd1 $\geq$ 4.07	5.21E-4
Fkh2 $\geq$ 2.85 $\cap$ Hsf1 $\geq$ 3.19	5.21E-4
Ste12 $\geq$ 2.38 $\cap$ Swi4 $\geq$ 2.3	5.31E-4
Ndd1 $\geq$ 4.07 $\cap$ Swi4 $\geq$ 2.3	5.31E-4
Met4 $\geq$ 4.42 $\cap$ Swi4 $\geq$ 2.3	7.62E-4

130min	p
Fkh2 $\geq$ 3.51	1.49E-16
Swi4 $\geq$ 9.45	4.02E-16
Fkh2 $\geq$ 3.51 $\cap$ Mcm1 $\geq$ 3.82	1.42E-11
Mbp1 $\geq$ 2.32 $\cap$ Swi4 $\geq$ 2.3	2.33E-10
Swi4 $\geq$ 4.61 $\cap$ Swi6 $\geq$ 2.35	2.47E-10
Fkh2 $\geq$ 3.51 $\cap$ Swi6 $\geq$ 2.33	3.54E-9
Fkh2 $\geq$ 2.4 $\cap$ Ndd1 $\geq$ 4.27	7.05E-9
Fkh2 $\geq$ 3.51 $\cap$ Swi4 $\geq$ 2.3	7.41E-9
Mal13 $\geq$ 2.39 $\cap$ Swi4 $\geq$ 6.81	4.69E-6
Ndd1 $\geq$ 4.74	4.88E-6
Hsf1 $\geq$ 3.32 $\cap$ Mal13 $\geq$ 3.12	2.37E-5
Fkh2 $\geq$ 3.51 $\cap$ Ste12 $\geq$ 3.91	5.66E-5
Fkh2 $\geq$ 3.51 $\cap$ Stb1 $\geq$ 3.54	5.66E-5
Fkh2 $\geq$ 2.85 $\cap$ Mbp1 $\geq$ 8.42	5.66E-5
Met4 $\geq$ 3.65 $\cap$ Swi4 $\geq$ 2.3	5.83E-5
Met4 $\geq$ 4.42	1.08E-4
Mal33 $\geq$ 2.3 $\cap$ Swi4 $\geq$ 2.3	1.14E-4
Ash1 $\geq$ 3.38	1.14E-4
Ndd1 $\geq$ 3.22 $\cap$ Swi4 $\geq$ 2.3	5.16E-4
Hsf1 $\geq$ 2.45 $\cap$ Met4 $\geq$ 3.65	5.37E-4
Ino4 $\geq$ 2.49 $\cap$ Swi4 $\geq$ 2.3	5.59E-4

140min	p
Fkh2 $\geq$ 3.58 $\cap$ Mcm1 $\geq$ 2.44	3.89E-15
Ndd1 $\geq$ 10.13	4.74E-14
Fkh2 $\geq$ 4.07	4.97E-13
Mcm1 $\geq$ 2.39 $\cap$ Ndd1 $\geq$ 2.8	6.78E-13
Mcm1 $\geq$ 2.39	2.3E-12
Swi4 $\geq$ 3.69	8.92E-11
Fkh2 $\geq$ 5.32 $\cap$ Ndd1 $\geq$ 2.3	4.59E-9
Fkh1 $\geq$ 2.66 $\cap$ Fkh2 $\geq$ 2.85	1.33E-7
Mal13 $\geq$ 2.39 $\cap$ Swi4 $\geq$ 3.77	7.6E-7
Met4 $\geq$ 4.66	2.09E-6
Hir2 $\geq$ 2.72 $\cap$ Met4 $\geq$ 4.66	3.84E-6
Hir2 $\geq$ 5.2 $\cap$ Swi4 $\geq$ 3.69	4.97E-5
Ash1 $\geq$ 2.85	6.31E-5
Ino4 $\geq$ 2.3 $\cap$ Swi4 $\geq$ 3.77	9.05E-5
Ino4 $\geq$ 2.43 $\cap$ Met4 $\geq$ 3.38	9.52E-5
Hsf1 $\geq$ 3.35 $\cap$ Swi4 $\geq$ 3.77	9.52E-5
Gcn4 $\geq$ 3.86	9.52E-5
Fkh1 $\geq$ 2.66 $\cap$ Mcm1 $\geq$ 2.39	1.9E-4
Mal13 $\geq$ 3.44	2.34E-4
Mbp1 $\geq$ 2.38 $\cap$ Swi4 $\geq$ 3.77	4.35E-4
Ino4 $\geq$ 2.49 $\cap$ Mal13 $\geq$ 3.44	4.44E-4
Fkh1 $\geq$ 3.44 $\cap$ Ndd1 $\geq$ 2.3	4.44E-4
Met4 $\geq$ 3.65 $\cap$ Swi4 $\geq$ 2.34	4.53E-4
Mcm1 $\geq$ 2.39 $\cap$ Swi4 $\geq$ 2.34	4.53E-4
Fkh2 $\geq$ 2.85 $\cap$ Swi4 $\geq$ 3.69	4.53E-4
Ash1 $\geq$ 2.8 $\cap$ Swi4 $\geq$ 4.61	4.53E-4
Ash1 $\geq$ 3.04 $\cap$ Mal13 $\geq$ 2.39	4.62E-4

150min	p
Mcm1 $\geq$ 2.39 $\cap$ Ndd1 $\geq$ 3.65	2.39E-16
Fkh2 $\geq$ 2.6 $\cap$ Mcm1 $\geq$ 2.39	4.18E-16
Mcm1 $\geq$ 3.65	4.96E-16
Fkh2 $\geq$ 2.6 $\cap$ Mcm1 $\geq$ 2.39 $\cap$ Ndd1 $\geq$ 2.49	5.58E-15
Ndd1 $\geq$ 10.13	6.66E-14
Fkh2 $\geq$ 4.07	2.99E-13
Fkh2 $\geq$ 2.6 $\cap$ Ndd1 $\geq$ 2.47	7.11E-12
Fkh1 $\geq$ 2.66 $\cap$ Mcm1 $\geq$ 2.39	3.69E-10
Gcn4 $\geq$ 5.52	4.11E-9
Swi4 $\geq$ 4.61	7.41E-9
Fkh1 $\geq$ 3.82 $\cap$ Ndd1 $\geq$ 2.49	4.35E-8
Met4 $\geq$ 4.51	9.93E-6
Mth1 $\geq$ 2.92 $\cap$ Swi4 $\geq$ 4.61	2.37E-5
Ash1 $\geq$ 2.54 $\cap$ Mbp1 $\geq$ 2.36	2.42E-5
Ash1 $\geq$ 2.8 $\cap$ Swi4 $\geq$ 9.45	5.66E-5
Ash1 $\geq$ 2.83 $\cap$ Mcm1 $\geq$ 2.76	5.71E-5
Dal81 $\geq$ 8.95	5.77E-5
Mal13 $\geq$ 3.51 $\cap$ Swi4 $\geq$ 6.81	5.83E-5
Ash1 $\geq$ 3.24	9.61E-5
Ash1 $\geq$ 3.24 $\cap$ Ecm22 $\geq$ 2.53	1.14E-4
Ash1 $\geq$ 2.54 $\cap$ Swi6 $\geq$ 2.35	1.14E-4
Hsf1 $\geq$ 3.32 $\cap$ Ino4 $\geq$ 2.43	1.16E-4
Mal33 $\geq$ 2.66 $\cap$ Swi4 $\geq$ 6.81	2.69E-4
Hsf1 $\geq$ 2.45 $\cap$ Met4 $\geq$ 4.51	2.69E-4
Gcn4 $\geq$ 3.86 $\cap$ Swi6 $\geq$ 2.33	5.16E-4
Ndd1 $\geq$ 2.8 $\cap$ Rlm1 $\geq$ 2.66	5.21E-4
Met4 $\geq$ 2.96 $\cap$ Mth1 $\geq$ 2.34	5.21E-4
Hsf1 $\geq$ 3.44 $\cap$ Swi4 $\geq$ 6.81	5.21E-4
Hir2 $\geq$ 3.96	5.21E-4
Grf10(pho2) $\geq$ 2.44 $\cap$ Ndd1 $\geq$ 2.47	5.21E-4
Ash1 $\geq$ 2.8 $\cap$ Phd1 $\geq$ 4.02	5.21E-4
Ndd1 $\geq$ 2.3 $\cap$ Ste12 $\geq$ 2.65	5.31E-4
Hap2 $\geq$ 3.82	5.31E-4
Fkh2 $\geq$ 2.53 $\cap$ Swi4 $\geq$ 4.66	5.31E-4
Cin5 $\geq$ 2.92 $\cap$ Ndd1 $\geq$ 2.3	5.31E-4
Ash1 $\geq$ 2.98 $\cap$ Mth1 $\geq$ 3.04	5.31E-4
Ash1 $\geq$ 2.98 $\cap$ Mal13 $\geq$ 3.61	5.31E-4
Met4 $\geq$ 3.38 $\cap$ Put3 $\geq$ 2.51	5.37E-4
Mcm1 $\geq$ 2.39 $\cap$ Swi6 $\geq$ 2.33	5.37E-4
Ino2 $\geq$ 2.3 $\cap$ Mcm1 $\geq$ 2.39	5.37E-4
Fkh2 $\geq$ 2.6 $\cap$ Mth1 $\geq$ 2.33	5.37E-4
Ash1 $\geq$ 2.98 $\cap$ Ecm22 $\geq$ 2.4 $\cap$ Mth1 $\geq$ 3.04	5.37E-4
Ash1 $\geq$ 2.58 $\cap$ Dal82 $\geq$ 2.54	5.37E-4
Mth1 $\geq$ 5.13	5.48E-4
Cup9 $\geq$ 3.41	6.3E-4
Gcn4 $\geq$ 6.07 $\cap$ Mcm1 $\geq$ 2.39	7.54E-4

160min	p
Ndd1 $\geq$ 2.38	1.36E-14
Mcm1 $\geq$ 4.02	4.65E-14
Fkh1 $\geq$ 3.41 $\cap$ Ndd1 $\geq$ 2.38	6.78E-13
Mcm1 $\geq$ 3.82 $\cap$ Ndd1 $\geq$ 3.73	1.72E-12
Fkh2 $\geq$ 3.24 $\cap$ Ndd1 $\geq$ 2.3	8.81E-10
Fkh2 $\geq$ 9.76 $\cap$ Mcm1 $\geq$ 3.82	1.35E-9
Fkh1 $\geq$ 2.66 $\cap$ Mcm1 $\geq$ 2.39	1.37E-6
Gcn4 $\geq$ 4.34	2.26E-6
Hsf1 $\geq$ 4.61 $\cap$ Skn7 $\geq$ 2.66	4.16E-6
Swi4 $\geq$ 2.34	6.52E-6
Ino4 $\geq$ 2.49 $\cap$ Swi4 $\geq$ 2.34	1.9E-5
Hsf1 $\geq$ 4.61	1.9E-5
Hsf1 $\geq$ 3.44 $\cap$ Ino4 $\geq$ 2.43	1.9E-5
Hsf1 $\geq$ 3.44 $\cap$ Swi4 $\geq$ 2.34	2.02E-5
Hsf1 $\geq$ 4.61 $\cap$ Ino4 $\geq$ 2.43 $\cap$ Met4 $\geq$ 3.65	5.17E-5
Dal81 $\geq$ 8.95	5.38E-5
Ino4 $\geq$ 2.41 $\cap$ Ndd1 $\geq$ 2.3	1E-4
Cup9 $\geq$ 2.3 $\cap$ Ndd1 $\geq$ 2.3	1E-4
Met4 $\geq$ 3.38	1.34E-4
Mal13 $\geq$ 2.39 $\cap$ Swi4 $\geq$ 2.34	4.71E-4
Ace2 $\geq$ 2.34 $\cap$ Swi4 $\geq$ 2.34	4.71E-4
Ndd1 $\geq$ 2.53 $\cap$ Swi4 $\geq$ 2.34	4.95E-4
