# Supplementary material for: An ensemble learning approach to reverse-engineering transcriptional regulatory networks from time-series gene expression data
Source: BMC Genomics. 2009 Jul 7;10(Suppl 1):S8. doi: 10.1186/1471-2164-10-S1-S8 (PMC2709269; doi:10.1186/1471-2164-10-S1-S8)
Supplement: Additional file 2 — This PDF file contains all the significant regulatory rules learned from the α-factor data set using the ensemble approach. [file 1471-2164-10-S1-S8-S2.pdf]

Supplementary Table 2. Regulatory rules learned from α-factor data set.

7min	p
Mbp1 $\geq$ 2.36 $\cap$ Swi4 $\geq$ 2.72	4.88E-6
Mbp1 $\geq$ 2.33	6.08E-6
Swi4 $\geq$ 2.72	8.38E-6
Ndd1 $\geq$ 3.69	2.42E-5
Fkh1 $\geq$ 2.42 $\cap$ Mbp1 $\geq$ 2.33	2.42E-5
Ash1 $\geq$ 3.06	2.52E-5
Swi4 $\geq$ 2.72 $\cap$ Swi6 $\geq$ 4.27	2.98E-5
Mbp1 $\geq$ 5.17 $\cap$ Swi6 $\geq$ 4.27	6.31E-5
Gat1 $\geq$ 2.47 $\cap$ Put3 $\geq$ 2.8	9.23E-5
Swi6 $\geq$ 4.27	9.52E-5
Fkh1 $\geq$ 2.67 $\cap$ Ndd1 $\geq$ 2.86	1.13E-4
Sok2 $\geq$ 3.54	1.21E-4
Fkh1 $\geq$ 2.32 $\cap$ Swi4 $\geq$ 2.72	1.25E-4
Fkh2 $\geq$ 2.38 $\cap$ Ndd1 $\geq$ 2.86	1.72E-4
Yap6 $\geq$ 4.07	5.42E-4
Mbp1 $\geq$ 2.33 $\cap$ Phd1 $\geq$ 2.83	5.42E-4
Mbp1 $\geq$ 2.33 $\cap$ Ndd1 $\geq$ 3.27	5.42E-4
Put3 $\geq$ 2.92	5.53E-4
Phd1 $\geq$ 3.12	5.53E-4
Gal4 $\geq$ 3.06	5.53E-4

14min	p
Mbp1 $\geq$ 6.21	5.98E-19
Mbp1 $\geq$ 2.72 $\cap$ Swi4 $\geq$ 2.34	6.92E-17
Swi4 $\geq$ 2.86	3.22E-15
Mbp1 $\geq$ 6.21 $\cap$ Swi6 $\geq$ 4.27	4.17E-15
Gat3 $\geq$ 8.42	8.57E-15
Swi6 $\geq$ 3.77	4.29E-14
Swi4 $\geq$ 2.86 $\cap$ Swi6 $\geq$ 4.27	2.67E-12
Yap5 $\geq$ 8.18	3.44E-10
Stb1 $\geq$ 2.96 $\cap$ Swi4 $\geq$ 2.86	3.44E-10
Rgm1 $\geq$ 4.02	3.62E-10
Msn4 $\geq$ 4.42	3.62E-10
Ndd1 $\geq$ 3.27 $\cap$ Swi4 $\geq$ 2.86	1.06E-8
Mal13 $\geq$ 2.51 $\cap$ Mbp1 $\geq$ 2.72	5.5E-6
Ecm22 $\geq$ 2.65 $\cap$ Met4 $\geq$ 3.73	6.38E-5
Ino4 $\geq$ 2.49 $\cap$ Met4 $\geq$ 3.73	5.81E-4
Ash1 $\geq$ 2.39 $\cap$ Met4 $\geq$ 3.73	5.81E-4
Phd1 $\geq$ 5.08	5.93E-4
Ace2 $\geq$ 2.43 $\cap$ Swi4 $\geq$ 2.34	5.93E-4
Hsf1 $\geq$ 2.58 $\cap$ Swi4 $\geq$ 2.34	6.05E-4

21min	p
Mbp1 $\geq$ 4.93	4.42E-26
Mbp1 $\geq$ 4.93 $\cap$ Swi6 $\geq$ 4.65	7.24E-19
Swi6 $\geq$ 3.08	2.55E-18
Gat3 $\geq$ 8.42	1.17E-13
Swi4 $\geq$ 2.36 $\cap$ Swi6 $\geq$ 3.08	1.55E-12
Yap5 $\geq$ 8.18	1.9E-9
Stb1 $\geq$ 2.41 $\cap$ Swi6 $\geq$ 3.08	2E-9
Stb1 $\geq$ 3.24 $\cap$ Swi4 $\geq$ 5.3	4.47E-7
Stb1 $\geq$ 3.51	5.56E-6
Swi4 $\geq$ 3.15	9.73E-6
Ino4 $\geq$ 2.59 $\cap$ Met4 $\geq$ 3.82 $\cap$ Put3 $\geq$ 2.85	6.19E-5
Ino4 $\geq$ 2.96 $\cap$ Met4 $\geq$ 3.82 $\cap$ Rox1 $\geq$ 2.5	6.25E-5
Ino4 $\geq$ 2.59 $\cap$ Met4 $\geq$ 3.82	1.23E-4
Met4 $\geq$ 3.82	6.43E-4

28min	p
Mbp1 $\geq$ 2.38 $\cap$ Swi4 $\geq$ 3.73	1.15E-22
Swi4 $\geq$ 3.73	1.02E-18
Mbp1 $\geq$ 2.38	1.66E-17
Stb1 $\geq$ 2.43 $\cap$ Swi4 $\geq$ 3.73	3.16E-16
Swi4 $\geq$ 3.73 $\cap$ Swi6 $\geq$ 2.67	2.05E-15
Mbp1 $\geq$ 2.38 $\cap$ Swi6 $\geq$ 2.67	2.34E-15
Swi6 $\geq$ 3.19	4.29E-14
Mal13 $\geq$ 2.39 $\cap$ Swi4 $\geq$ 3.73	1.31E-11
Met4 $\geq$ 3.73 $\cap$ Swi4 $\geq$ 3.73	2.03E-10
Fkh2 $\geq$ 3.51 $\cap$ Swi4 $\geq$ 3.73	3.49E-8
Ecm22 $\geq$ 2.43 $\cap$ Swi4 $\geq$ 3.73	4.17E-7
Hir2 $\geq$ 5.2 $\cap$ Swi4 $\geq$ 3.73	4.3E-7
Ino4 $\geq$ 2.59 $\cap$ Met4 $\geq$ 3.73	9.28E-7
Ndd1 $\geq$ 4.07 $\cap$ Swi4 $\geq$ 3.73	2.3E-5
Stb1 $\geq$ 3.51 $\cap$ Swi6 $\geq$ 2.67	2.39E-5
Hir1 $\geq$ 3.96 $\cap$ Mbp1 $\geq$ 2.38	6.25E-5
Mbp1 $\geq$ 2.38 $\cap$ Yap1 $\geq$ 2.67	1.21E-4
Fkh2 $\geq$ 2.88 $\cap$ Met4 $\geq$ 3.73	1.21E-4
Met4 $\geq$ 3.73	1.57E-4
Stb1 $\geq$ 3.32	5.59E-4
Hsf1 $\geq$ 3.32 $\cap$ Mbp1 $\geq$ 2.38	5.59E-4
Fkh2 $\geq$ 4.51 $\cap$ Mbp1 $\geq$ 2.38	5.59E-4
Ino4 $\geq$ 2.59 $\cap$ Swi4 $\geq$ 3.73	5.87E-4

35min	p
Swi4 $\geq$ 6.81	1.59E-22
Swi4 $\geq$ 2.94 $\cap$ Swi6 $\geq$ 3.19	1.01E-18
Mbp1 $\geq$ 2.38 $\cap$ Swi4 $\geq$ 2.94	1.9E-16
Stb1 $\geq$ 3.51 $\cap$ Swi4 $\geq$ 2.94	5.97E-14
Hsf1 $\geq$ 3.32 $\cap$ Swi4 $\geq$ 3.73	5.97E-14
Ash1 $\geq$ 2.5 $\cap$ Swi4 $\geq$ 2.94	5.55E-13
Fkh2 $\geq$ 2.4 $\cap$ Swi4 $\geq$ 2.94	6.91E-13
Fkh2 $\geq$ 2.4 $\cap$ Swi4 $\geq$ 2.94 $\cap$ Swi6 $\geq$ 2.35	1.4E-11
Met4 $\geq$ 3.73 $\cap$ Swi4 $\geq$ 2.94	1.93E-10
Ino4 $\geq$ 2.3 $\cap$ Swi4 $\geq$ 2.94	6.49E-8
Ecm22 $\geq$ 2.4 $\cap$ Swi4 $\geq$ 2.94	1.8E-7
Mal33 $\geq$ 2.66 $\cap$ Swi4 $\geq$ 2.94	4.17E-7
Ash1 $\geq$ 2.73 $\cap$ Fkh2 $\geq$ 2.4 $\cap$ Swi4 $\geq$ 2.94	4.17E-7
Put3 $\geq$ 2.85 $\cap$ Swi4 $\geq$ 2.94	4.98E-6
Stb1 $\geq$ 3.51 $\cap$ Swi4 $\geq$ 2.94 $\cap$ Swi6 $\geq$ 2.35	5.24E-6
Hir1 $\geq$ 3.96 $\cap$ Swi4 $\geq$ 2.94	6.01E-5
Gat1 $\geq$ 3.54 $\cap$ Met4 $\geq$ 2.34	6.01E-5
Dal82 $\geq$ 2.58 $\cap$ Swi4 $\geq$ 2.94	6.01E-5
Mal13 $\geq$ 3.38 $\cap$ Swi4 $\geq$ 2.94	1.19E-4
Grf10(pho2) $\geq$ 2.98 $\cap$ Swi4 $\geq$ 2.94	5.48E-4
Fkh1 $\geq$ 3.15 $\cap$ Swi4 $\geq$ 2.94	5.64E-4
Fkh2 $\geq$ 2.72 $\cap$ Met4 $\geq$ 3.73 $\cap$ Swi4 $\geq$ 2.94	7.25E-4
Met4 $\geq$ 6.07 $\cap$ Rox1 $\geq$ 2.6	7.32E-4
Ino2 $\geq$ 2.9 $\cap$ Met4 $\geq$ 6.07	7.32E-4

42min	p
Swi4 $\geq$ 5.6	7.59E-22
Fkh2 $\geq$ 4.07 $\cap$ Ndd1 $\geq$ 2.56	1.1E-19
Fkh2 $\geq$ 3.51	1.13E-17
Fkh2 $\geq$ 4.07 $\cap$ Swi4 $\geq$ 3.77	2.9E-17
Ndd1 $\geq$ 4.34	3.08E-14
Mbp1 $\geq$ 2.81	3.08E-14
Swi4 $\geq$ 2.33 $\cap$ Swi6 $\geq$ 2.35	5.28E-13
Fkh2 $\geq$ 4.07 $\cap$ Mcm1 $\geq$ 4.77	1.25E-12
Ash1 $\geq$ 2.63 $\cap$ Swi4 $\geq$ 2.94	1.25E-12
Fkh2 $\geq$ 2.4 $\cap$ Mbp1 $\geq$ 2.38	4.43E-11
Ash1 $\geq$ 2.3 $\cap$ Fkh2 $\geq$ 2.4	4.43E-11
Mal13 $\geq$ 2.39 $\cap$ Swi4 $\geq$ 5.6	2.24E-10
Ecm22 $\geq$ 2.4 $\cap$ Swi4 $\geq$ 5.6	2.95E-9
Fkh2 $\geq$ 4.07 $\cap$ Hsf1 $\geq$ 3.32	3.67E-8
Ino4 $\geq$ 2.59 $\cap$ Swi4 $\geq$ 2.94	4.14E-8
Hsf1 $\geq$ 3.17 $\cap$ Swi4 $\geq$ 2.94	4.14E-8
Mbp1 $\geq$ 2.55 $\cap$ Swi4 $\geq$ 2.94	4.61E-7
Fkh2 $\geq$ 3.51 $\cap$ Ste12 $\geq$ 3.91 $\cap$ Swi4 $\geq$ 5.6	4.61E-7
Mcm1 $\geq$ 2.39 $\cap$ Ndd1 $\geq$ 2.47	5.45E-6
Hsf1 $\geq$ 3.17 $\cap$ Mal13 $\geq$ 2.66	5.45E-6
Msn4 $\geq$ 2.44 $\cap$ Swi4 $\geq$ 5.6	5.5E-6
Mal33 $\geq$ 2.66 $\cap$ Swi4 $\geq$ 3.77	5.56E-6
Hir2 $\geq$ 5.2	2.47E-5
Met4 $\geq$ 3.51	2.57E-5
Hsf1 $\geq$ 3.17 $\cap$ Mbp1 $\geq$ 2.38	2.65E-5
Hir1 $\geq$ 17.5	6.25E-5
Hir1 $\geq$ 17.5 $\cap$ Met4 $\geq$ 3.51	6.51E-5
Rgm1 $\geq$ 2.3 $\cap$ Swi4 $\geq$ 2.33	1.27E-4
Met4 $\geq$ 3.51 $\cap$ Put3 $\geq$ 3.58	2.86E-4
Ino4 $\geq$ 2.59 $\cap$ Met4 $\geq$ 3.51	5.64E-4
Hir2 $\geq$ 3.0 $\cap$ Swi4 $\geq$ 2.33	5.76E-4

49min	p
Fkh2 $\geq$ 4.98	5.09E-18
Ndd1 $\geq$ 7.85	1.15E-17
Fkh2 $\geq$ 4.98 $\cap$ Mcm1 $\geq$ 3.24	1.24E-11
Fkh2 $\geq$ 2.3 $\cap$ Ndd1 $\geq$ 3.73	2.51E-11
Mcm1 $\geq$ 2.39 $\cap$ Ndd1 $\geq$ 2.81	1.44E-10
Swi4 $\geq$ 2.55	1.15E-8
Mbp1 $\geq$ 2.36	6.11E-8
Ino4 $\geq$ 2.3 $\cap$ Mbp1 $\geq$ 2.36 $\cap$ Swi4 $\geq$ 3.77	3.97E-7
Mbp1 $\geq$ 2.36 $\cap$ Swi4 $\geq$ 2.94	2.14E-5
Ino4 $\geq$ 2.3 $\cap$ Mbp1 $\geq$ 2.36	2.25E-5
Hsf1 $\geq$ 3.44 $\cap$ Swi4 $\geq$ 2.94	2.25E-5
Hsf1 $\geq$ 2.33 $\cap$ Mbp1 $\geq$ 2.36	2.35E-5
Hir2 $\geq$ 2.54	3.3E-5
Ecm22 $\geq$ 2.43 $\cap$ Mbp1 $\geq$ 2.36	6.01E-5
Ash1 $\geq$ 2.63 $\cap$ Fkh2 $\geq$ 4.98	6.38E-5
Ino4 $\geq$ 2.3 $\cap$ Swi4 $\geq$ 6.81	2.86E-4
Met4 $\geq$ 5.08	5.42E-4
Hir2 $\geq$ 3.0 $\cap$ Swi4 $\geq$ 2.55	5.42E-4
Hir2 $\geq$ 3.02 $\cap$ Mbp1 $\geq$ 2.36	5.59E-4
Hir1 $\geq$ 3.96	5.59E-4
Mth1 $\geq$ 5.74	7.03E-4
Ecm22 $\geq$ 2.43 $\cap$ Swi4 $\geq$ 6.81	7.1E-4
Hir1 $\geq$ 3.96 $\cap$ Hir2 $\geq$ 3.0	7.25E-4
Fkh2 $\geq$ 4.98 $\cap$ Hir2 $\geq$ 5.71	7.25E-4
Hir2 $\geq$ 3.0 $\cap$ Ino4 $\geq$ 3.12	7.32E-4

56min	p
Ndd1 $\geq$ 2.8	1.49E-20
Fkh2 $\geq$ 4.61 $\cap$ Ndd1 $\geq$ 2.8	7.31E-19
Mcm1 $\geq$ 2.39 $\cap$ Ndd1 $\geq$ 2.8	6.09E-14
Fkh2 $\geq$ 4.61	6.09E-14
Fkh2 $\geq$ 4.61 $\cap$ Mcm1 $\geq$ 3.02 $\cap$ Ndd1 $\geq$ 2.8	6.01E-5
Cup9 $\geq$ 2.76 $\cap$ Ndd1 $\geq$ 2.8	7.1E-4
Ndd1 $\geq$ 2.8 $\cap$ Sok2 $\geq$ 2.85	7.25E-4
Ino4 $\geq$ 2.81 $\cap$ Met4 $\geq$ 4.14	7.25E-4
Cin5 $\geq$ 4.2 $\cap$ Ndd1 $\geq$ 2.8	7.25E-4
Ash1 $\geq$ 2.5 $\cap$ Met4 $\geq$ 4.14	7.25E-4

63min	p
Ndd1 $\geq$ 3.82	1.09E-10
Mcm1 $\geq$ 3.12 $\cap$ Ndd1 $\geq$ 2.63	1.53E-9
Fkh2 $\geq$ 3.38	3.72E-9
Mcm1 $\geq$ 2.65	7.05E-9
Swi5 $\geq$ 7.4	2.8E-8
Swi4 $\geq$ 2.51	2.48E-7
Fkh2 $\geq$ 4.73 $\cap$ Ndd1 $\geq$ 2.63	3.96E-6
Cin5 $\geq$ 4.2 $\cap$ Ndd1 $\geq$ 2.63	4.74E-6
Mcm1 $\geq$ 2.81 $\cap$ Swi5 $\geq$ 3.41	4.88E-6
Swi4 $\geq$ 2.51 $\cap$ Swi6 $\geq$ 2.35	2.19E-5
Mbp1 $\geq$ 4.27 $\cap$ Swi6 $\geq$ 2.55	2.62E-5
Ndd1 $\geq$ 2.63 $\cap$ Swi6 $\geq$ 3.58	5.21E-4
Cin5 $\geq$ 4.34	5.37E-4
Rim101 $\geq$ 2.3 $\cap$ Swi5 $\geq$ 2.47	5.59E-4
Phd1 $\geq$ 3.3	6.11E-4
Hsf1 $\geq$ 2.92 $\cap$ Swi4 $\geq$ 2.86	7.03E-4
Hir2 $\geq$ 2.7 $\cap$ Ixr1 $\geq$ 2.43	7.03E-4

70min	p
Mbp1 $\geq$ 4.27 $\cap$ Swi4 $\geq$ 2.86	1.57E-19
Swi5 $\geq$ 3.24	4.9E-19
Mbp1 $\geq$ 4.93	2.04E-17
Swi4 $\geq$ 2.86 $\cap$ Swi6 $\geq$ 2.32	6.31E-16
Swi4 $\geq$ 2.51	1.31E-15
Swi6 $\geq$ 4.71	1.04E-13
Mbp1 $\geq$ 2.72 $\cap$ Swi6 $\geq$ 3.58	1.84E-12
Mcm1 $\geq$ 2.3 $\cap$ Swi4 $\geq$ 2.51	4.37E-10
Smp1 $\geq$ 3.47 $\cap$ Swi5 $\geq$ 4.42	4.06E-8
Mcm1 $\geq$ 3.47 $\cap$ Swi6 $\geq$ 3.41	5.42E-8
Mbp1 $\geq$ 2.36 $\cap$ Stb1 $\geq$ 3.02	5.64E-8
Ace2 $\geq$ 2.73 $\cap$ Swi5 $\geq$ 2.92	2.66E-7
Ndd1 $\geq$ 2.63	3.7E-7
Mcm1 $\geq$ 2.62 $\cap$ Swi5 $\geq$ 4.42	4.8E-7
Yap5 $\geq$ 10.52	3.21E-6
Gat3 $\geq$ 9.92	5.56E-6
Mal13 $\geq$ 3.22 $\cap$ Yap5 $\geq$ 10.52	5.67E-6
Ino4 $\geq$ 2.47 $\cap$ Swi5 $\geq$ 6.81	5.67E-6
Stb1 $\geq$ 3.51 $\cap$ Swi6 $\geq$ 3.58	6.02E-6
Stb1 $\geq$ 3.51 $\cap$ Swi4 $\geq$ 4.42	6.02E-6
Fkh2 $\geq$ 8.29	2.89E-5
Msn2 $\geq$ 2.98 $\cap$ Swi5 $\geq$ 4.42	6.51E-5
Gcn4 $\geq$ 2.4 $\cap$ Swi5 $\geq$ 4.42	6.51E-5
Cin5 $\geq$ 2.83 $\cap$ Swi4 $\geq$ 2.51	1.31E-4
Mbp1 $\geq$ 2.36 $\cap$ Mcm1 $\geq$ 4.34	1.34E-4
Ash1 $\geq$ 2.3 $\cap$ Swi5 $\geq$ 2.92	1.34E-4
Ino4 $\geq$ 2.47 $\cap$ Swi4 $\geq$ 4.42 $\cap$ Swi6 $\geq$ 2.32	3.04E-4
Mcm1 $\geq$ 2.39 $\cap$ Ndd1 $\geq$ 2.63	5.99E-4
Fkh2 $\geq$ 8.29 $\cap$ Ndd1 $\geq$ 2.63	5.99E-4
Fkh2 $\geq$ 5.55 $\cap$ Swi4 $\geq$ 2.51	6.05E-4

77min	p
Swi4 $\geq$ 2.47 $\cap$ Swi6 $\geq$ 3.04	8.14E-22
Mbp1 $\geq$ 6.21	1.07E-21
Mbp1 $\geq$ 4.27 $\cap$ Swi6 $\geq$ 2.55	3.6E-20
Swi4 $\geq$ 2.86	1.29E-18
Mbp1 $\geq$ 4.93 $\cap$ Swi4 $\geq$ 5.6	4.15E-17
Stb1 $\geq$ 3.02 $\cap$ Swi4 $\geq$ 2.86	8.66E-15
Swi6 $\geq$ 3.41	1.06E-14
Yap5 $\geq$ 11.56	1.32E-12
Gat3 $\geq$ 8.42	1.47E-11
Mbp1 $\geq$ 4.27 $\cap$ Swi4 $\geq$ 2.86 $\cap$ Swi6 $\geq$ 2.32	4.41E-9
Swi5 $\geq$ 2.85	2.92E-8
Mbp1 $\geq$ 2.36 $\cap$ Stb1 $\geq$ 3.02	4.27E-8
Stb1 $\geq$ 3.3 $\cap$ Swi6 $\geq$ 2.32	4.21E-7
Fkh2 $\geq$ 2.4 $\cap$ Swi4 $\geq$ 2.47	9.17E-6
Gcn4 $\geq$ 2.4 $\cap$ Swi5 $\geq$ 2.85	6.07E-5
Mbp1 $\geq$ 2.36 $\cap$ Sum1 $\geq$ 2.3	1.2E-4
Mcm1 $\geq$ 2.3 $\cap$ Swi4 $\geq$ 2.86	1.25E-4
Ace2 $\geq$ 2.83 $\cap$ Swi5 $\geq$ 7.52	2.8E-4
Mbp1 $\geq$ 2.3 $\cap$ Phd1 $\geq$ 3.12	5.48E-4
Mbp1 $\geq$ 2.36 $\cap$ Swi5 $\geq$ 5.84	5.48E-4
Stb1 $\geq$ 4.42	5.59E-4
Ash1 $\geq$ 3.96 $\cap$ Swi4 $\geq$ 2.47	5.59E-4

84min	p
Swi4 $\geq$ 2.47 $\cap$ Swi6 $\geq$ 2.67	1.83E-25
Swi4 $\geq$ 2.47	2.58E-23
Stb1 $\geq$ 2.43 $\cap$ Swi4 $\geq$ 2.47 $\cap$ Swi6 $\geq$ 2.67	1.18E-20
Mbp1 $\geq$ 2.38 $\cap$ Swi4 $\geq$ 2.47	1.57E-20
Stb1 $\geq$ 3.04 $\cap$ Swi4 $\geq$ 3.77	1.77E-19
Swi6 $\geq$ 2.67	6.11E-19
Mbp1 $\geq$ 2.36	4.47E-18
Ash1 $\geq$ 2.73 $\cap$ Swi4 $\geq$ 2.47	1.88E-12
Mbp1 $\geq$ 2.36 $\cap$ Swi6 $\geq$ 3.24	2.55E-10
Mbp1 $\geq$ 2.36 $\cap$ Stb1 $\geq$ 3.04	3.24E-10
Met4 $\geq$ 2.78 $\cap$ Swi4 $\geq$ 3.77	4.44E-8
Mbp1 $\geq$ 2.36 $\cap$ Met4 $\geq$ 2.78	4.62E-8
Ino4 $\geq$ 2.47 $\cap$ Swi4 $\geq$ 2.47	1.2E-6
Hir1 $\geq$ 3.41 $\cap$ Swi4 $\geq$ 2.86	5.4E-6
Hir2 $\geq$ 2.51 $\cap$ Swi4 $\geq$ 2.86	5.45E-6
Put3 $\geq$ 2.45 $\cap$ Swi4 $\geq$ 3.77	5.56E-6
Swi4 $\geq$ 2.86 $\cap$ Yap1 $\geq$ 2.67	2.7E-5
Met4 $\geq$ 4.51	2.7E-5
Hir2 $\geq$ 5.2	2.7E-5
Swi5 $\geq$ 7.4	3.1E-5
Fkh1 $\geq$ 2.66 $\cap$ Swi4 $\geq$ 2.86 $\cap$ Swi6 $\geq$ 2.32	6.31E-5
Stb1 $\geq$ 3.3 $\cap$ Swi6 $\geq$ 2.32	5.87E-4
Mal13 $\geq$ 2.66 $\cap$ Swi4 $\geq$ 2.47	6.05E-4
Stb1 $\geq$ 3.3	6.49E-4

91min	p
Mbp1 $\geq$ 2.38 $\cap$ Swi4 $\geq$ 3.77	9.72E-21
Swi4 $\geq$ 6.81	3.92E-18
Swi4 $\geq$ 5.3 $\cap$ Swi6 $\geq$ 2.67	1.6E-15
Mbp1 $\geq$ 2.38	3.22E-15
Mbp1 $\geq$ 2.38 $\cap$ Swi4 $\geq$ 3.77 $\cap$ Swi6 $\geq$ 2.67	2.88E-14
Hsf1 $\geq$ 2.49 $\cap$ Mbp1 $\geq$ 2.38	3.08E-14
Stb1 $\geq$ 2.43 $\cap$ Swi4 $\geq$ 5.3	5.07E-13
Ino4 $\geq$ 2.3 $\cap$ Swi4 $\geq$ 3.77	8.42E-12
Ash1 $\geq$ 2.55 $\cap$ Mbp1 $\geq$ 2.38 $\cap$ Swi4 $\geq$ 3.77	1.33E-10
Mbp1 $\geq$ 2.38 $\cap$ Swi6 $\geq$ 2.67	4.42E-10
Hsf1 $\geq$ 2.49 $\cap$ Swi4 $\geq$ 2.72	2.95E-9
Ash1 $\geq$ 2.5 $\cap$ Mbp1 $\geq$ 2.38	1.86E-8
Mbp1 $\geq$ 2.38 $\cap$ Stb1 $\geq$ 3.51	6.54E-7
Hsf1 $\geq$ 3.61 $\cap$ Mbp1 $\geq$ 2.38 $\cap$ Swi4 $\geq$ 3.77	4.51E-6
Mbp1 $\geq$ 2.38 $\cap$ Stb1 $\geq$ 2.43 $\cap$ Swi6 $\geq$ 2.67	4.69E-6
Ino4 $\geq$ 2.59 $\cap$ Mbp1 $\geq$ 2.38 $\cap$ Swi4 $\geq$ 3.77	4.74E-6
Hir1 $\geq$ 17.5	5.6E-5
Hir1 $\geq$ 3.96 $\cap$ Swi4 $\geq$ 3.77	5.83E-5
Mal13 $\geq$ 2.39 $\cap$ Swi4 $\geq$ 2.72	1.01E-4
Mbp1 $\geq$ 2.38 $\cap$ Put3 $\geq$ 2.85	1.06E-4
Met4 $\geq$ 5.13	4.86E-4
Ino4 $\geq$ 2.39 $\cap$ Met4 $\geq$ 2.75	4.86E-4
Hir2 $\geq$ 3.54	4.86E-4
Put3 $\geq$ 2.85 $\cap$ Swi4 $\geq$ 2.72	5.05E-4
Met4 $\geq$ 5.13 $\cap$ Put3 $\geq$ 2.85	5.05E-4
Met4 $\geq$ 3.08 $\cap$ Swi4 $\geq$ 3.77	5.05E-4
Mbp1 $\geq$ 2.38 $\cap$ Met4 $\geq$ 5.08	5.05E-4
Ino4 $\geq$ 2.59 $\cap$ Mbp1 $\geq$ 2.38	5.26E-4
Ino2 $\geq$ 2.98 $\cap$ Put3 $\geq$ 3.58	6.89E-4
Gat1 $\geq$ 2.44 $\cap$ Put3 $\geq$ 3.58	7.1E-4

98min	p
Swi4 $\geq$ 3.77	3.73E-26
Swi4 $\geq$ 3.77 $\cap$ Swi6 $\geq$ 2.35	8.95E-20
Mbp1 $\geq$ 2.38 $\cap$ Swi4 $\geq$ 3.77	2.63E-15
Stb1 $\geq$ 2.43 $\cap$ Swi4 $\geq$ 3.77	4.95E-15
Met4 $\geq$ 3.19 $\cap$ Swi4 $\geq$ 3.77	2.11E-10
Fkh2 $\geq$ 4.07 $\cap$ Swi4 $\geq$ 3.77	3.53E-8
Put3 $\geq$ 2.73 $\cap$ Swi4 $\geq$ 3.77	4.3E-7
Ino4 $\geq$ 2.59 $\cap$ Swi4 $\geq$ 3.77	5.08E-6
Mal13 $\geq$ 3.38 $\cap$ Swi4 $\geq$ 3.77	5.4E-6
Hir1 $\geq$ 17.5	6.25E-5
Ash1 $\geq$ 2.8 $\cap$ Swi4 $\geq$ 3.77 $\cap$ Swi6 $\geq$ 3.19	6.57E-5
Fkh2 $\geq$ 4.51	5.59E-4
Hsf1 $\geq$ 3.61 $\cap$ Swi4 $\geq$ 3.77	5.87E-4
Met4 $\geq$ 5.08	6.96E-4

105min	p
Swi4 $\geq$ 2.47	2.63E-15
Mbp1 $\geq$ 2.38 $\cap$ Swi4 $\geq$ 3.77	8.84E-15
Fkh2 $\geq$ 3.96	1.18E-14
Swi4 $\geq$ 3.32 $\cap$ Swi6 $\geq$ 2.67	4.65E-14
Hsf1 $\geq$ 2.3 $\cap$ Mbp1 $\geq$ 2.38 $\cap$ Swi4 $\geq$ 2.47	5.35E-14
Mbp1 $\geq$ 2.38	1.07E-12
Hsf1 $\geq$ 2.3 $\cap$ Swi4 $\geq$ 2.47	1.33E-10
Hsf1 $\geq$ 2.3 $\cap$ Mbp1 $\geq$ 2.36	8.13E-10
Mbp1 $\geq$ 2.36 $\cap$ Swi6 $\geq$ 2.67	9.61E-9
Ash1 $\geq$ 3.41 $\cap$ Swi4 $\geq$ 2.47	1.9E-8
Fkh2 $\geq$ 3.35 $\cap$ Swi4 $\geq$ 2.47	2.75E-8
Ndd1 $\geq$ 7.85	3.89E-7
Hir1 $\geq$ 3.96 $\cap$ Swi4 $\geq$ 2.47	3.89E-7
Hir2 $\geq$ 5.2	4.05E-7
Ash1 $\geq$ 3.41 $\cap$ Mbp1 $\geq$ 2.36	8.74E-7
Met4 $\geq$ 4.78 $\cap$ Swi4 $\geq$ 2.47	4.98E-6
Hir2 $\geq$ 3.0 $\cap$ Swi4 $\geq$ 2.47	2.21E-5
Hir1 $\geq$ 3.73	2.21E-5
Hir1 $\geq$ 2.86 $\cap$ Mbp1 $\geq$ 2.36	2.3E-5
Swi6 $\geq$ 7.45	6.13E-5
Fkh2 $\geq$ 2.58 $\cap$ Ndd1 $\geq$ 3.27	1.09E-4
Mcm1 $\geq$ 2.81	1.14E-4
Mal13 $\geq$ 2.66 $\cap$ Swi4 $\geq$ 2.47	1.14E-4
Ash1 $\geq$ 3.73 $\cap$ Swi4 $\geq$ 2.47 $\cap$ Swi6 $\geq$ 2.72	2.8E-4
Fkh2 $\geq$ 2.94 $\cap$ Mcm1 $\geq$ 2.81	5.48E-4
Ecm22 $\geq$ 2.3 $\cap$ Mbp1 $\geq$ 2.36	5.48E-4
Phd1 $\geq$ 2.83 $\cap$ Swi4 $\geq$ 2.47	7.03E-4
Smp1 $\geq$ 2.3 $\cap$ Swi4 $\geq$ 5.6	7.1E-4
Rlm1 $\geq$ 3.38 $\cap$ Swi4 $\geq$ 2.47	7.1E-4
Gat1 $\geq$ 3.86 $\cap$ Ino2 $\geq$ 2.44	7.1E-4

112min	p
Ndd1 $\geq$ 4.79	2.33E-21
Fkh2 $\geq$ 4.73	1.34E-19
Swi4 $\geq$ 2.55	5.69E-11
Mcm1 $\geq$ 2.39 $\cap$ Ndd1 $\geq$ 3.22	2.3E-9
Mbp1 $\geq$ 2.38 $\cap$ Swi4 $\geq$ 3.77	2.49E-9
Fkh2 $\geq$ 4.07 $\cap$ Mcm1 $\geq$ 4.77	2.95E-8
Hir2 $\geq$ 5.2	3.22E-8
Hir1 $\geq$ 3.73 $\cap$ Swi4 $\geq$ 2.55	4.13E-7
Hir1 $\geq$ 3.73	4.33E-6
Hir1 $\geq$ 3.73 $\cap$ Hir2 $\geq$ 2.7	4.55E-6
Hir2 $\geq$ 2.7 $\cap$ Swi4 $\geq$ 2.55	2.21E-5
Met4 $\geq$ 4.14	2.3E-5
Hir2 $\geq$ 2.7 $\cap$ Mbp1 $\geq$ 2.36	2.3E-5
Ash1 $\geq$ 3.51 $\cap$ Fkh2 $\geq$ 4.07	5.95E-5
Mal13 $\geq$ 3.58 $\cap$ Swi4 $\geq$ 6.81	6.07E-5
Ino4 $\geq$ 3.12 $\cap$ Put3 $\geq$ 2.73	1.09E-4
Fkh2 $\geq$ 4.07 $\cap$ Swi4 $\geq$ 3.77	1.14E-4
Ino4 $\geq$ 3.1	1.41E-4
Put3 $\geq$ 3.15	5.48E-4
Fkh2 $\geq$ 4.07 $\cap$ Hir1 $\geq$ 3.41	7.03E-4
Fkh2 $\geq$ 4.07 $\cap$ Put3 $\geq$ 2.73	7.1E-4
Hir2 $\geq$ 2.7 $\cap$ Ino2 $\geq$ 2.44	7.17E-4

119min	p
Ndd1 $\geq$ 7.85	1.6E-15
Fkh2 $\geq$ 2.3 $\cap$ Ndd1 $\geq$ 2.63	5.63E-11
Mcm1 $\geq$ 2.39 $\cap$ Ndd1 $\geq$ 2.63	3.96E-10
Fkh2 $\geq$ 3.96	4.42E-10
Cup9 $\geq$ 2.3 $\cap$ Ndd1 $\geq$ 2.63	1.01E-4
Dot6 $\geq$ 2.33 $\cap$ Ndd1 $\geq$ 2.63	5.05E-4
Mcm1 $\geq$ 2.39 $\cap$ Ndd1 $\geq$ 2.63 $\cap$ Sok2 $\geq$ 2.85	6.96E-4
Hir2 $\geq$ 2.7 $\cap$ Mbp1 $\geq$ 2.36	6.96E-4
Ndd1 $\geq$ 2.63 $\cap$ Sko1 $\geq$ 2.3	7.03E-4
Ndd1 $\geq$ 2.63 $\cap$ Sfl1 $\geq$ 2.58	7.03E-4
Ndd1 $\geq$ 2.63 $\cap$ Rlm1 $\geq$ 2.59	7.03E-4
Mcm1 $\geq$ 2.39 $\cap$ Sok2 $\geq$ 2.85	7.03E-4
