# Supplementary figures and images for: An ensemble learning approach to reverse-engineering transcriptional regulatory networks from time-series gene expression data
Source: BMC Genomics. 2009 Jul 7;10(Suppl 1):S8. doi: 10.1186/1471-2164-10-S1-S8 (PMC2709269; doi:10.1186/1471-2164-10-S1-S8)

**Supplementary Figure 1.** Rule profiles that show cell-cycle dependency.

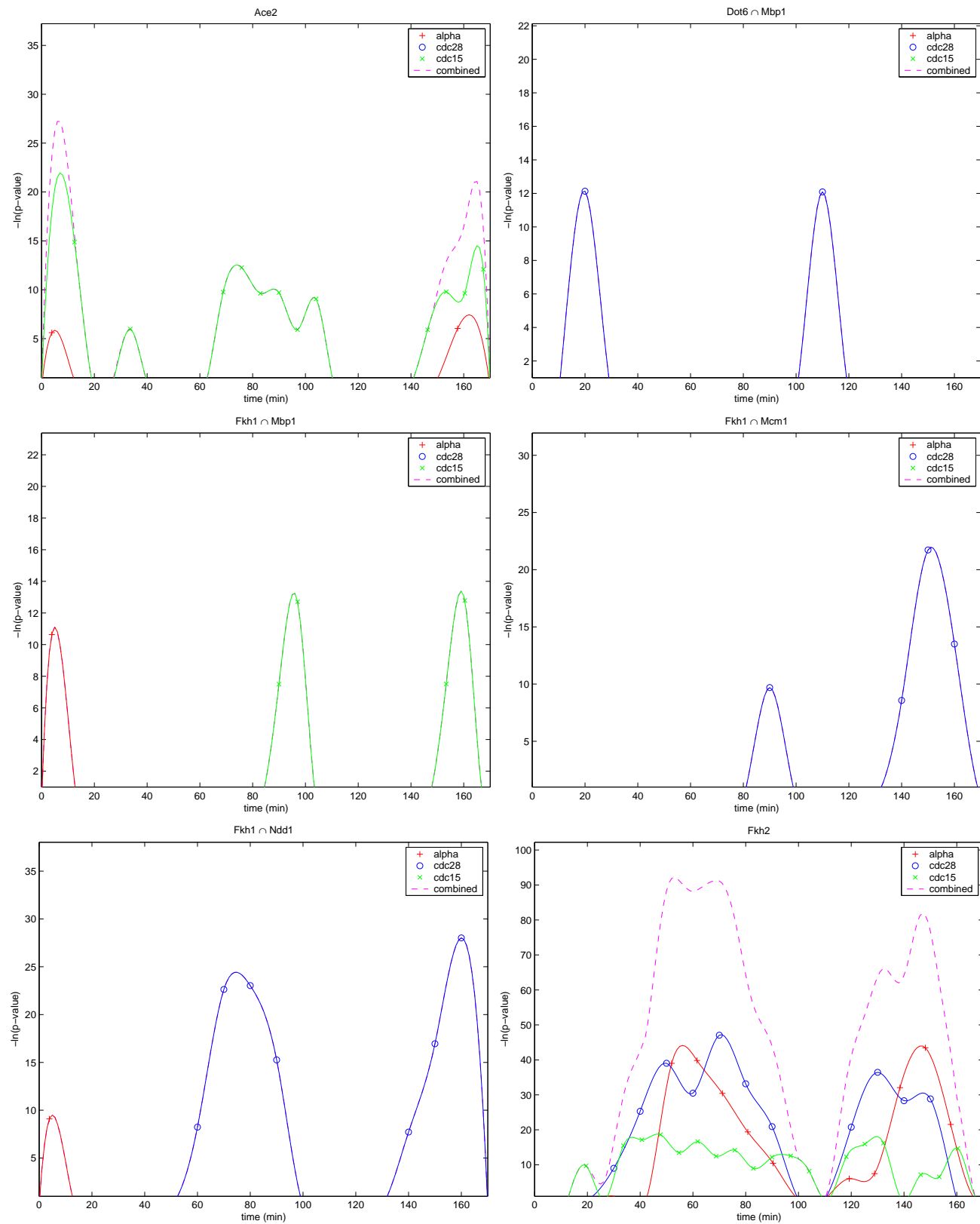

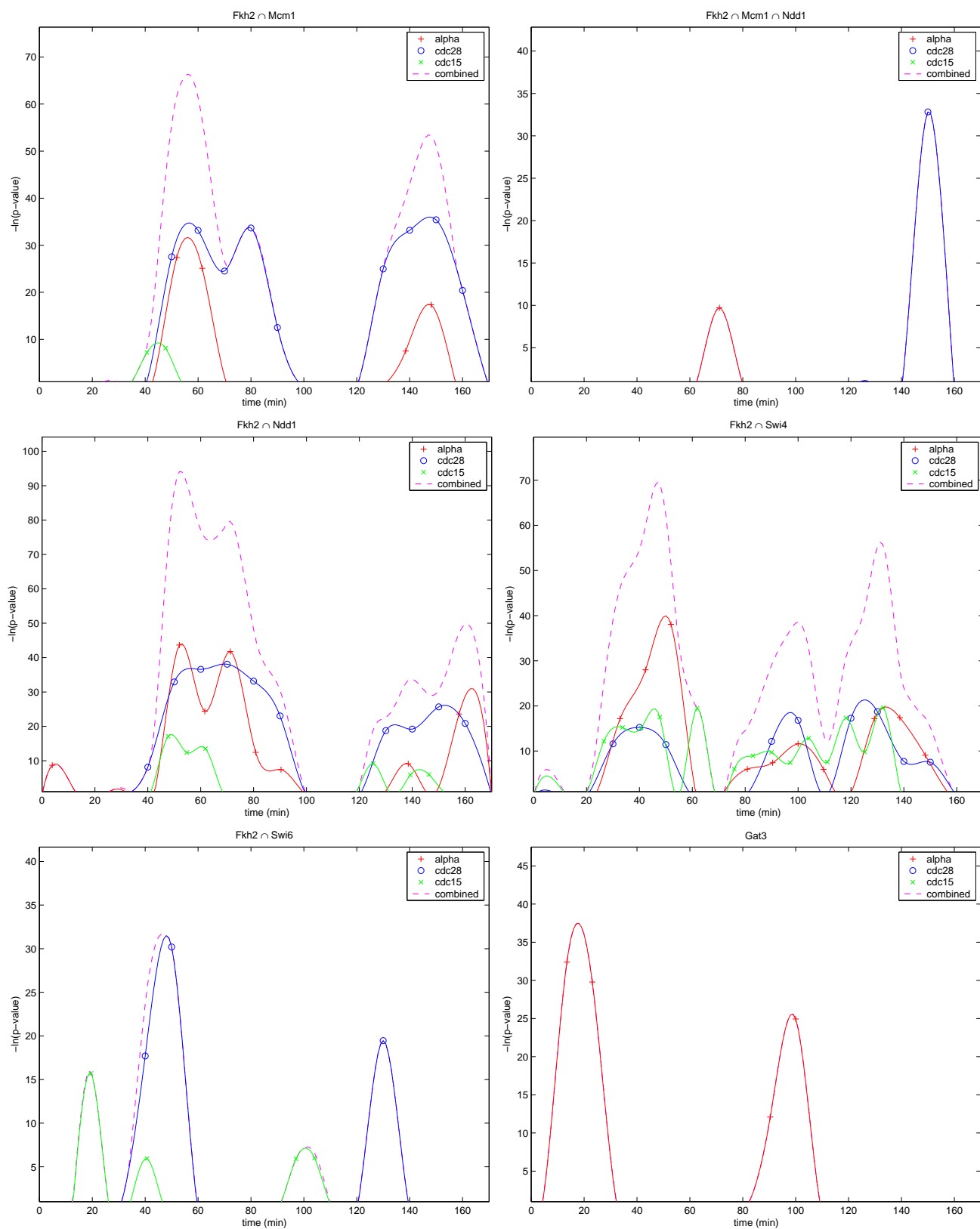

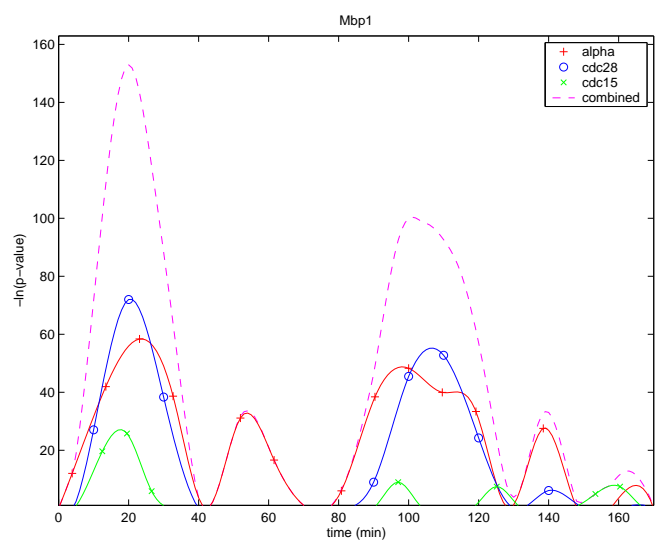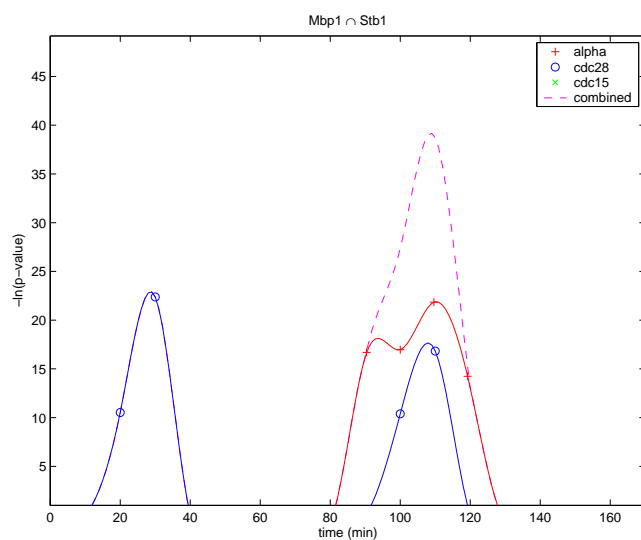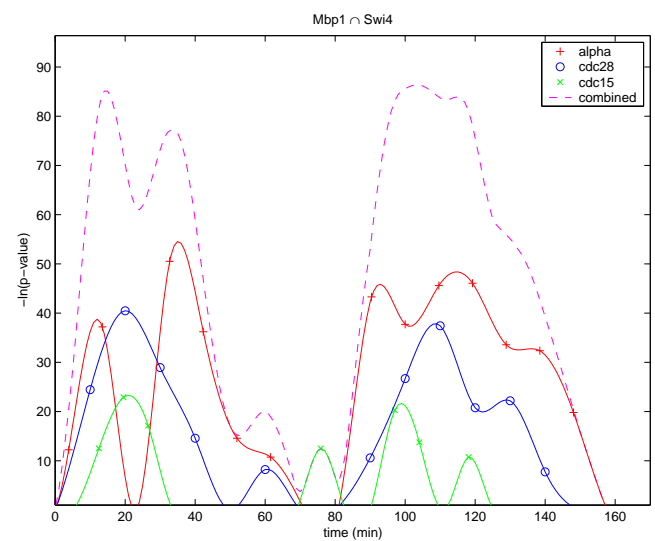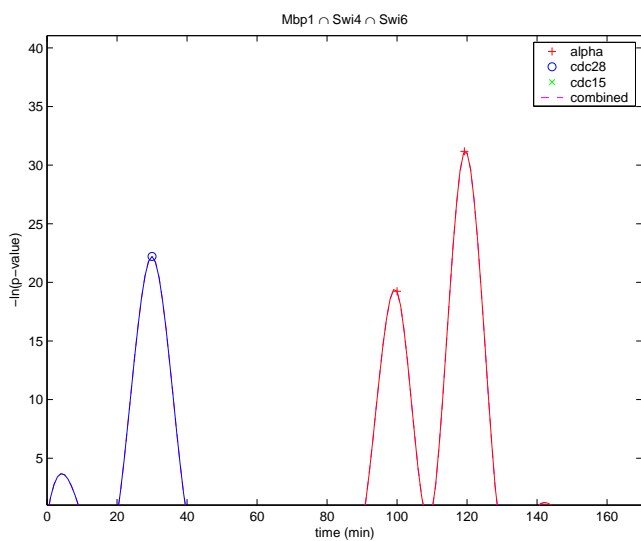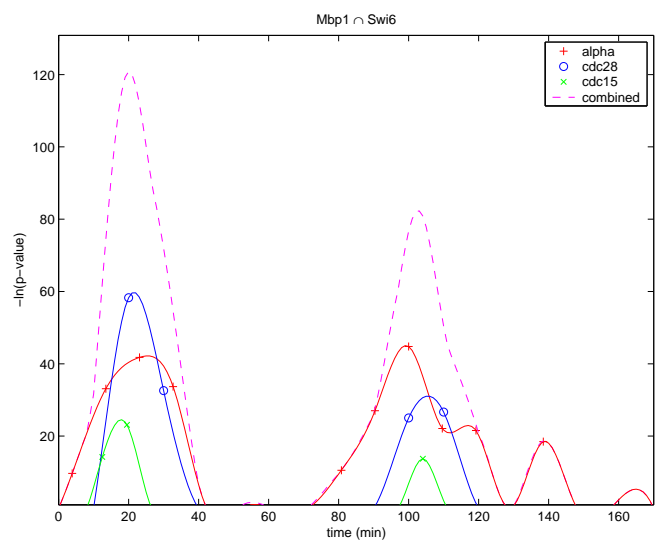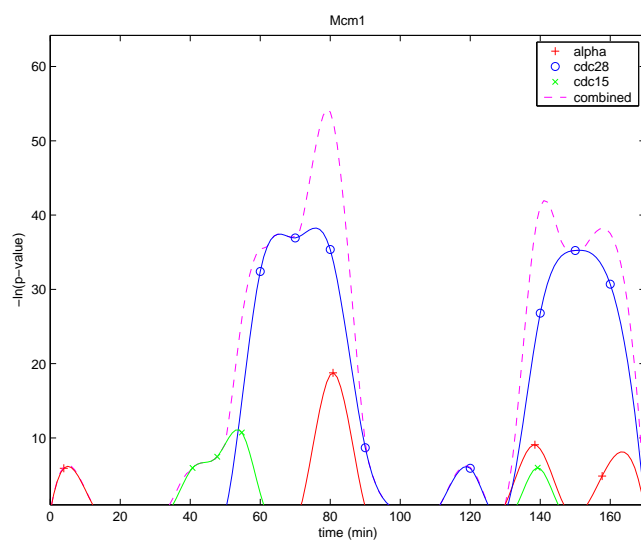

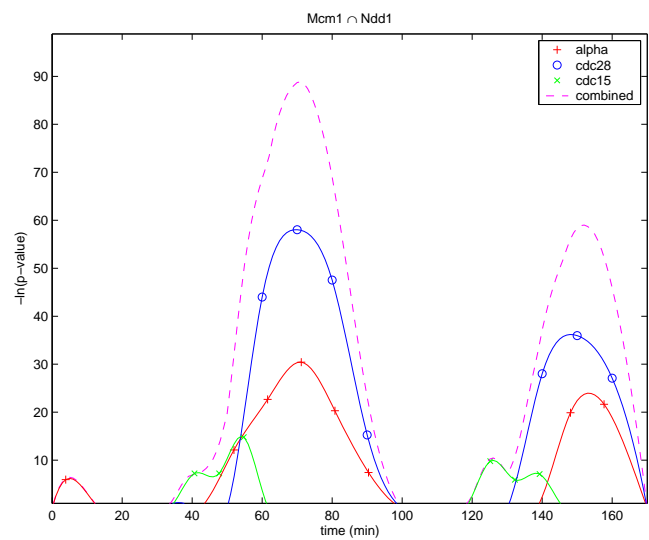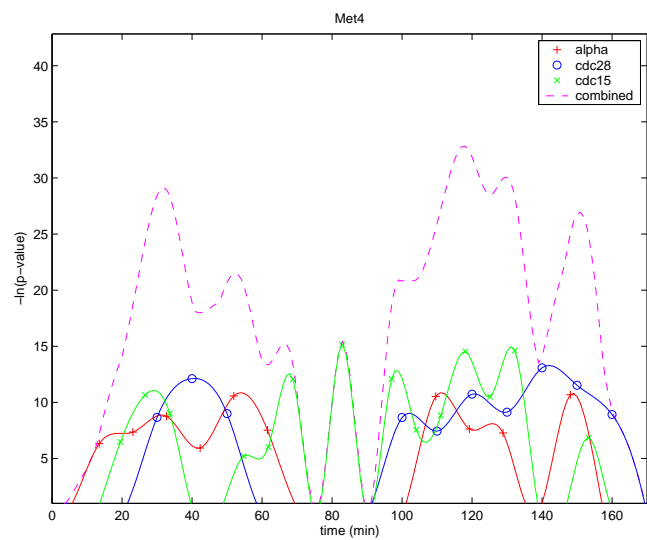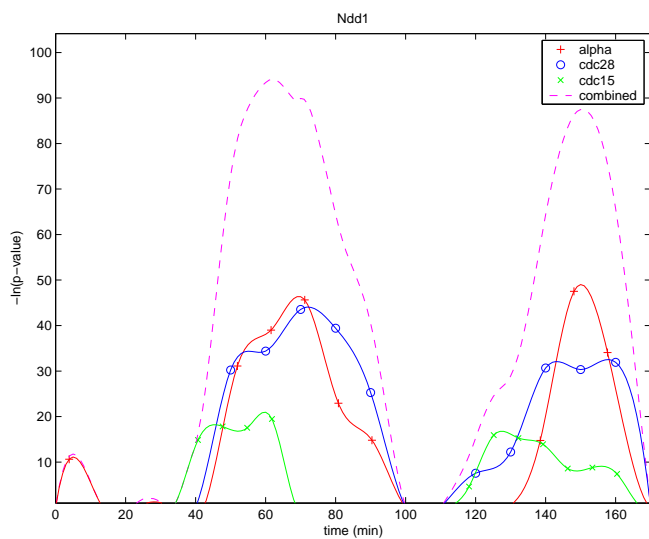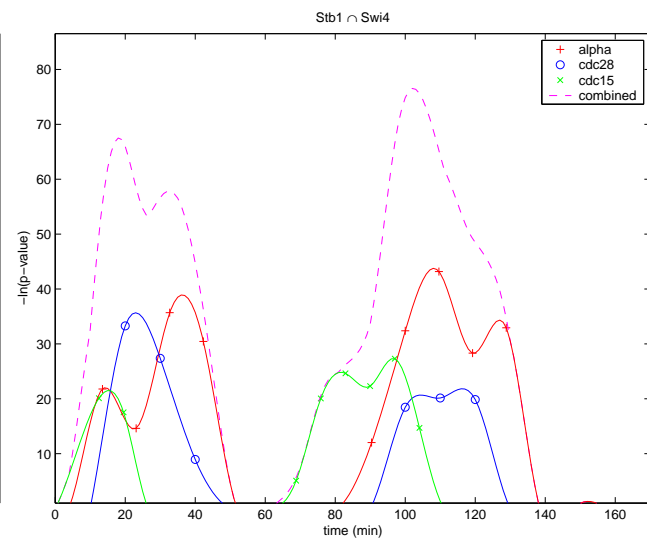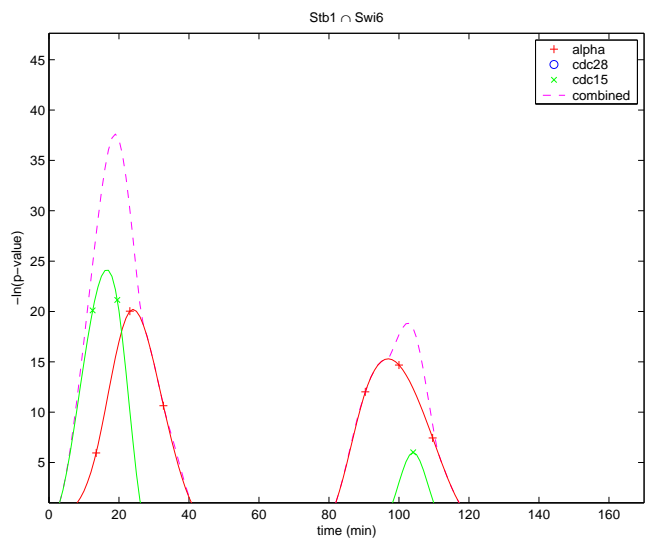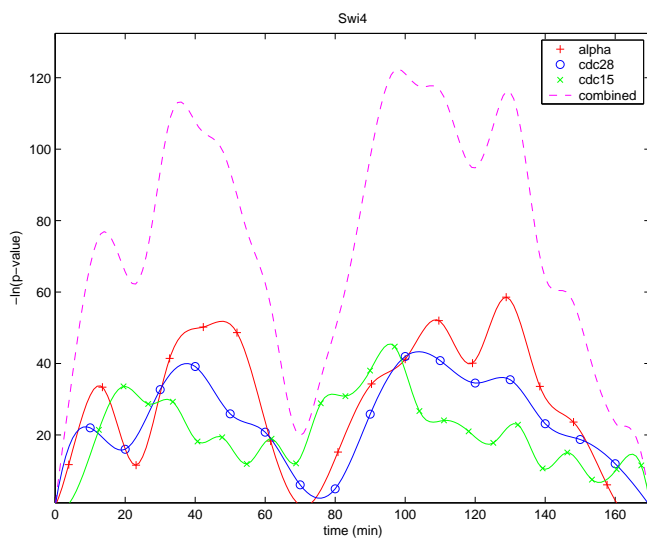

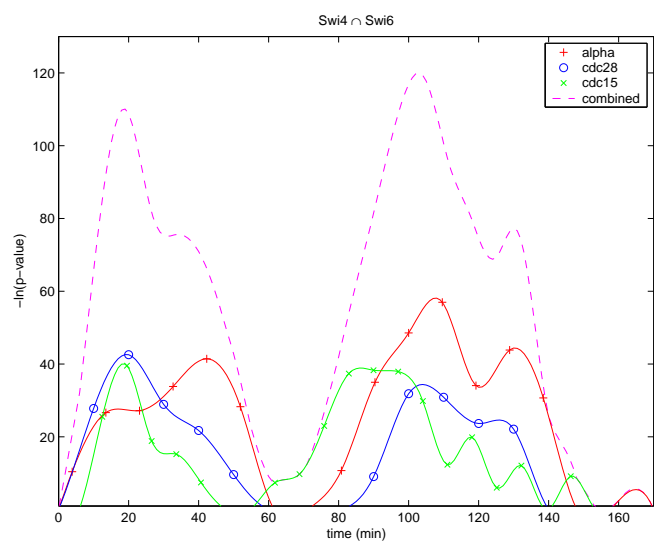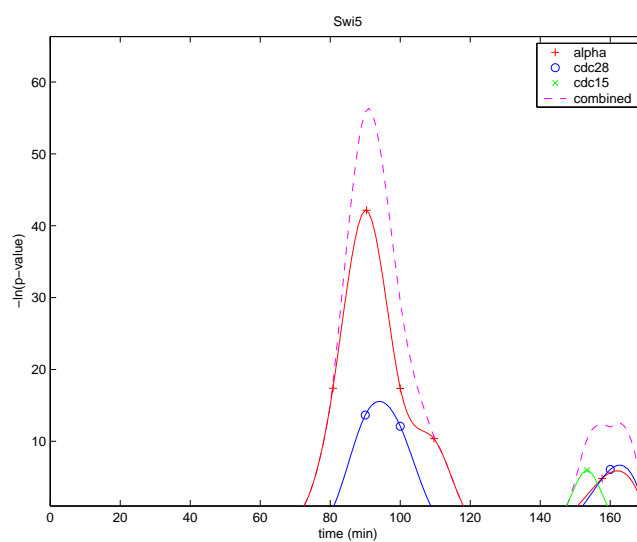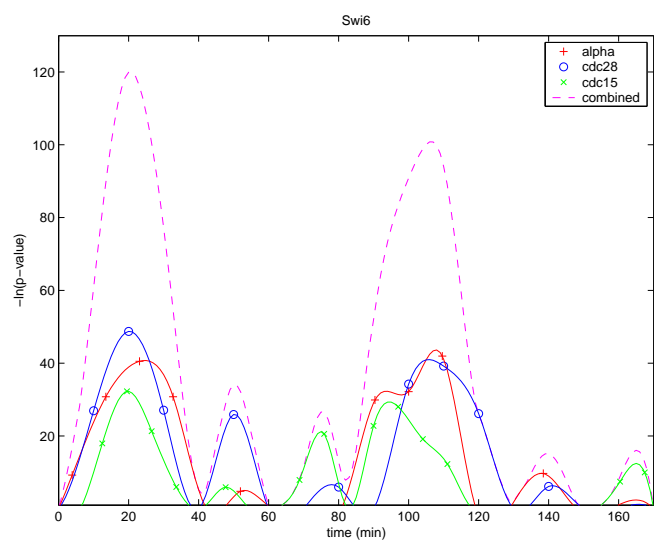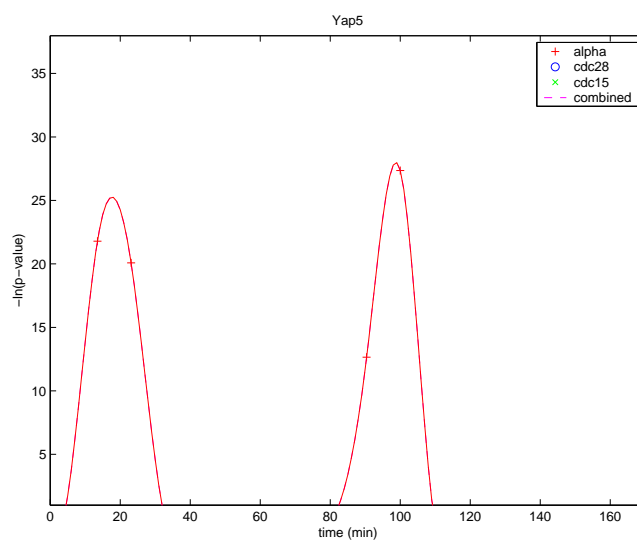

Supplement: Additional file 3 — This PDF file contains all the significant regulatory rules learned from the CDC15 data set using the ensemble approach. [file 1471-2164-10-S1-S8-S3.pdf]

**Supplementary Figure 2.** Rule profiles that do not show clear cell-cycle dependency.

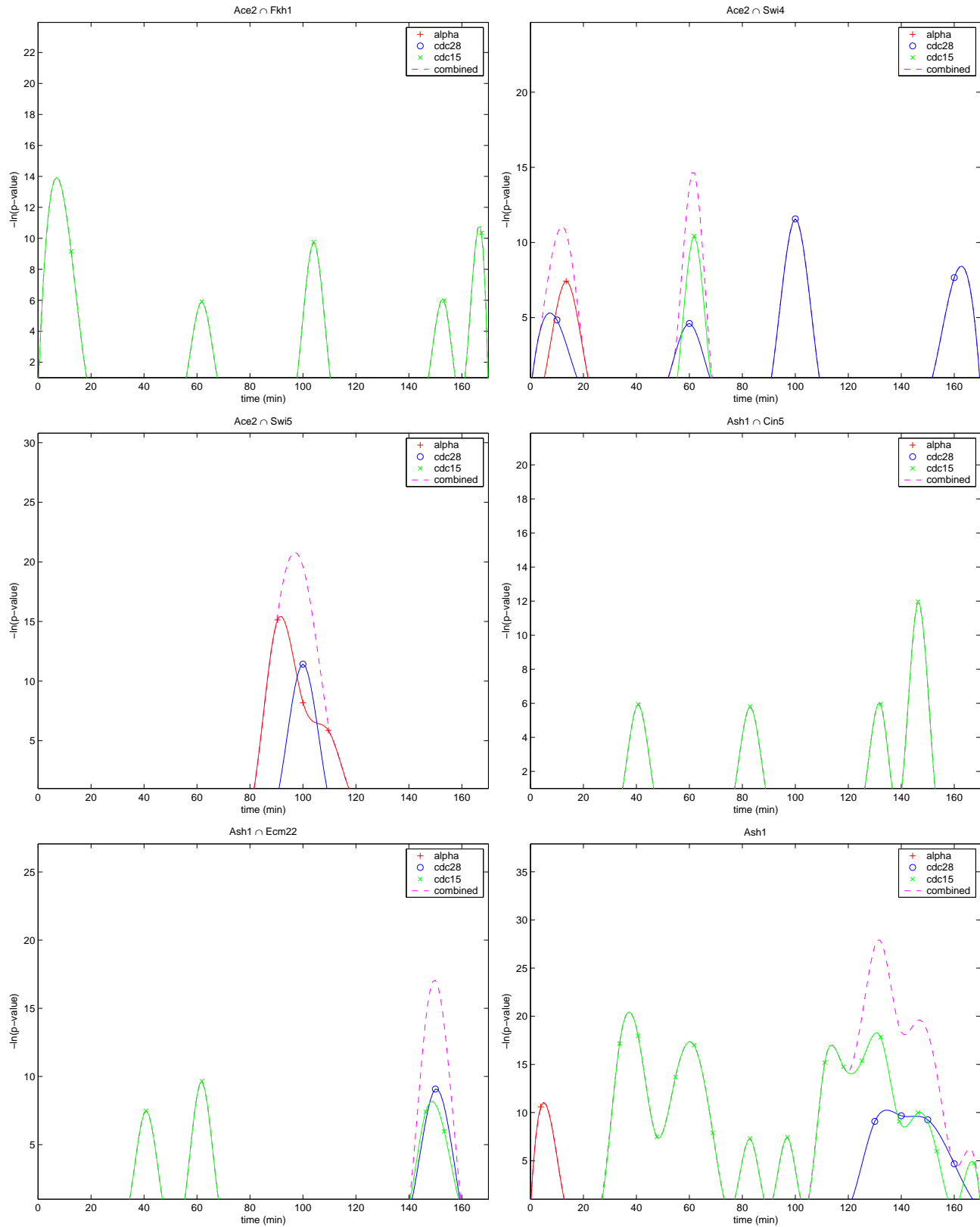

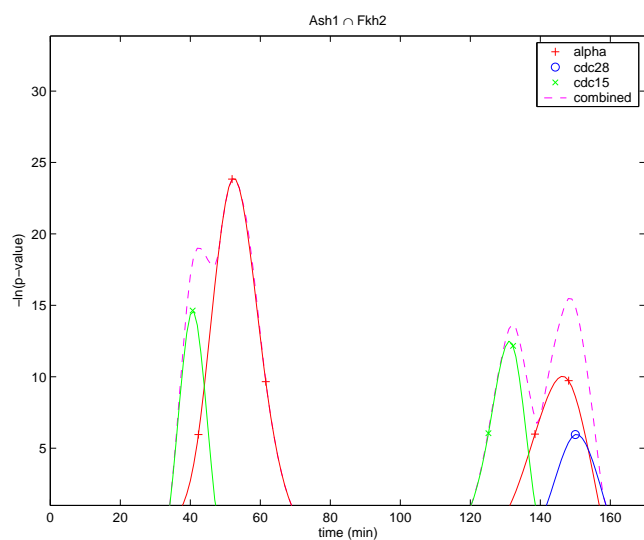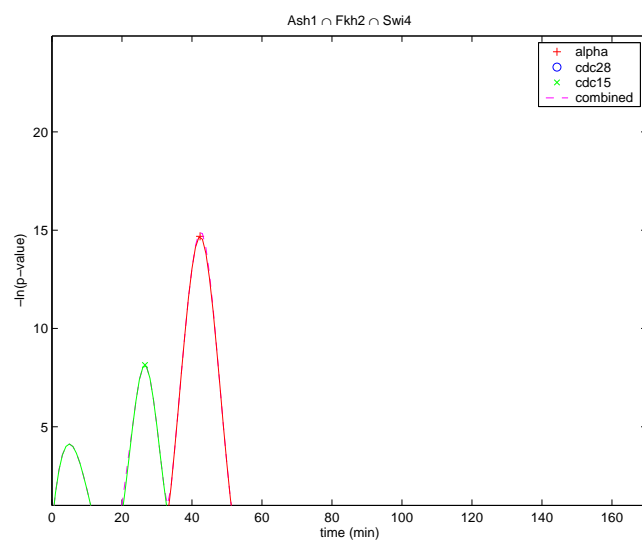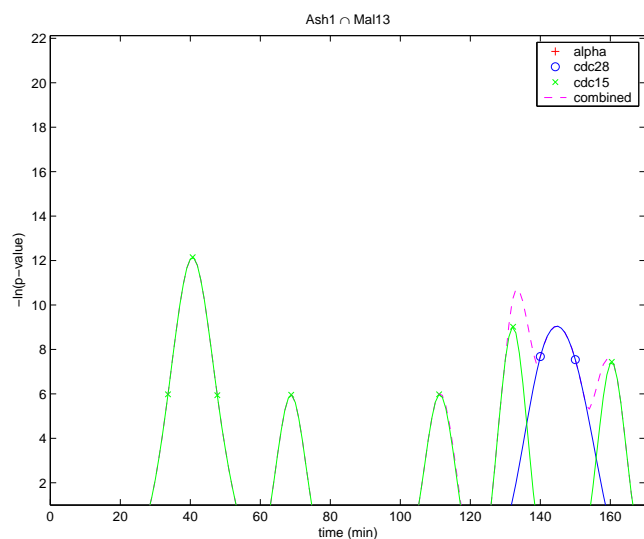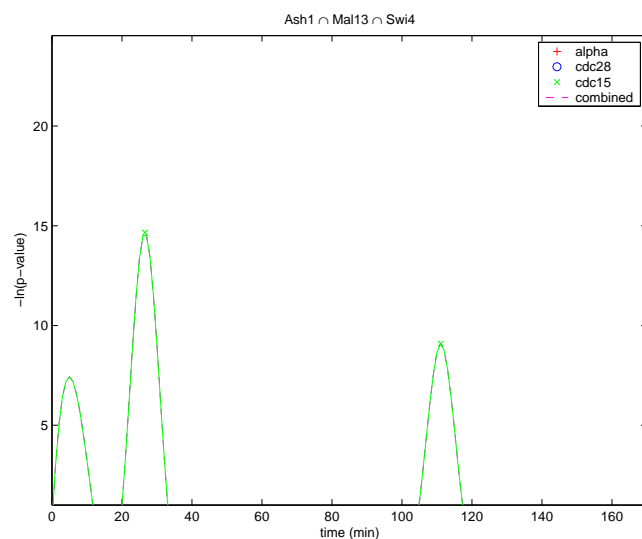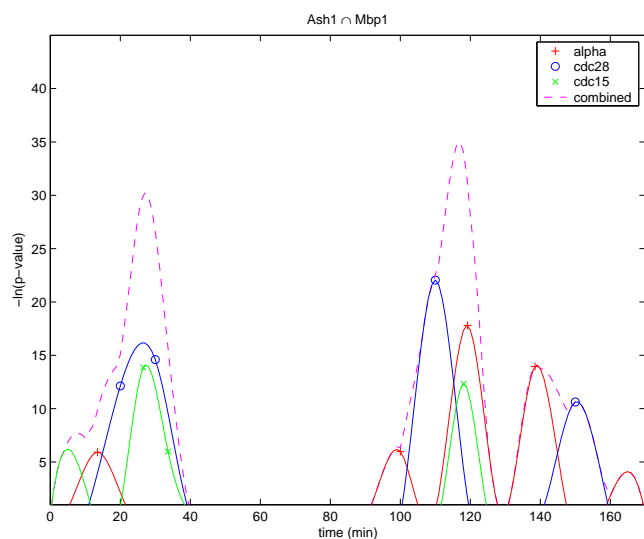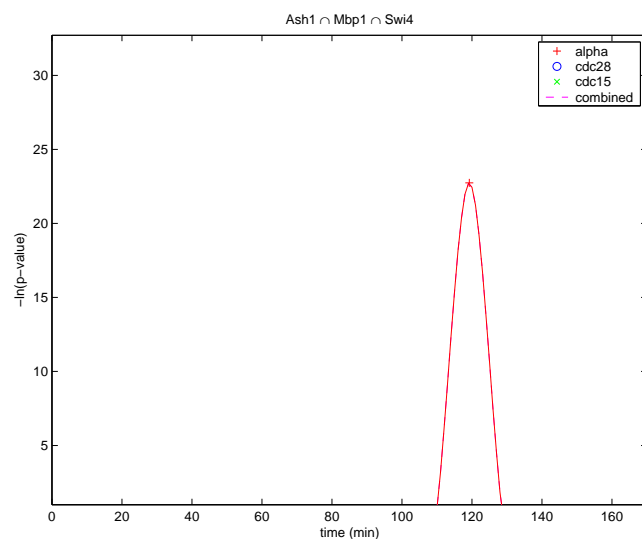

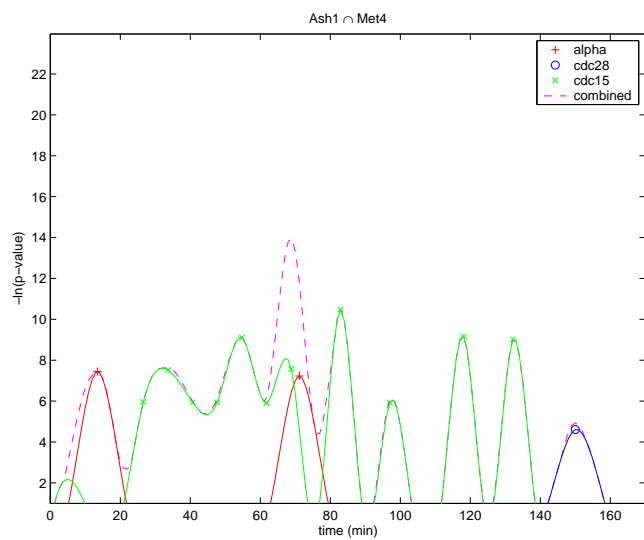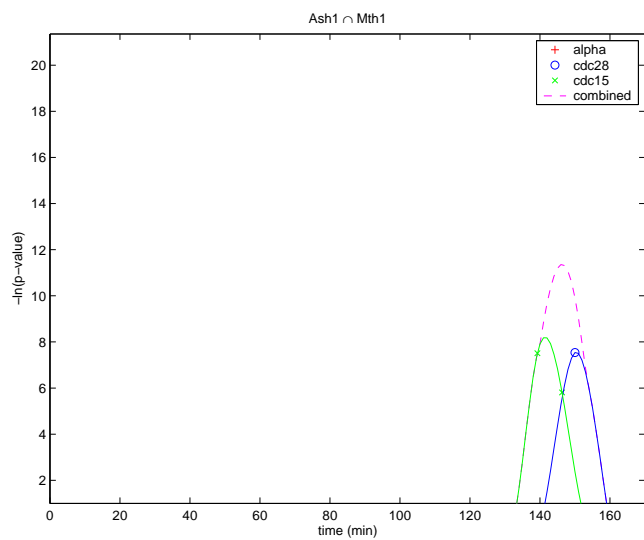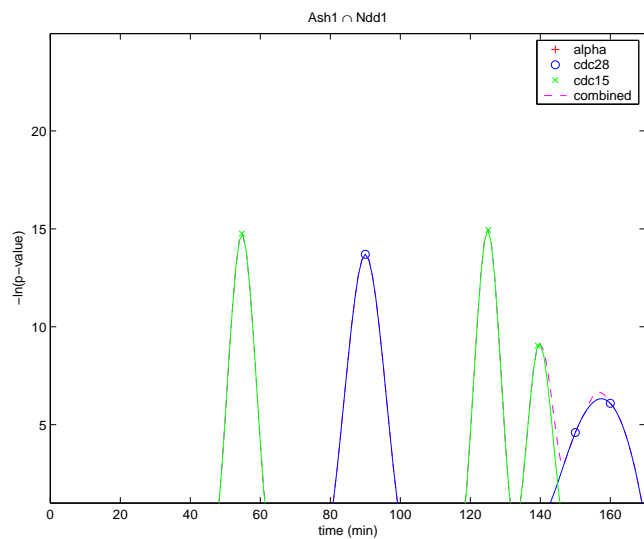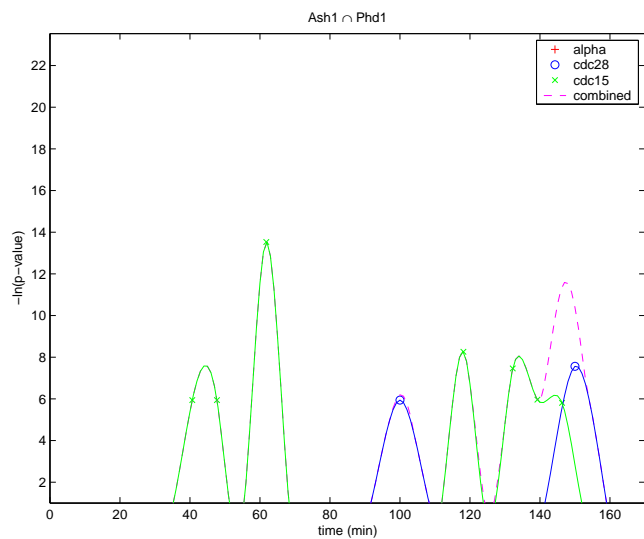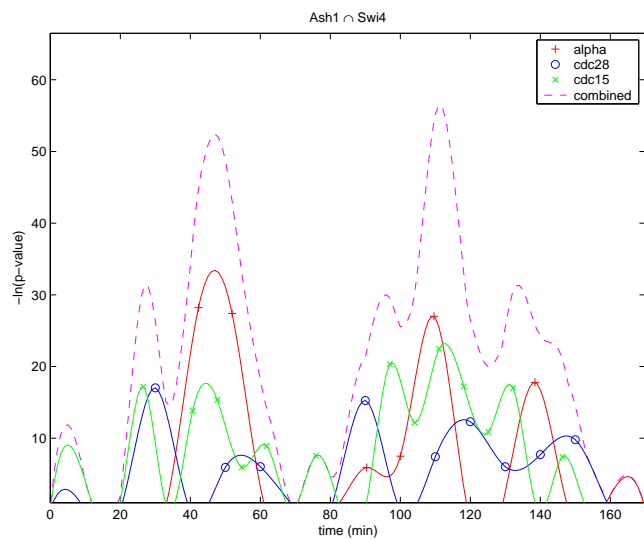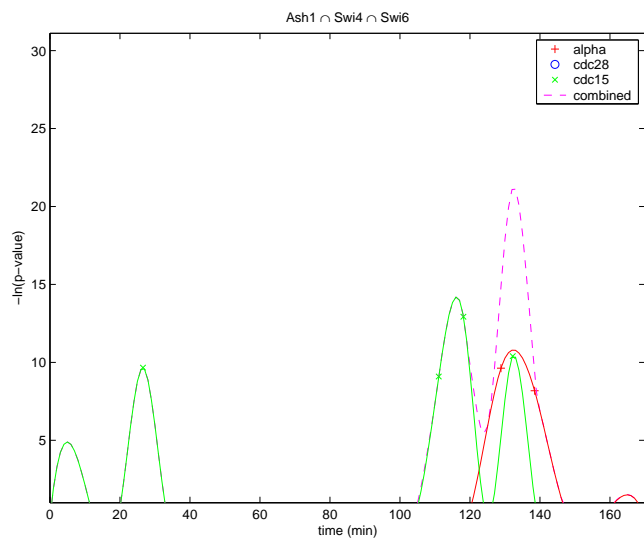

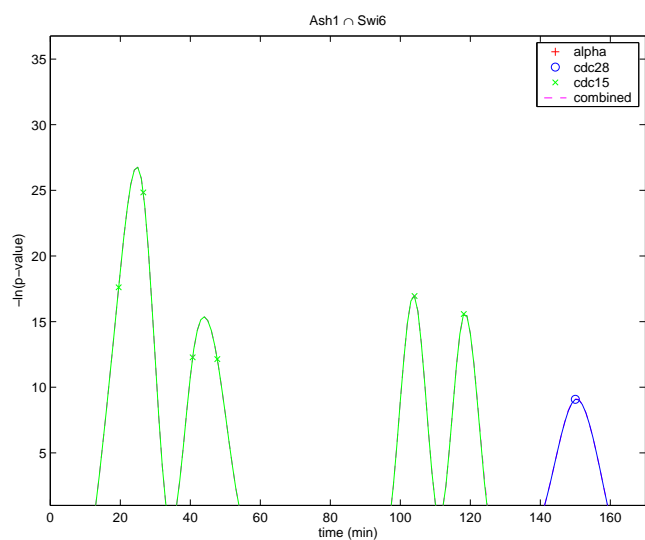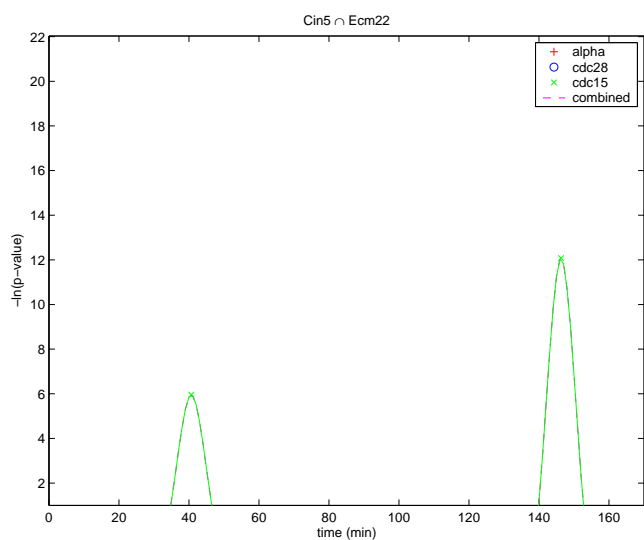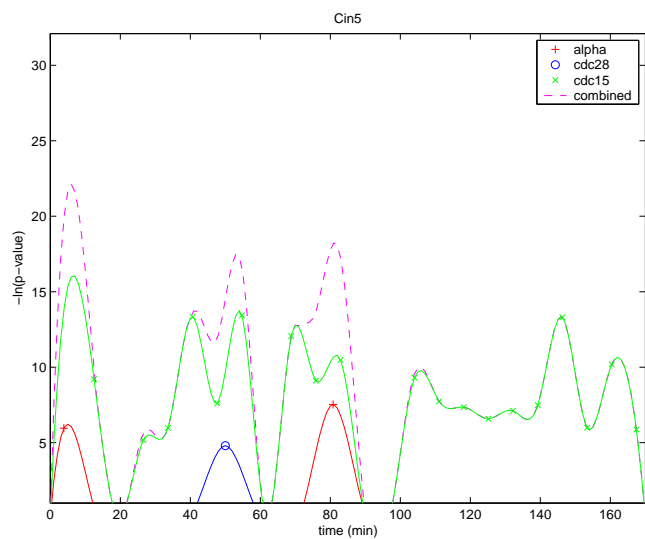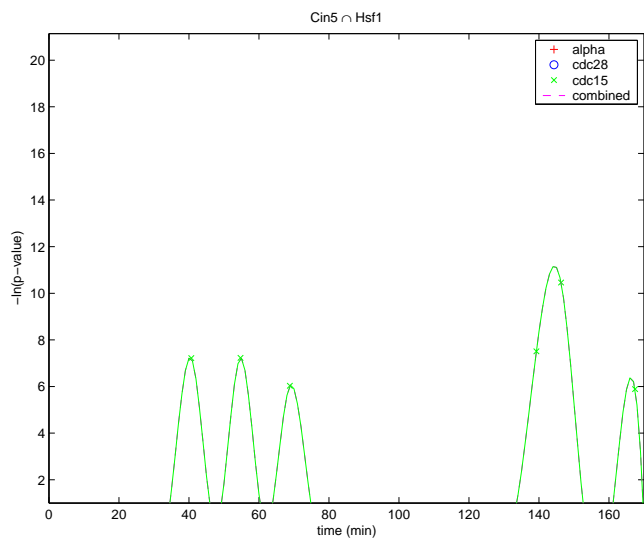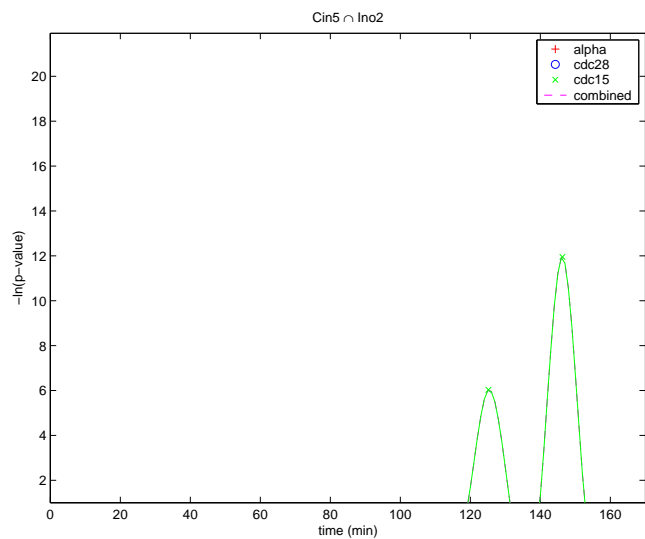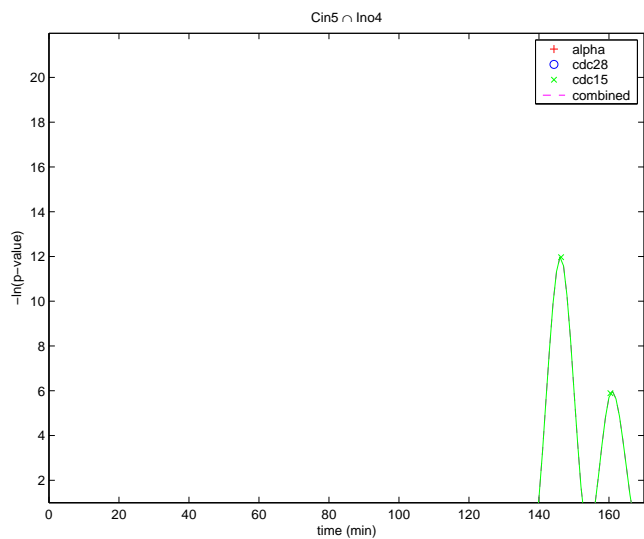

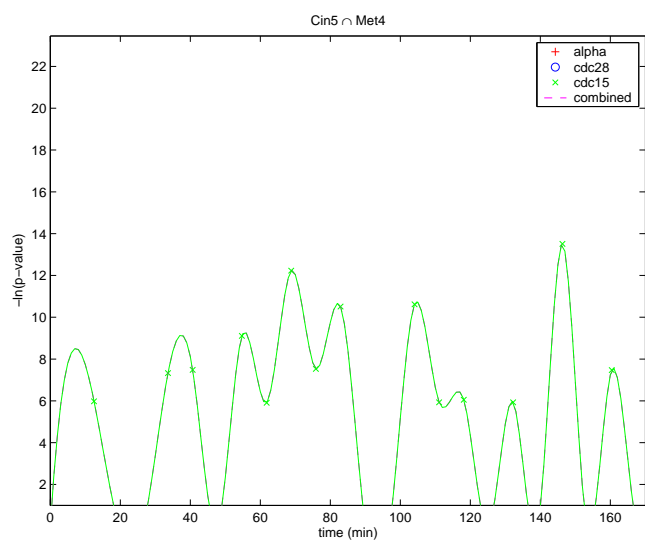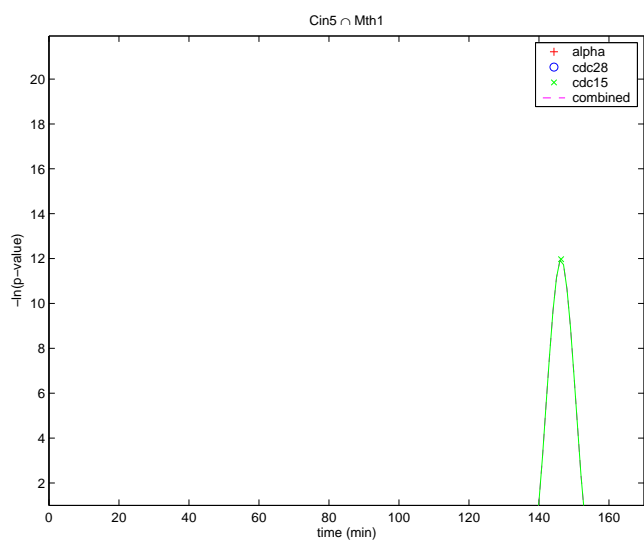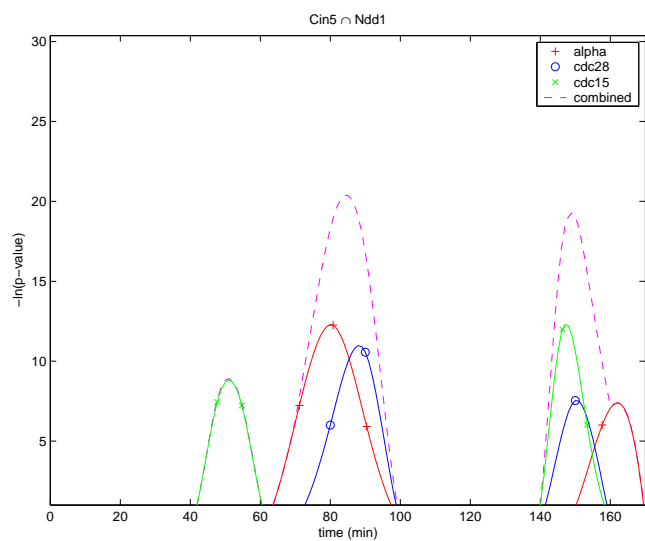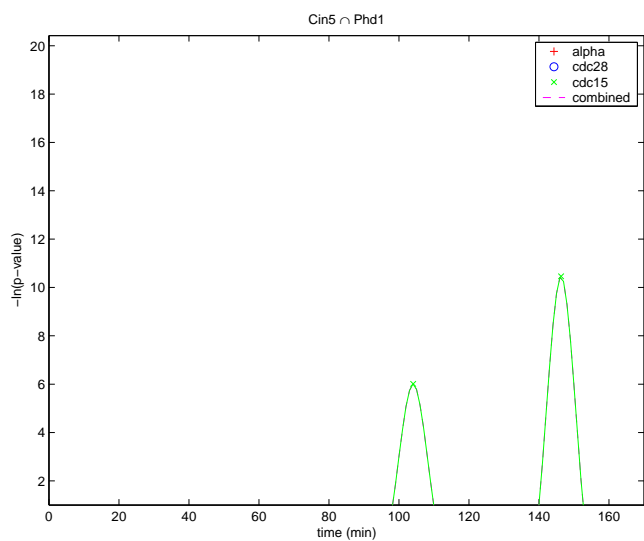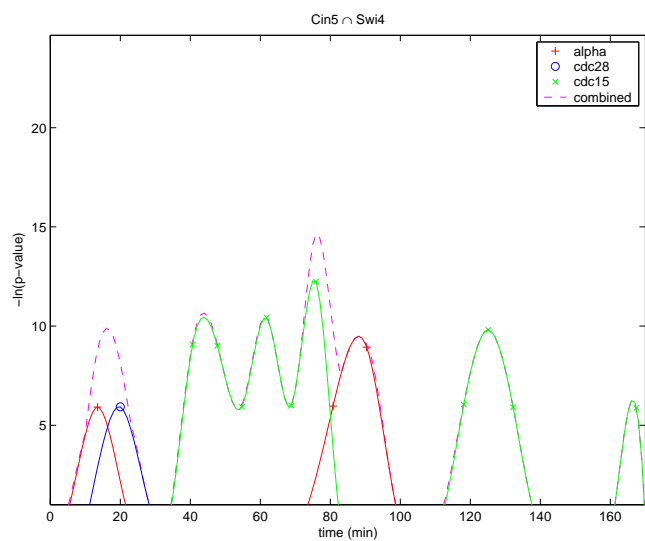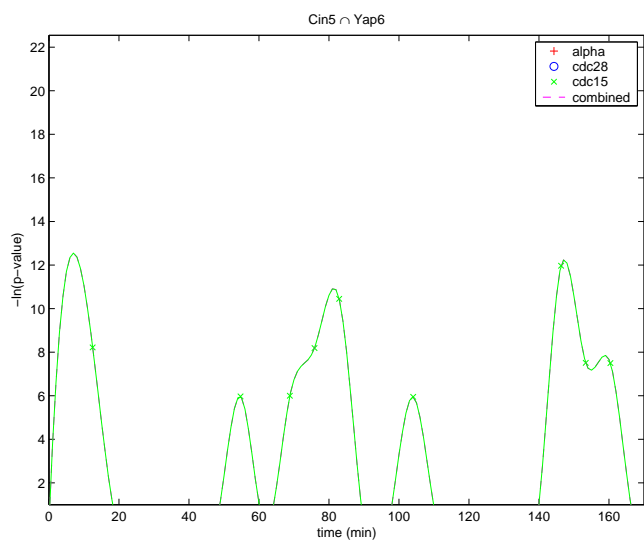

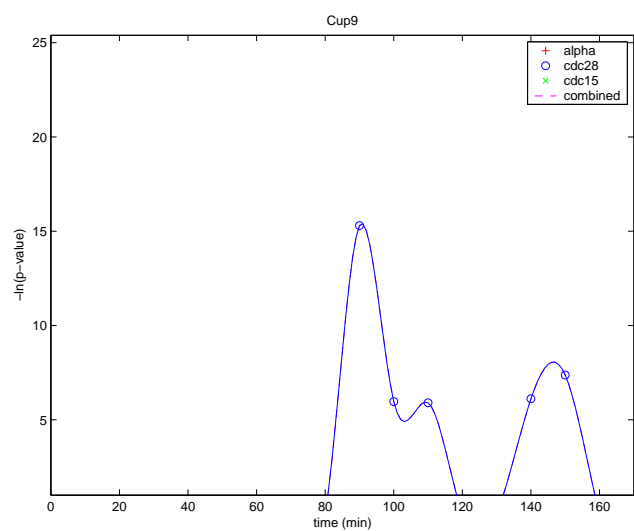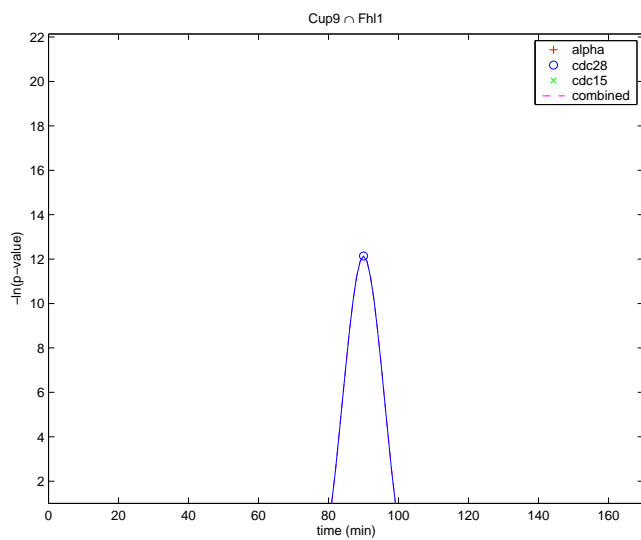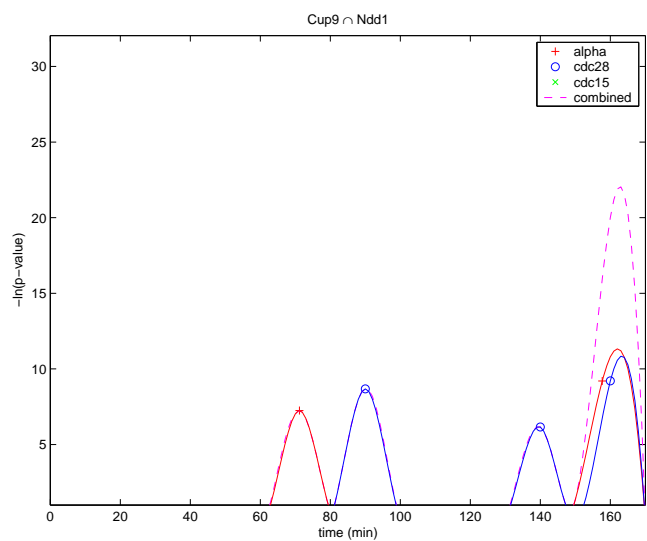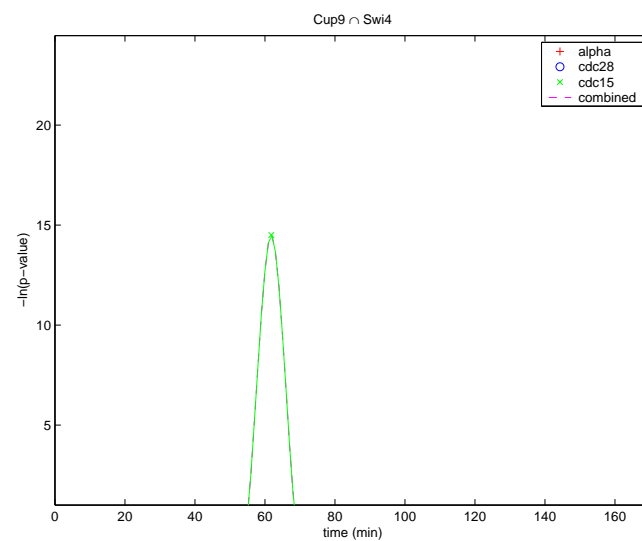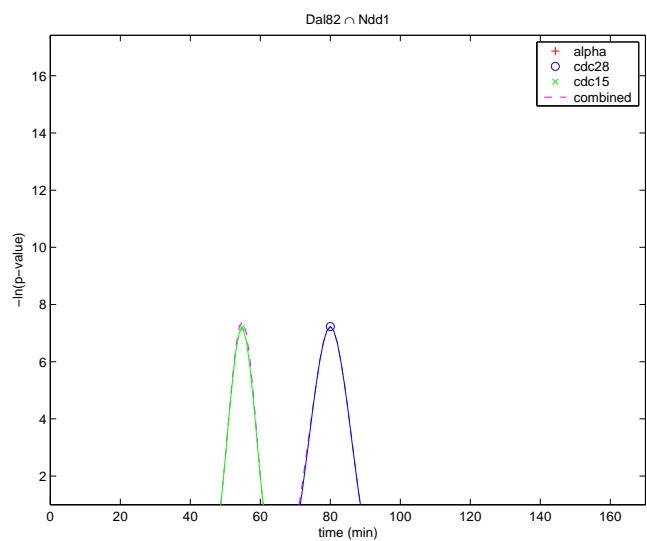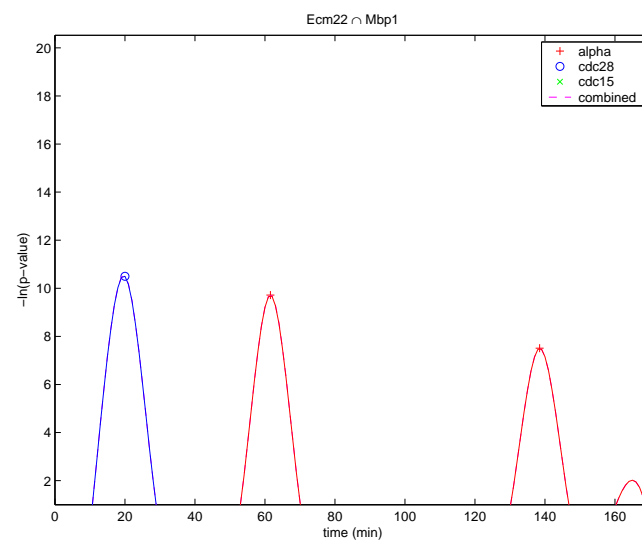

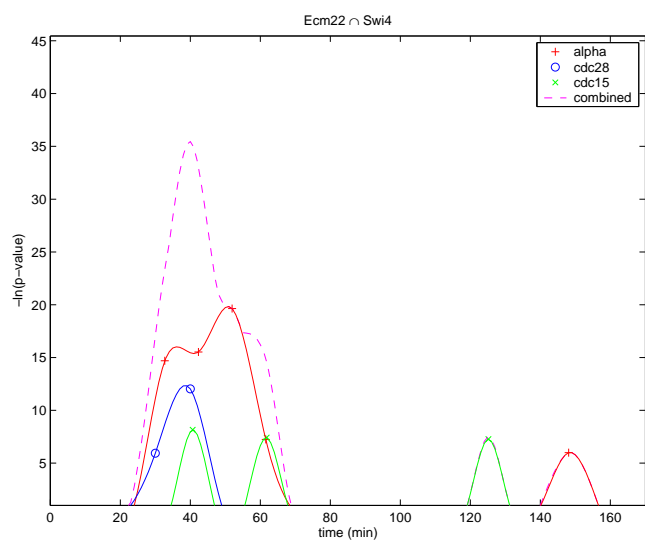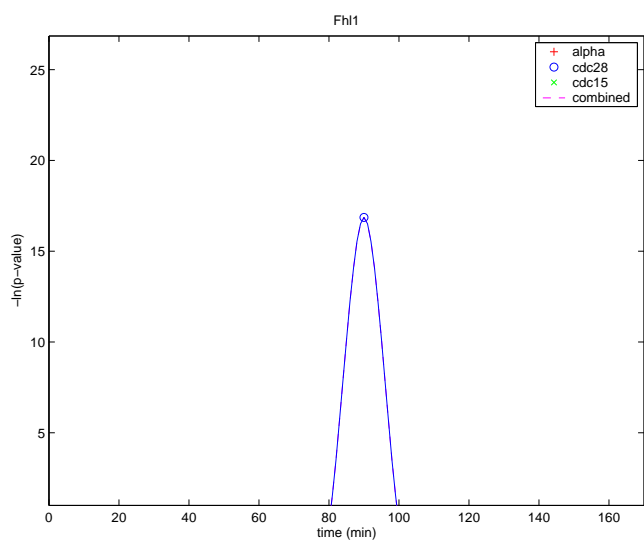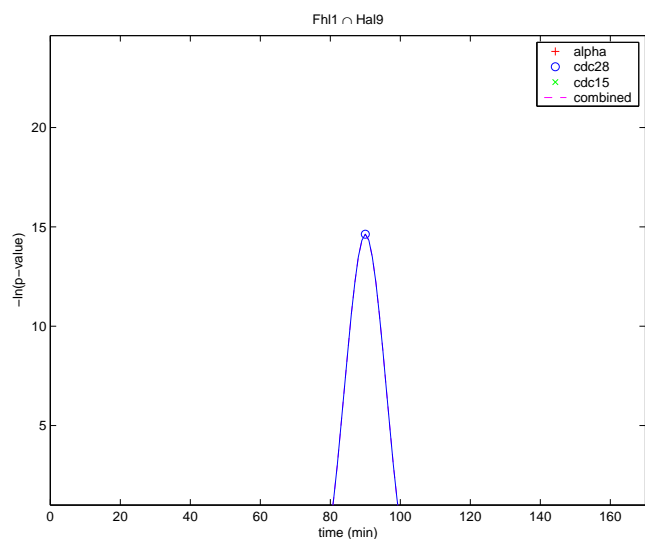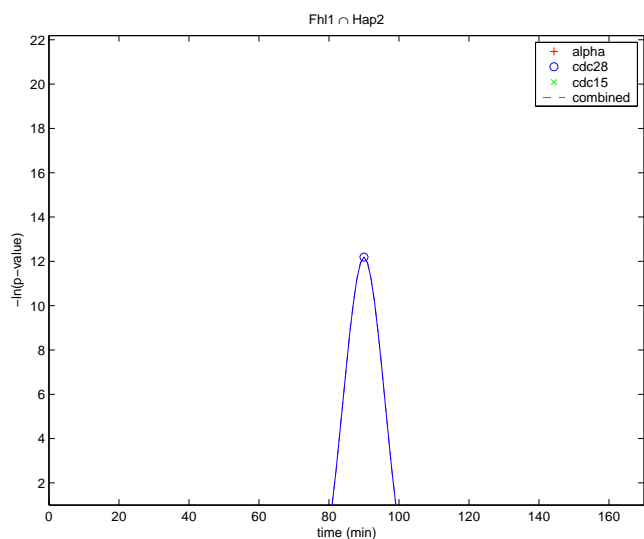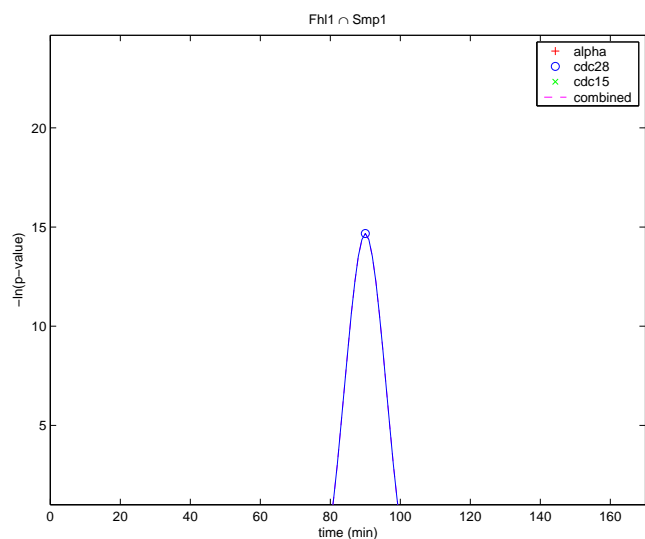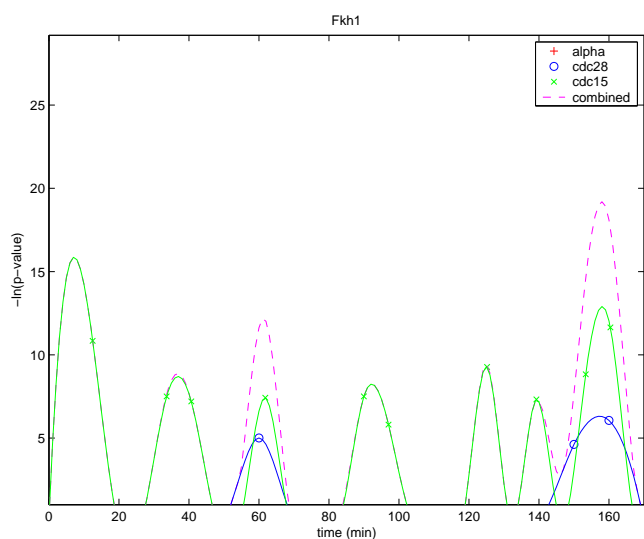

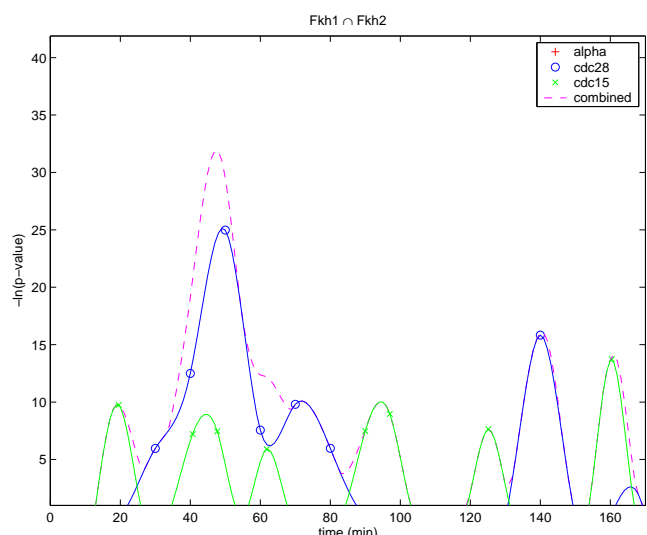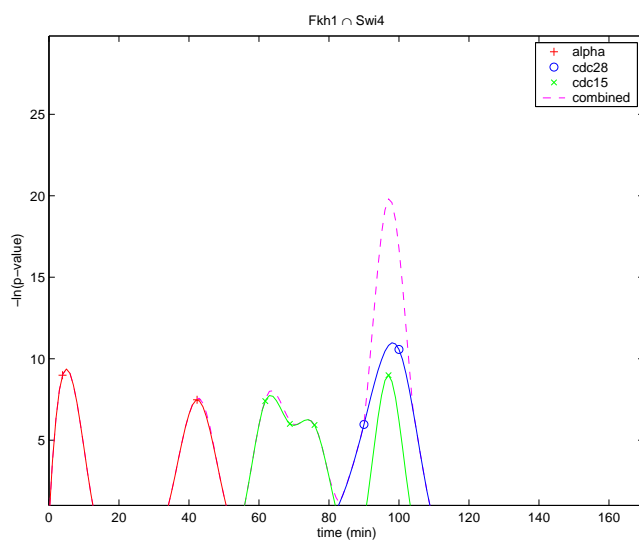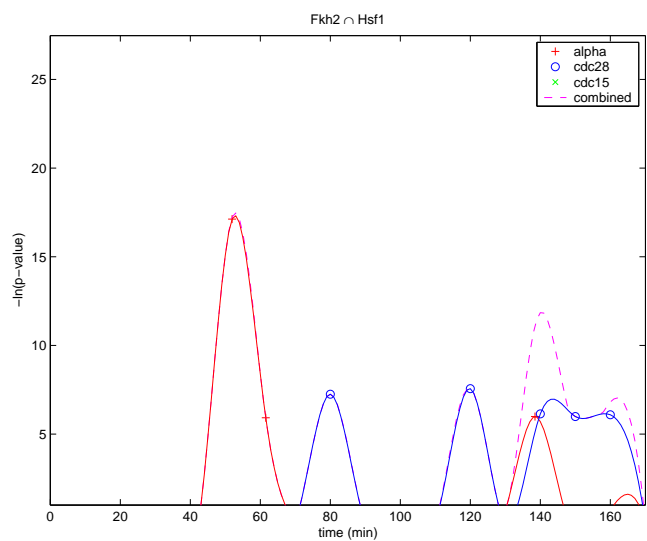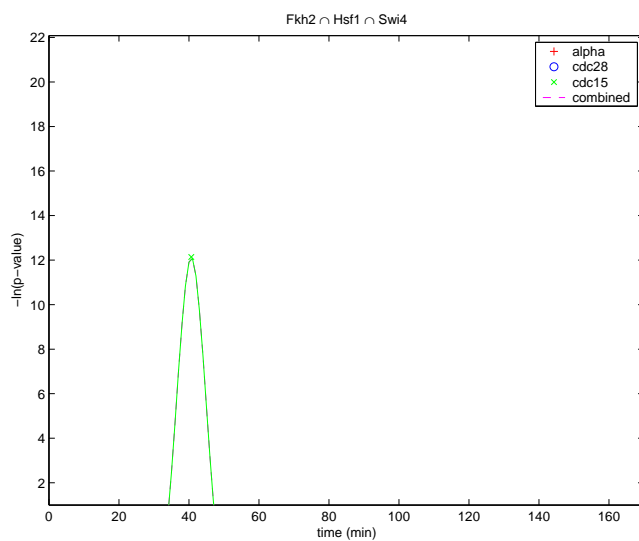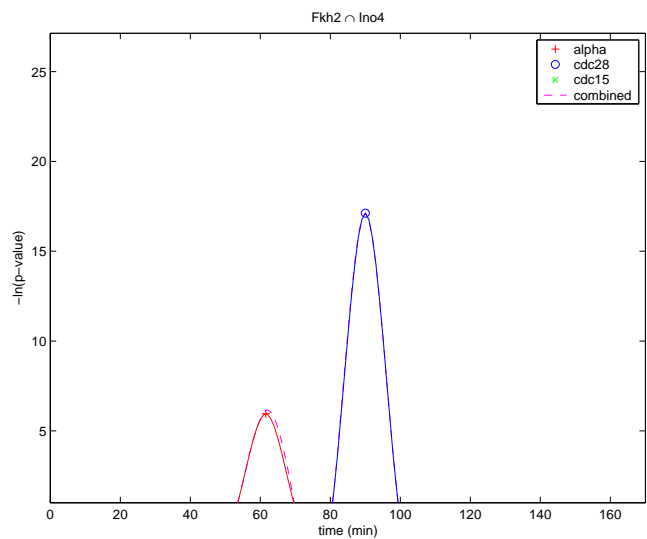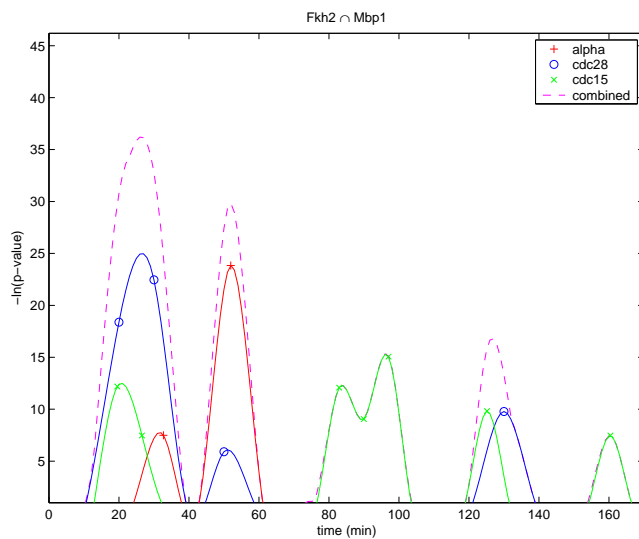

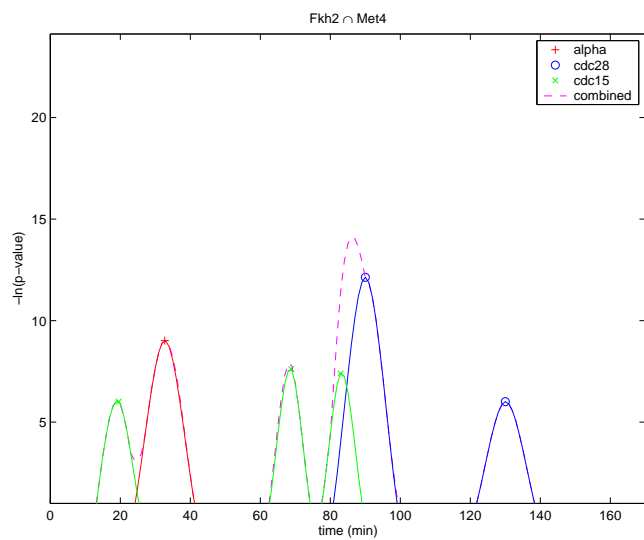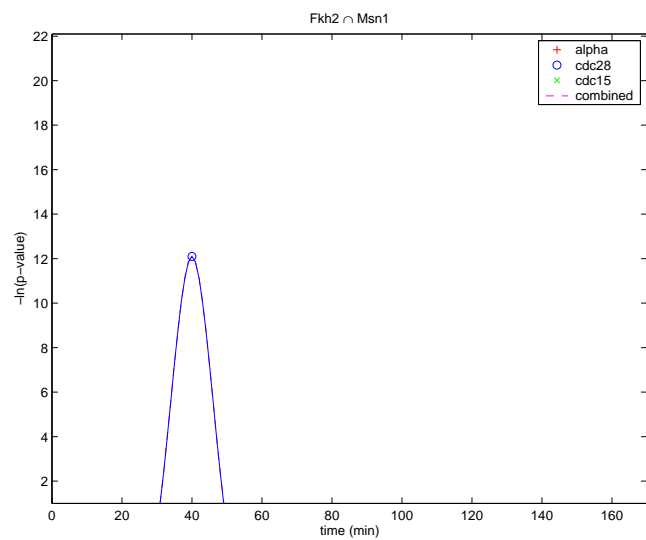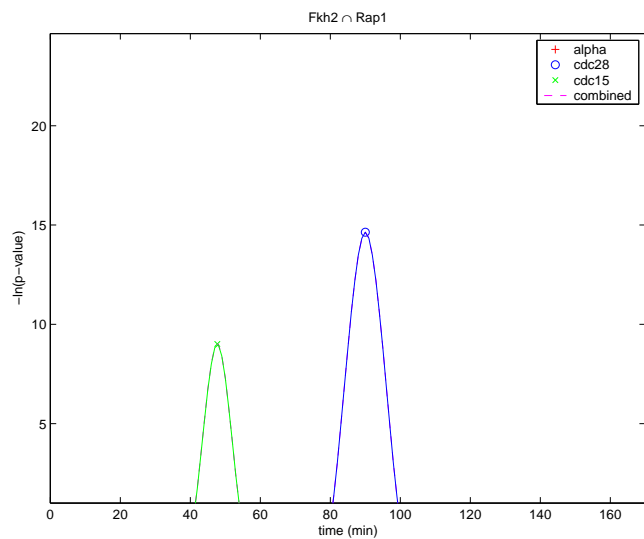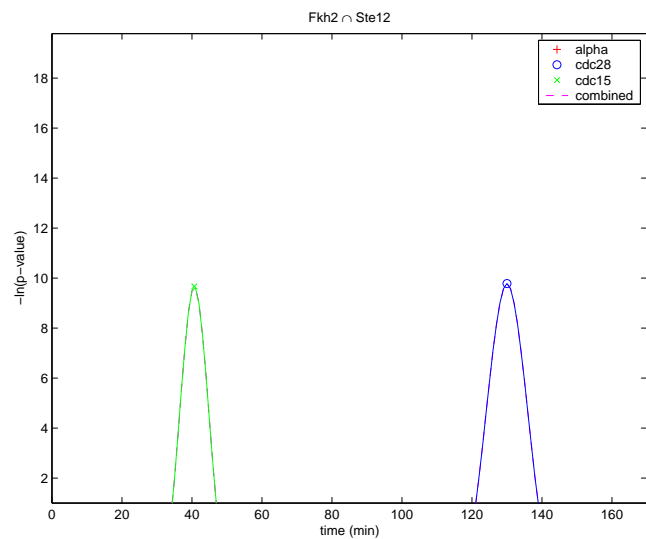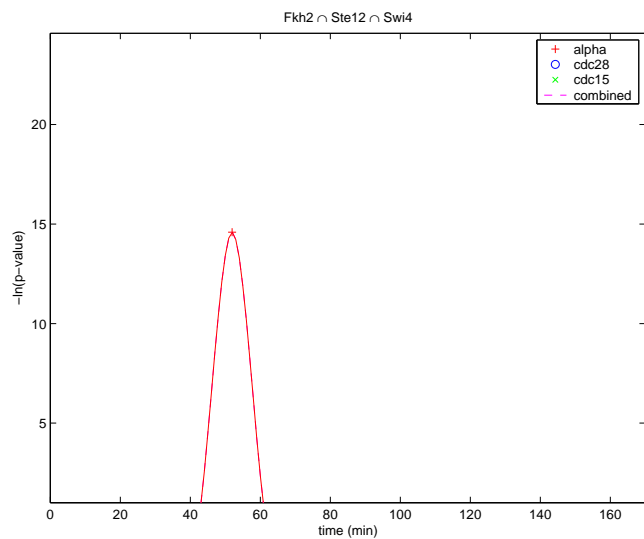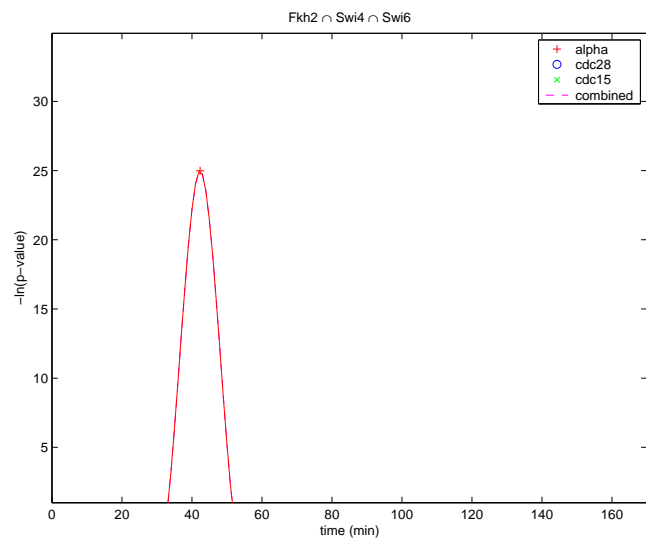

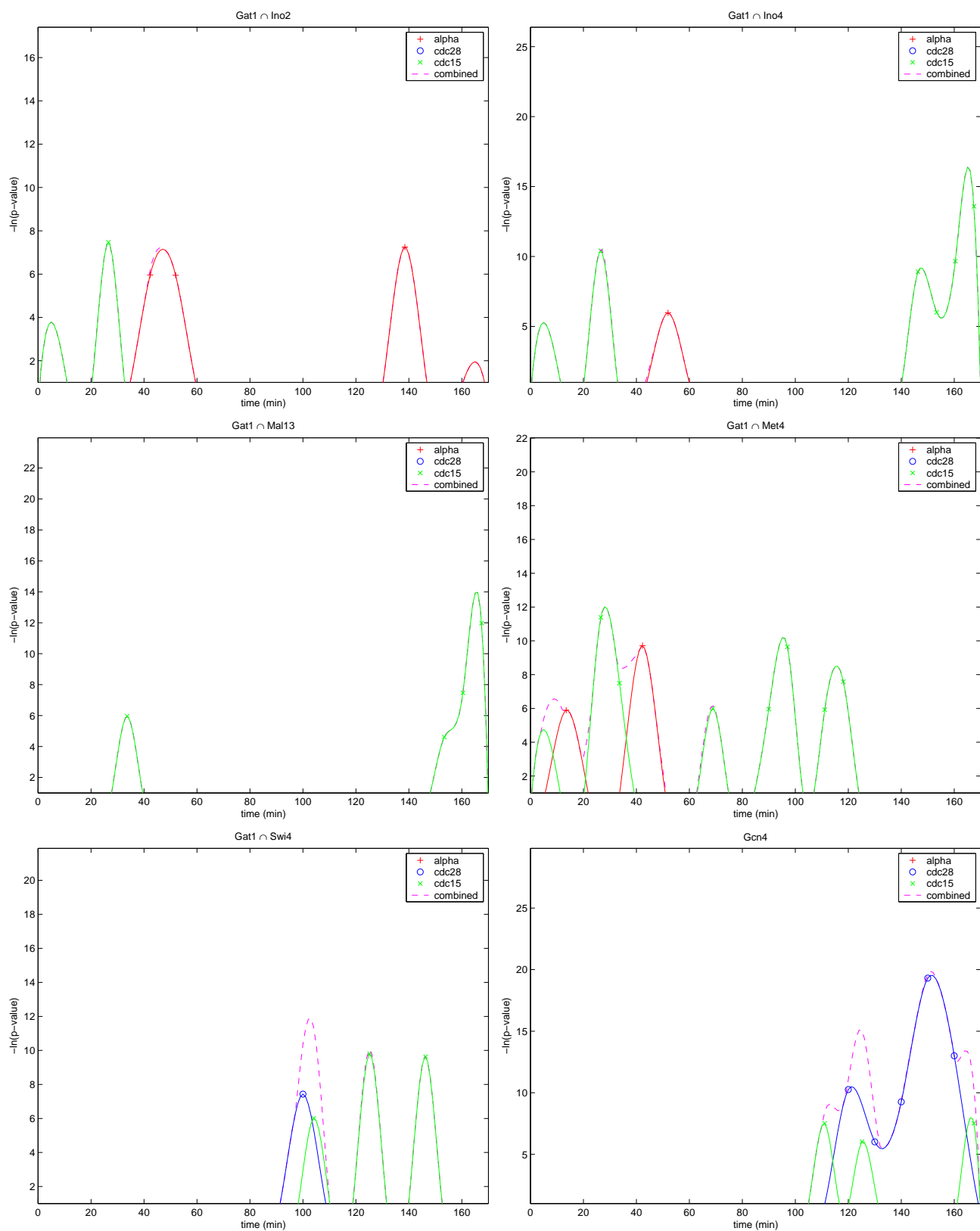

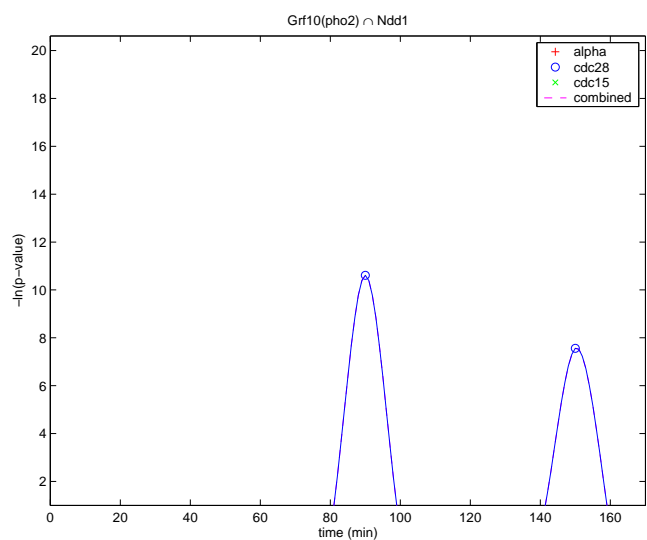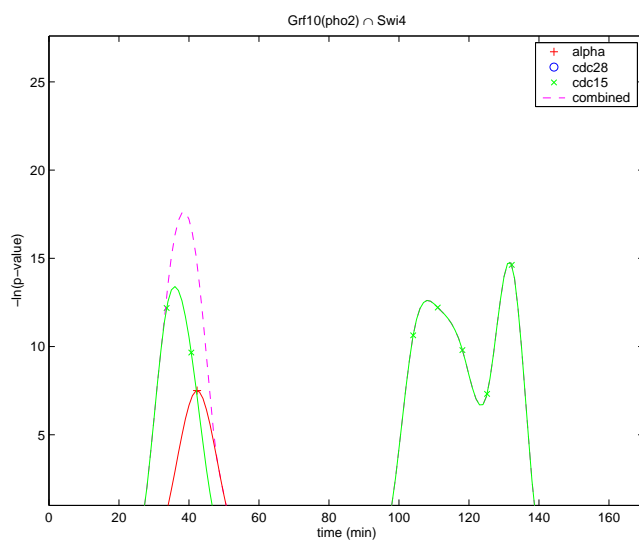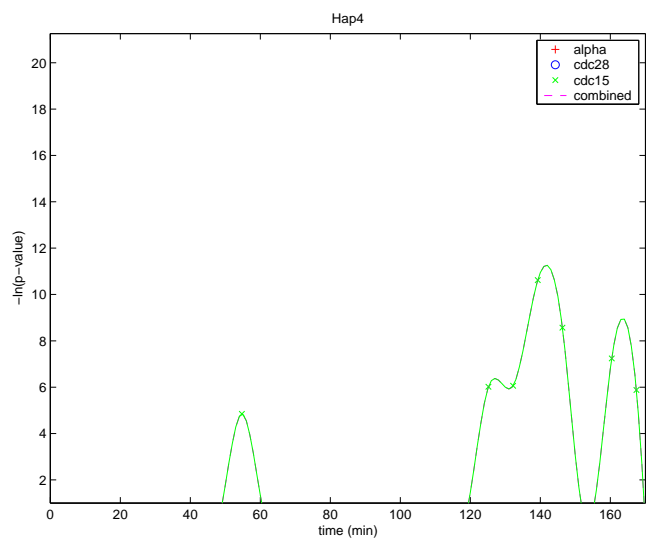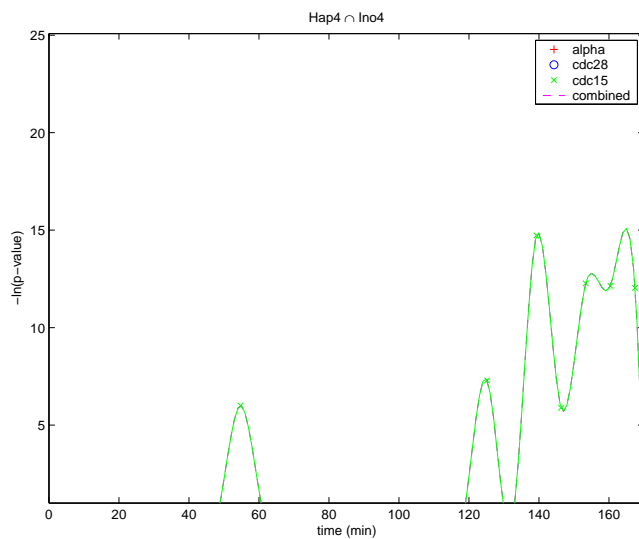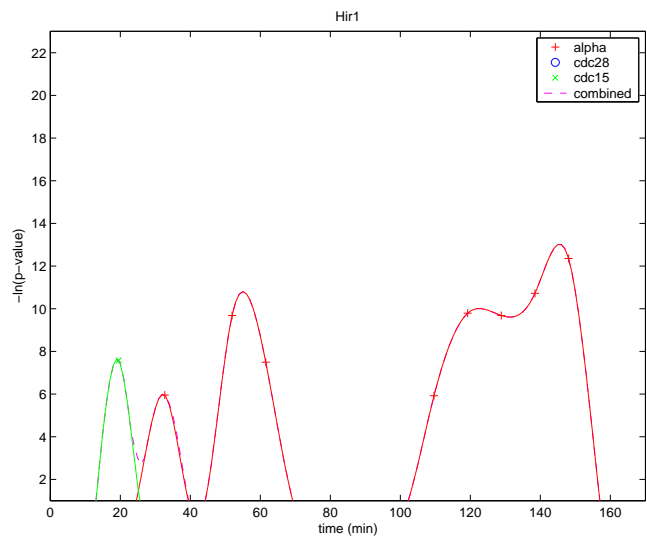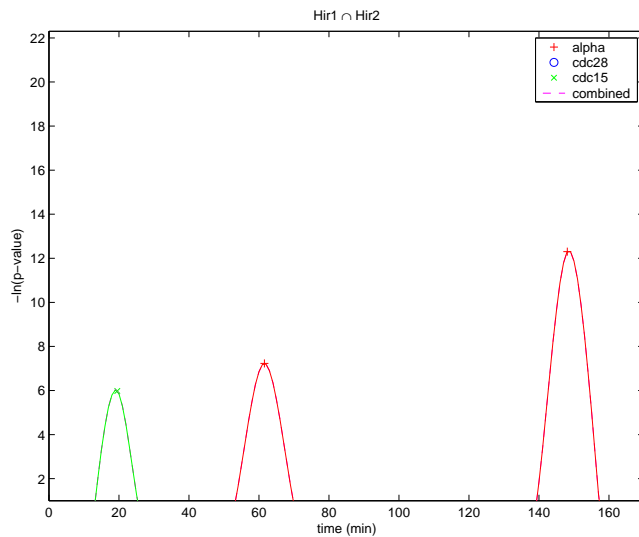

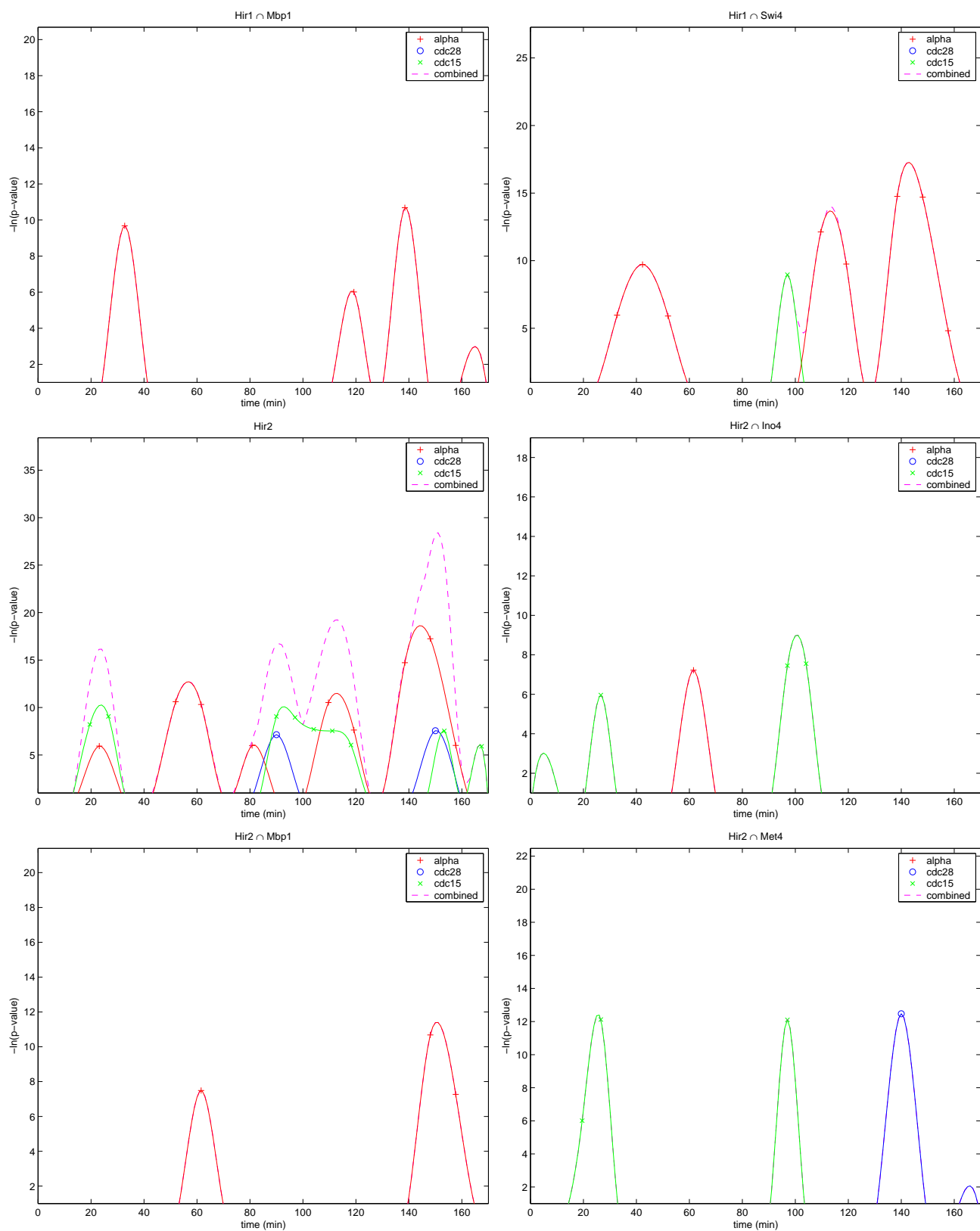

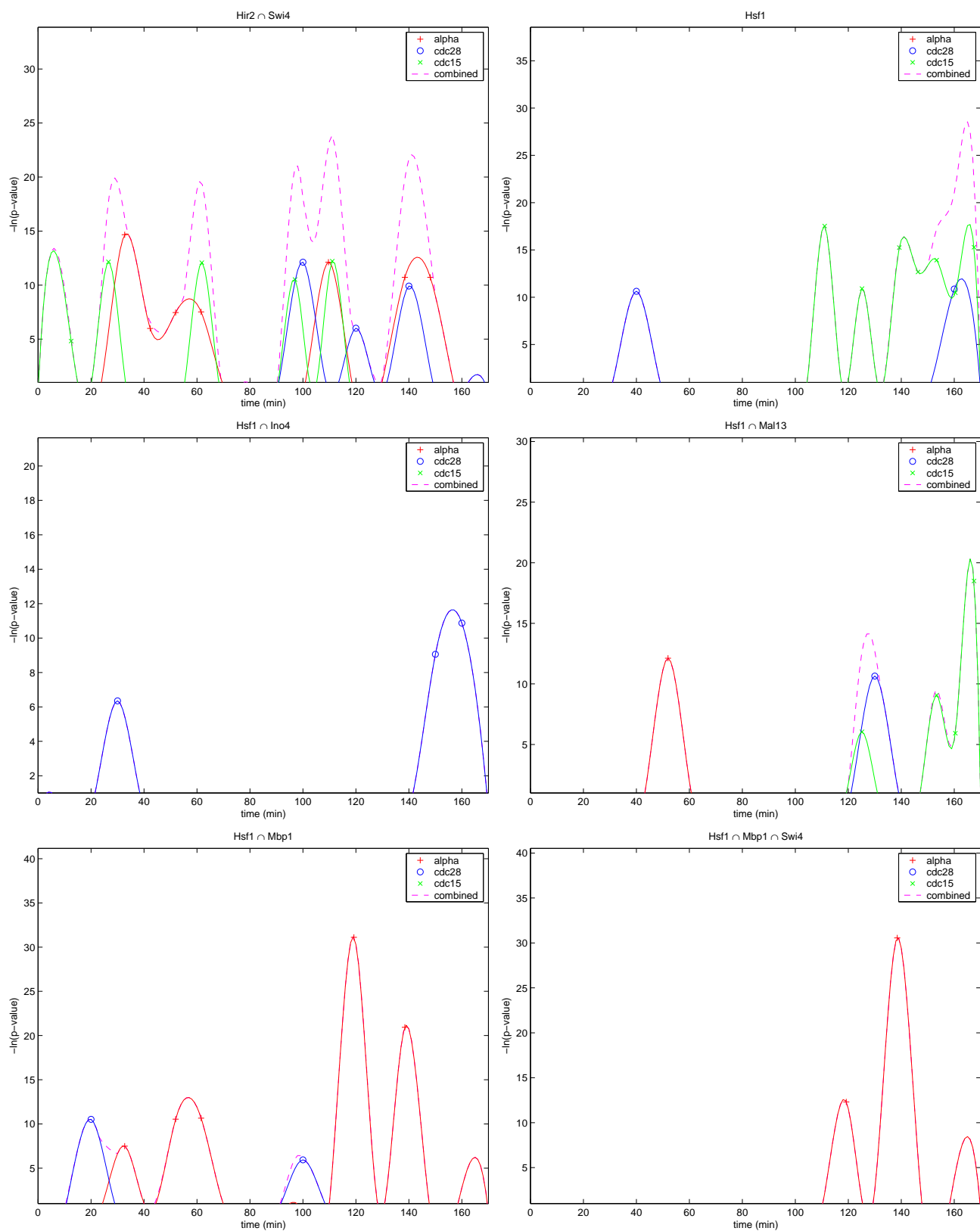

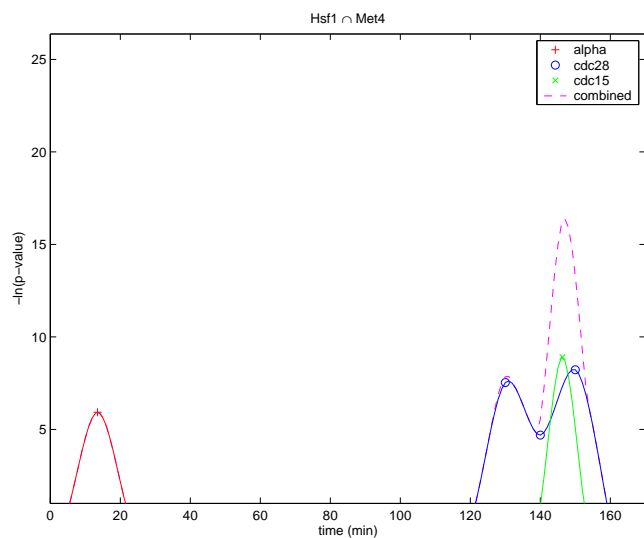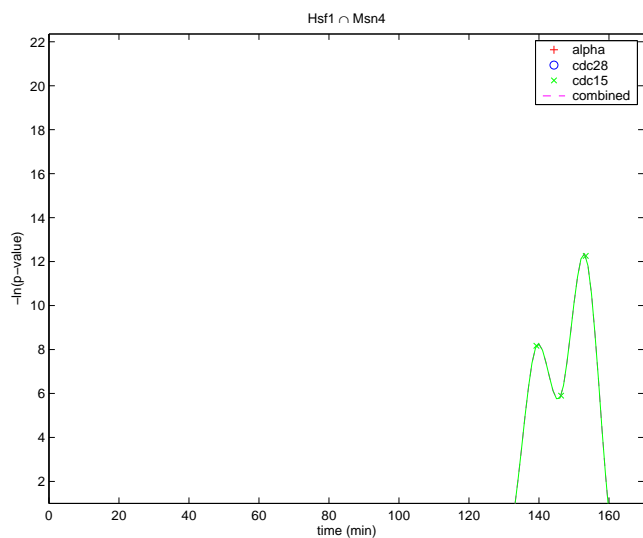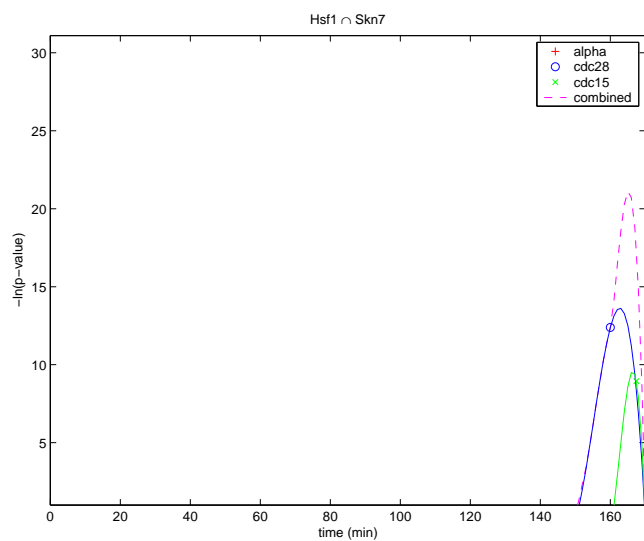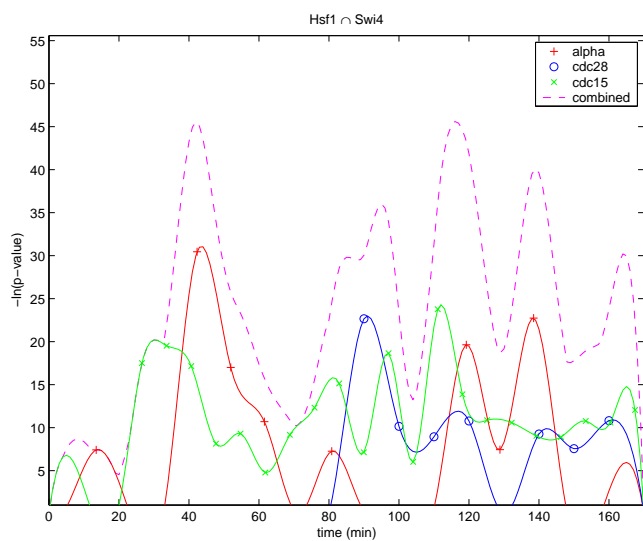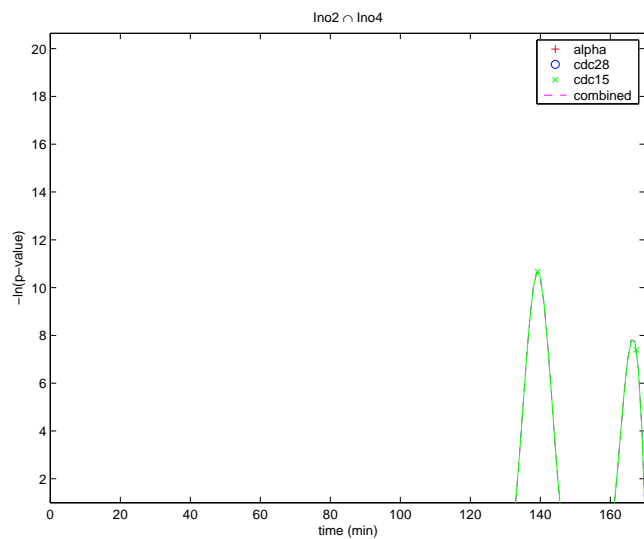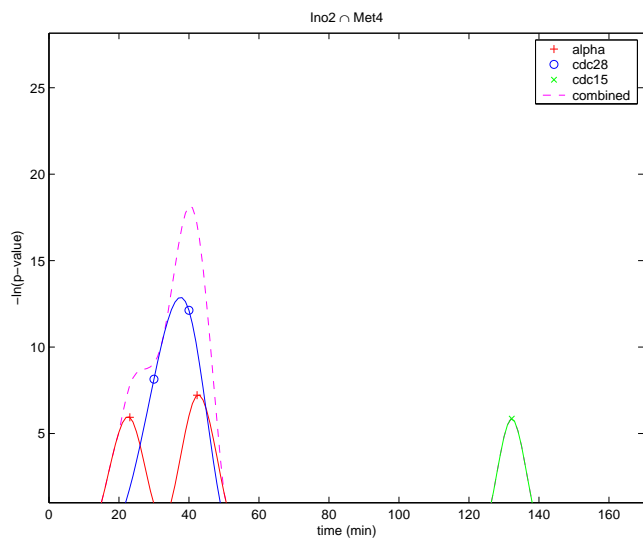

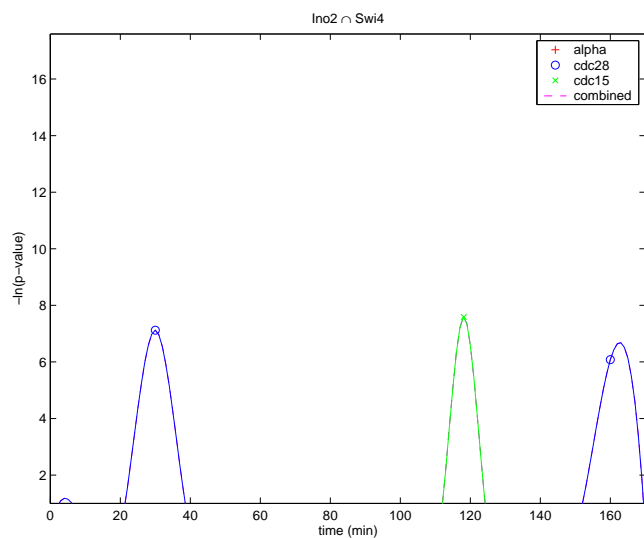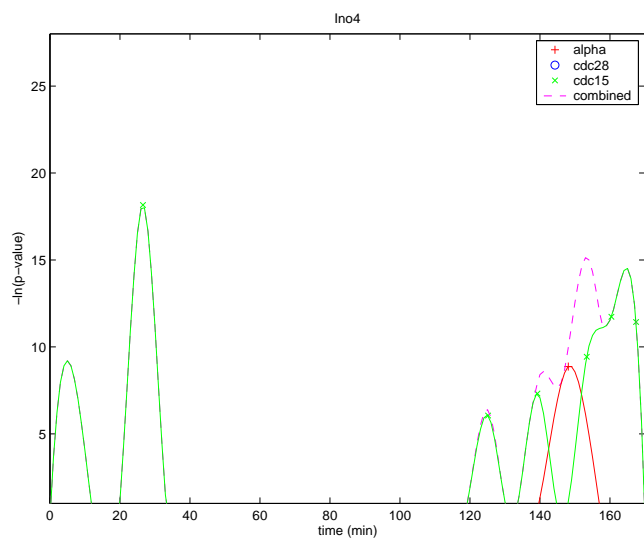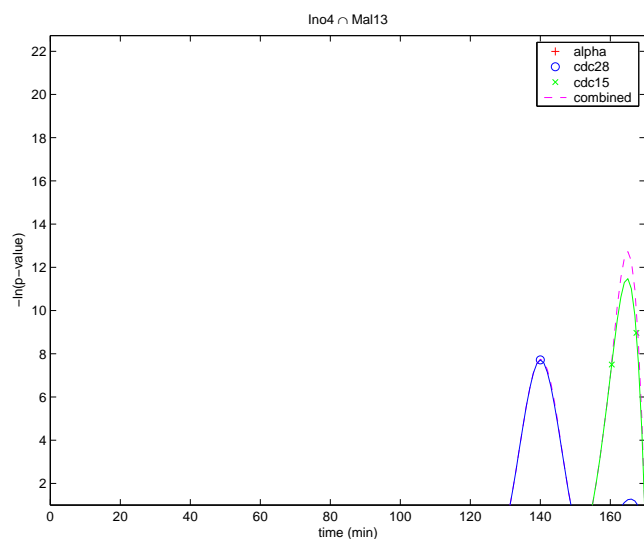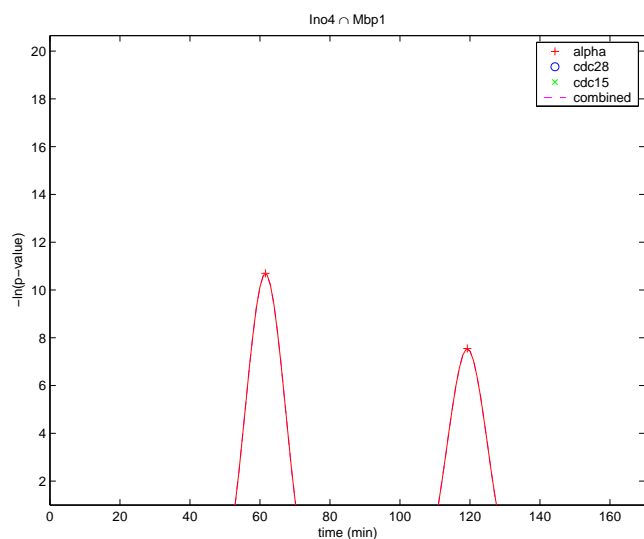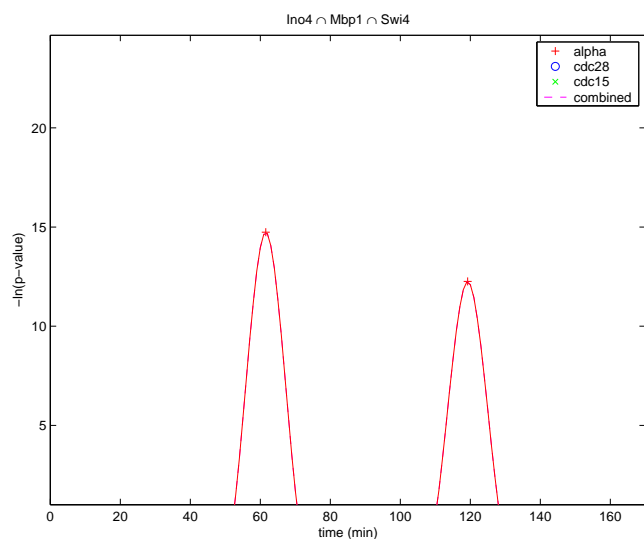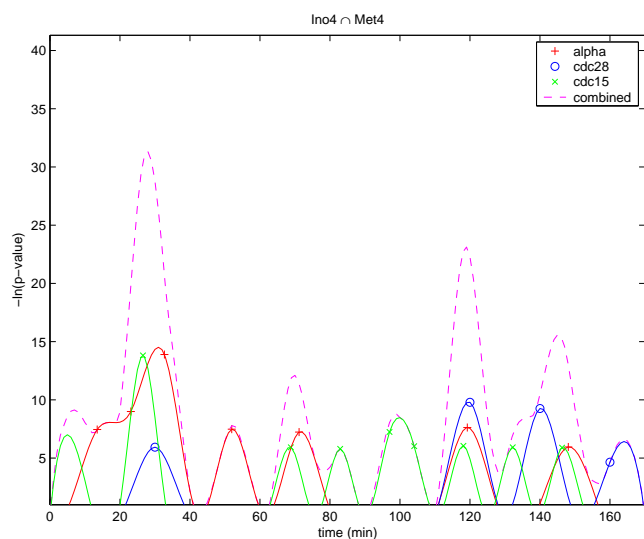

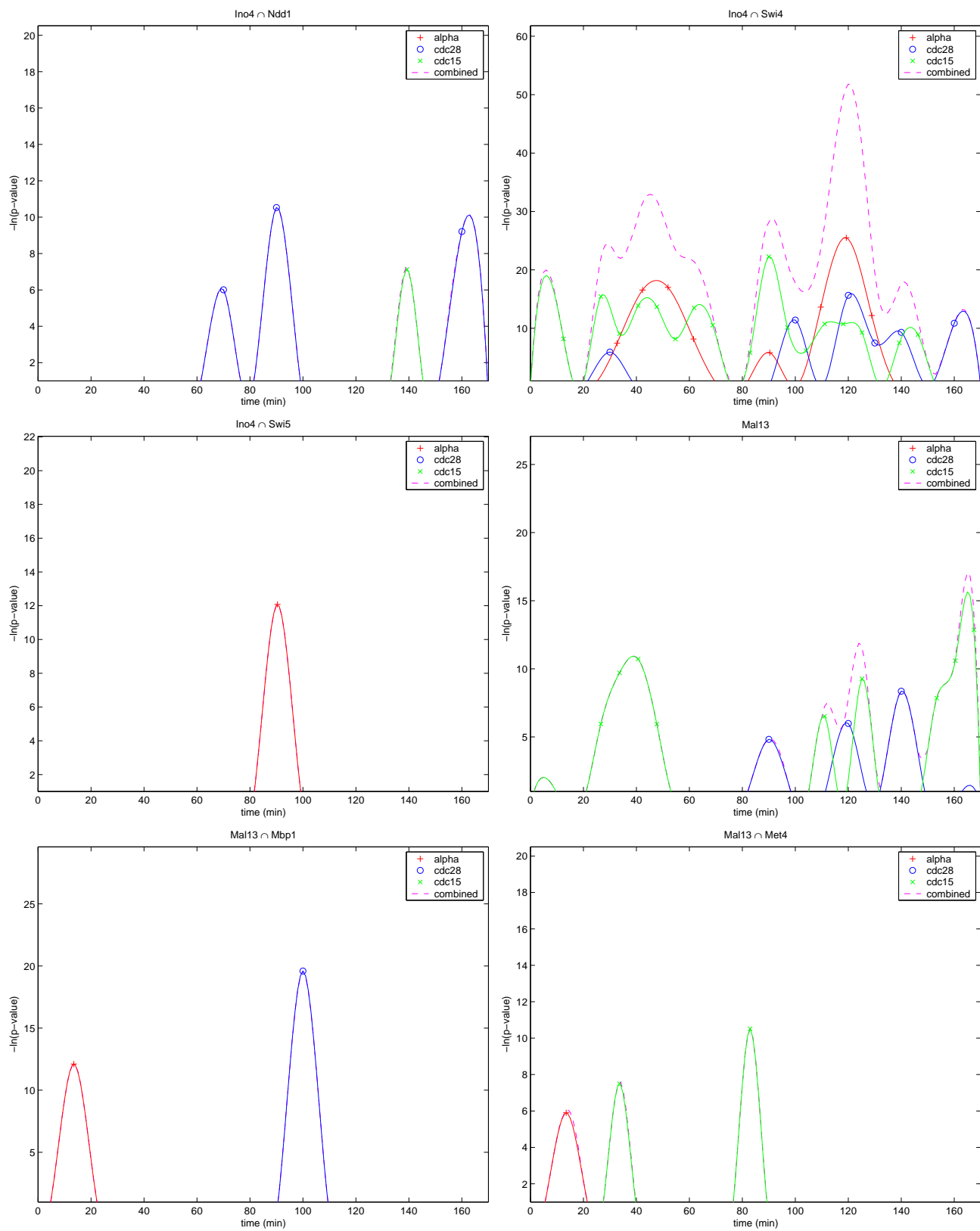

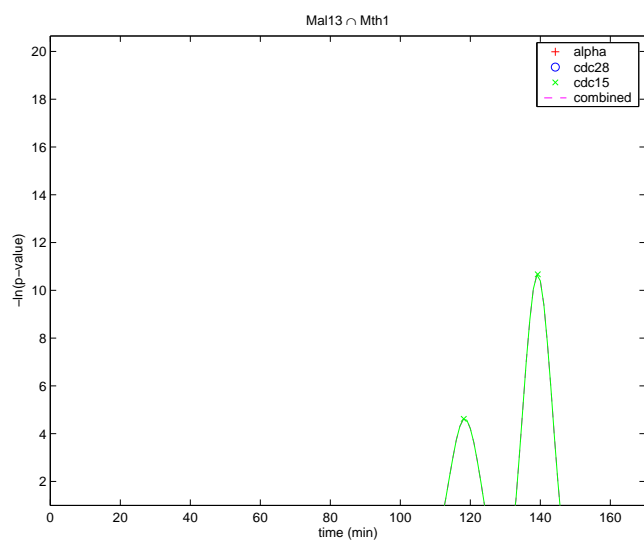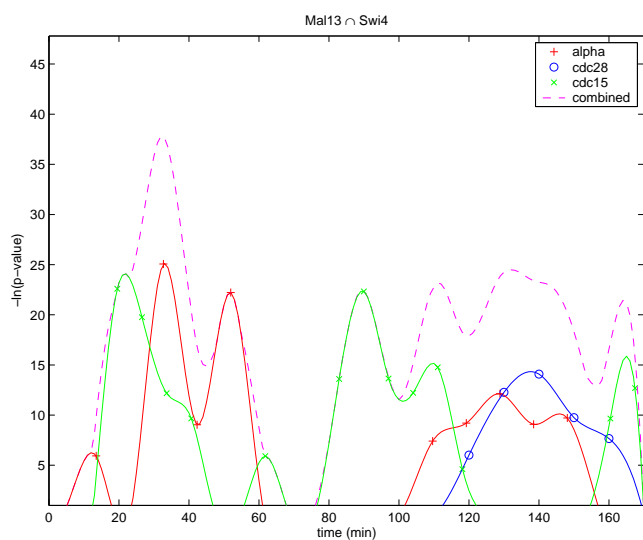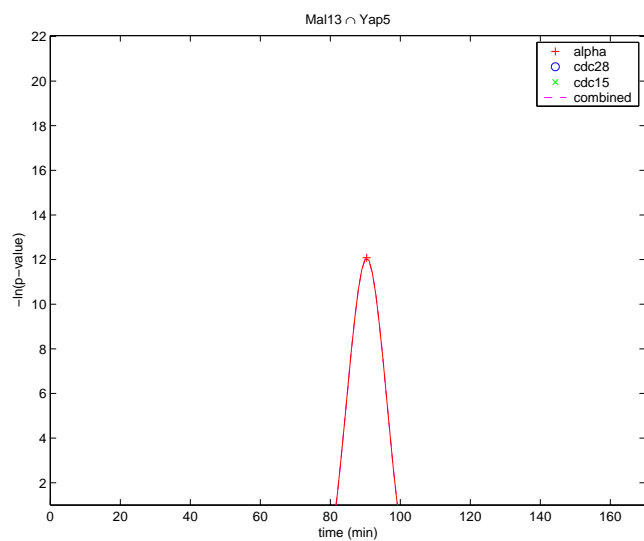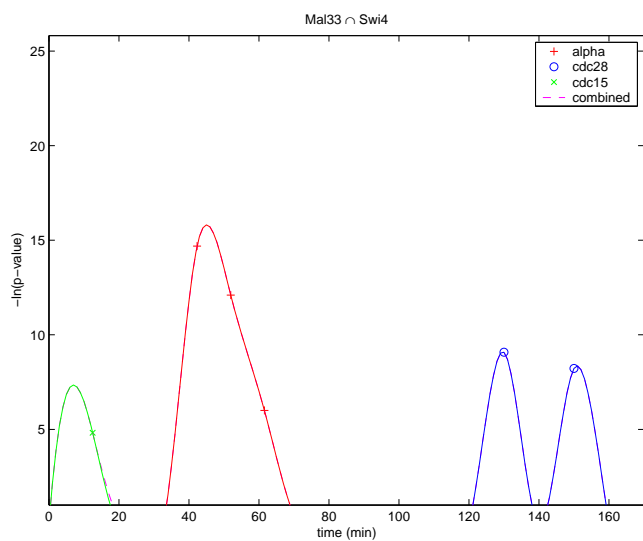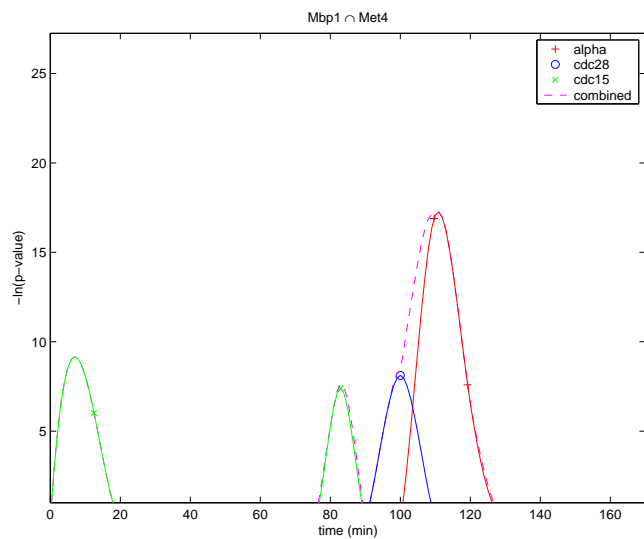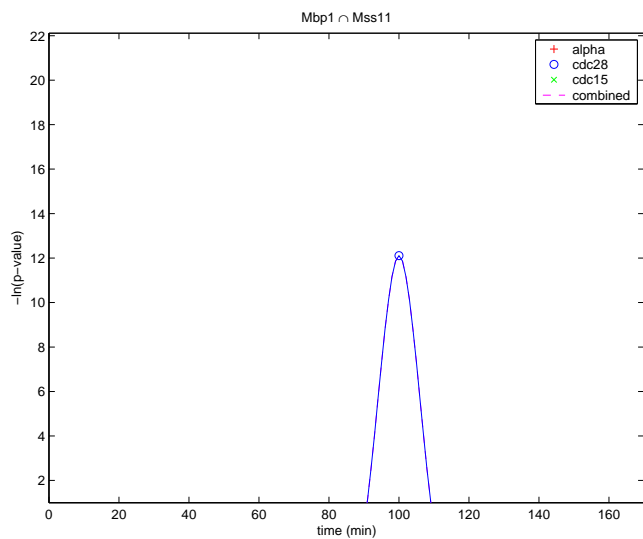

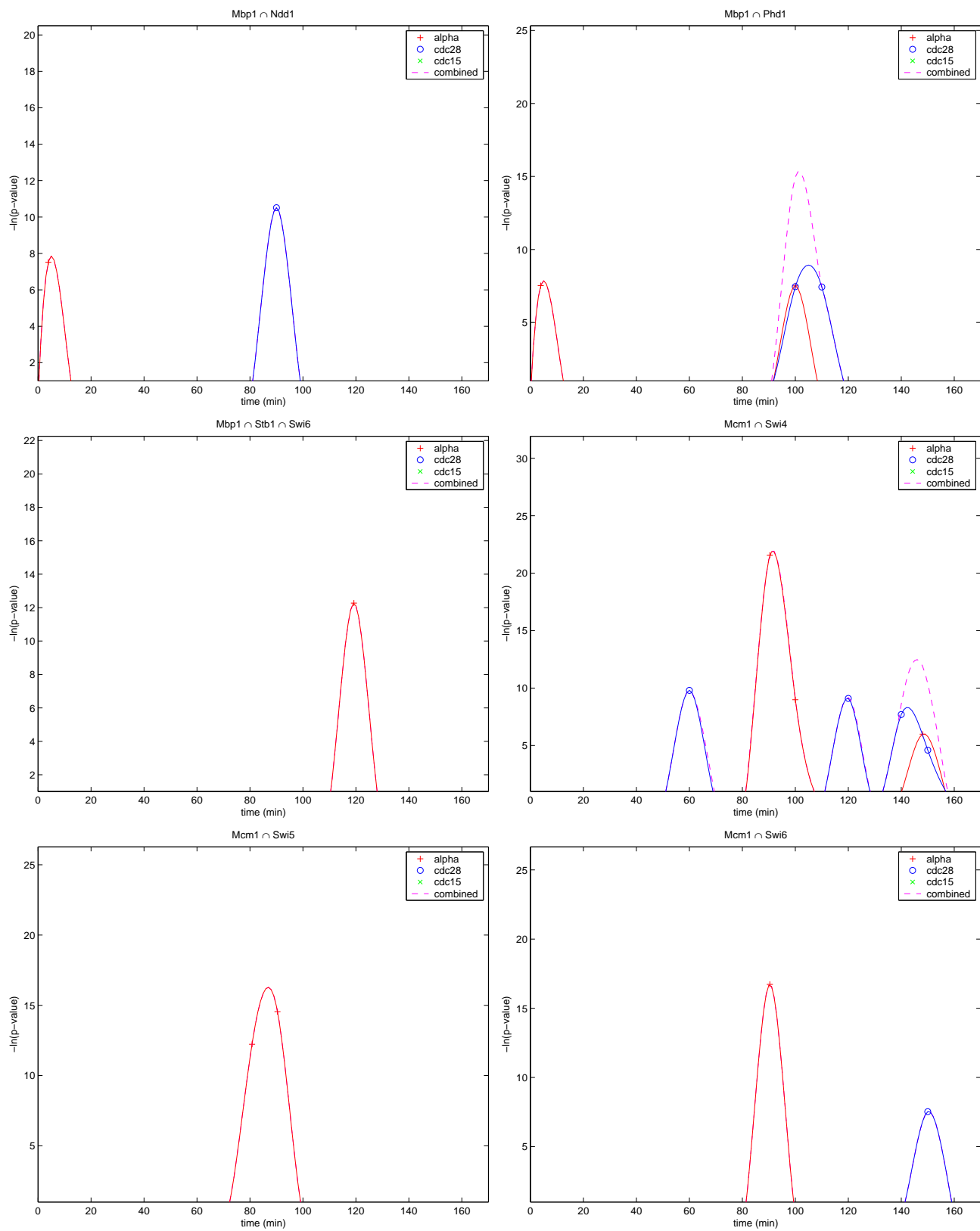

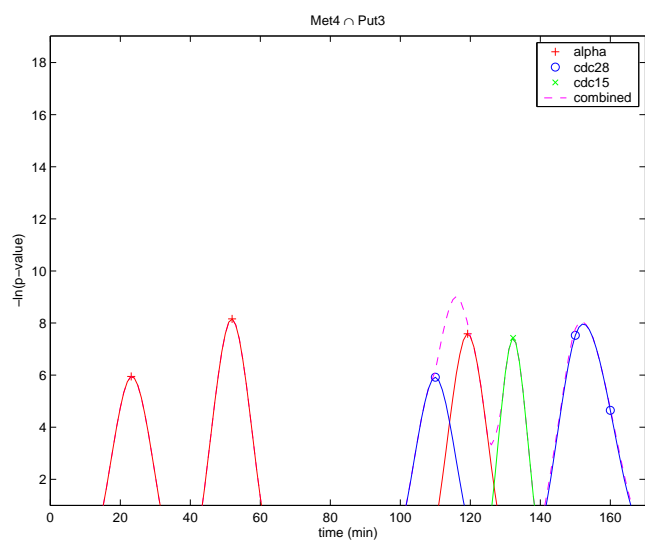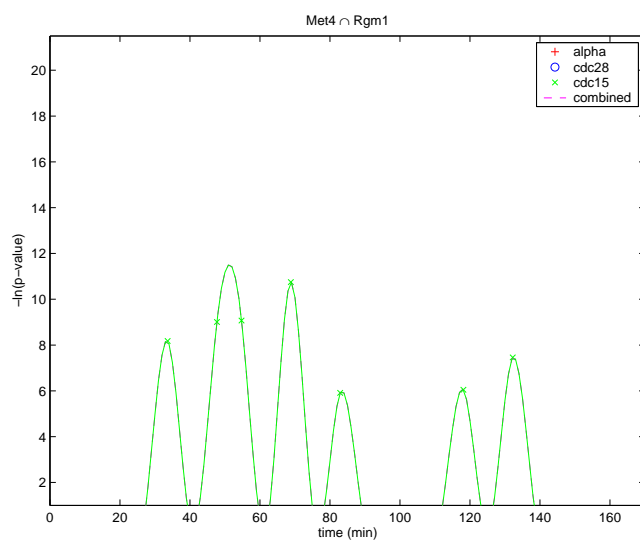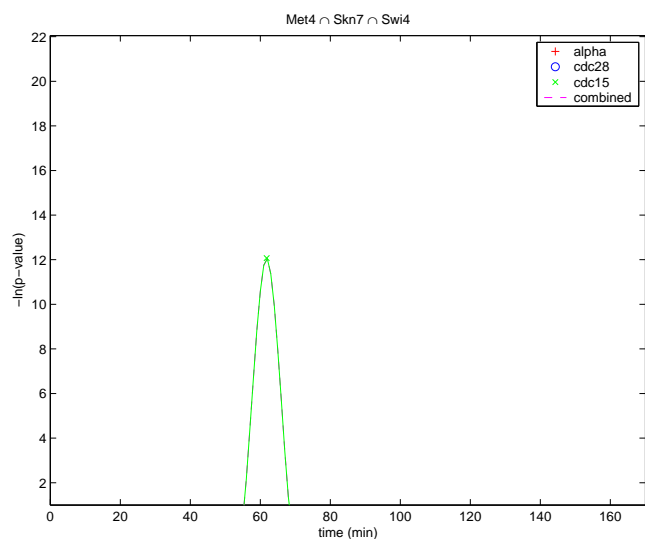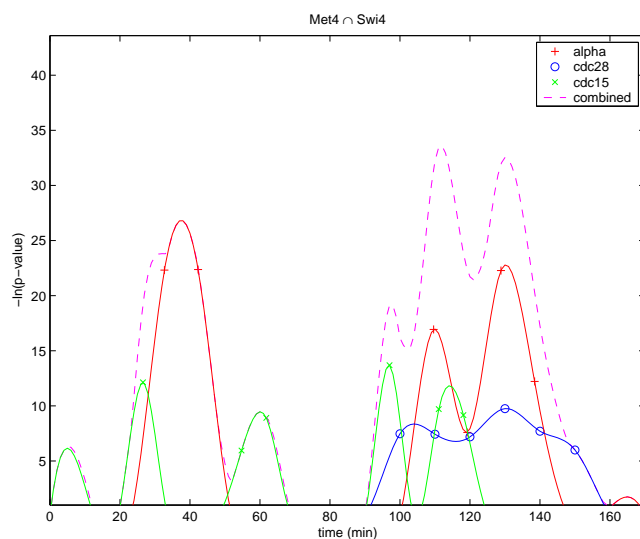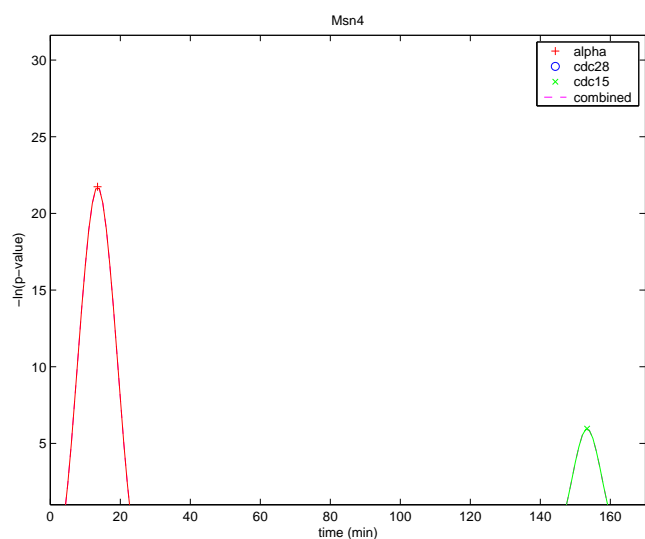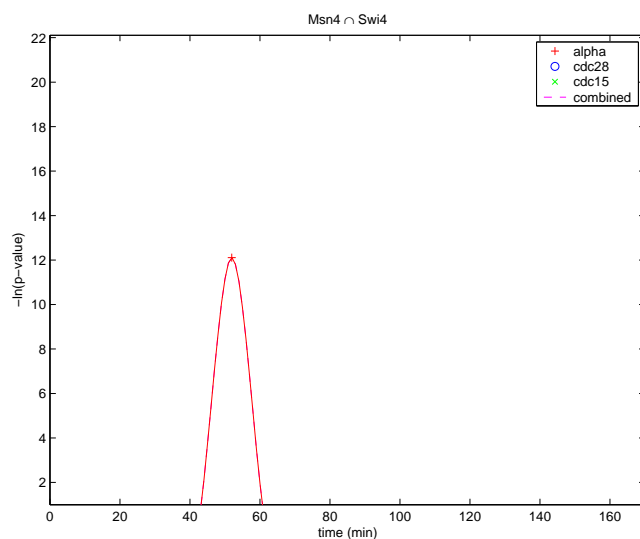

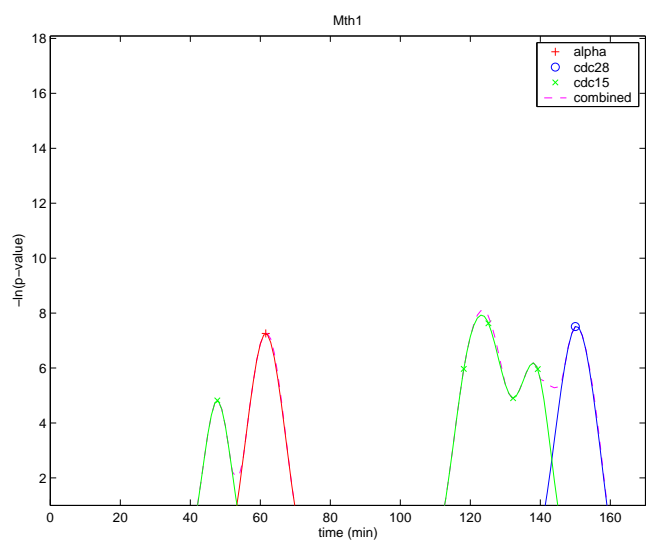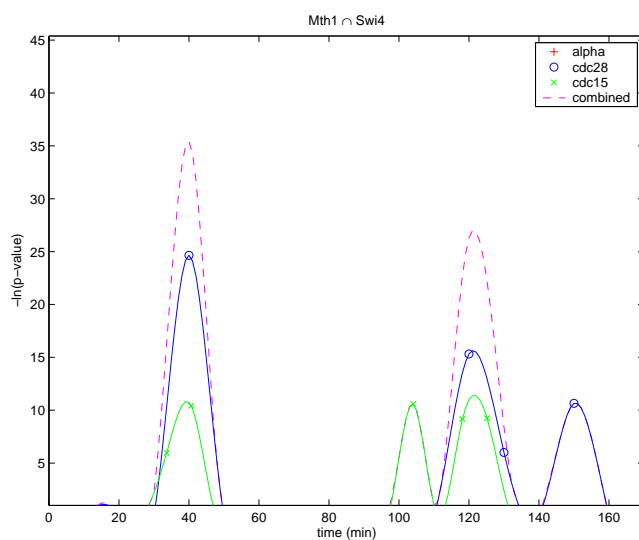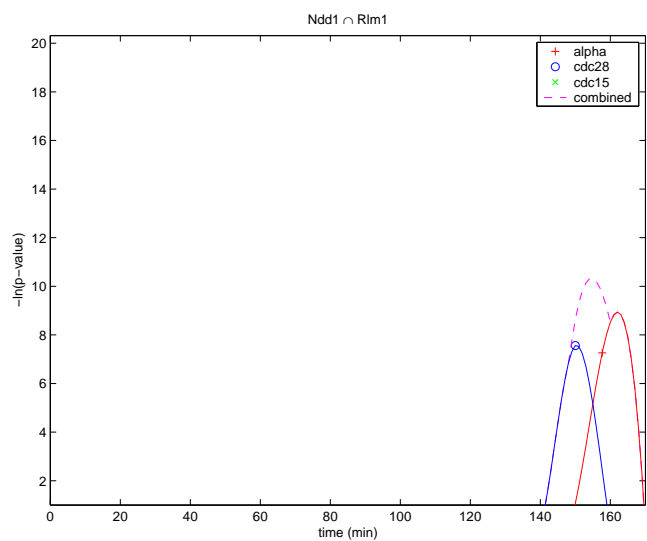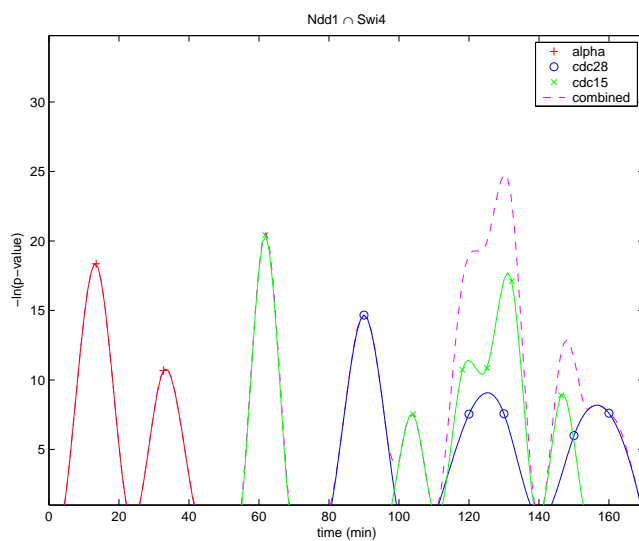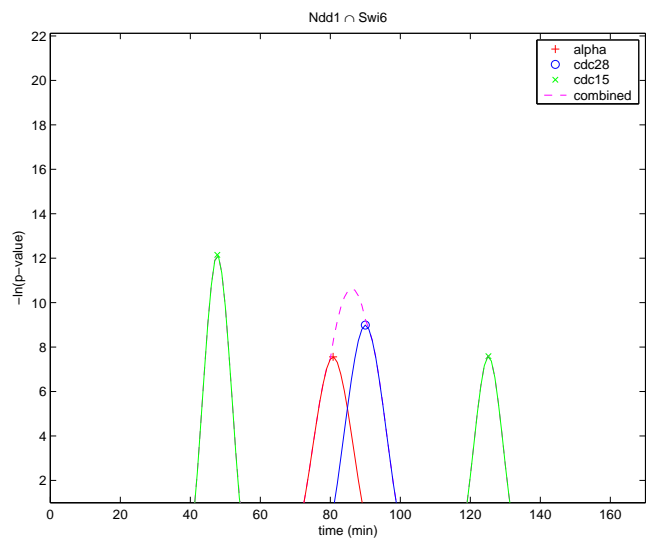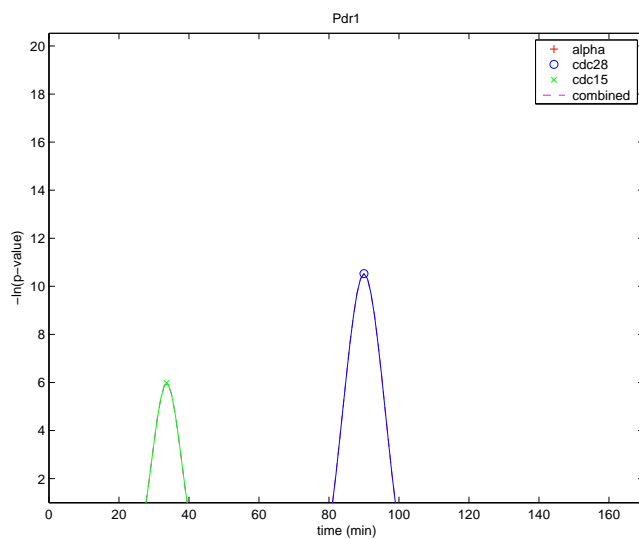

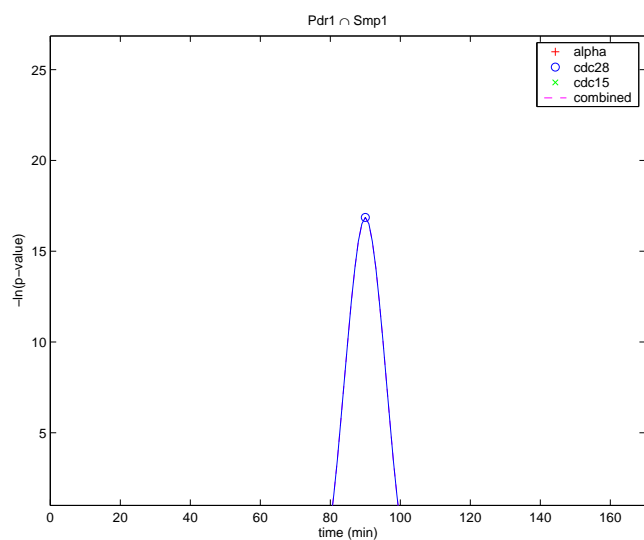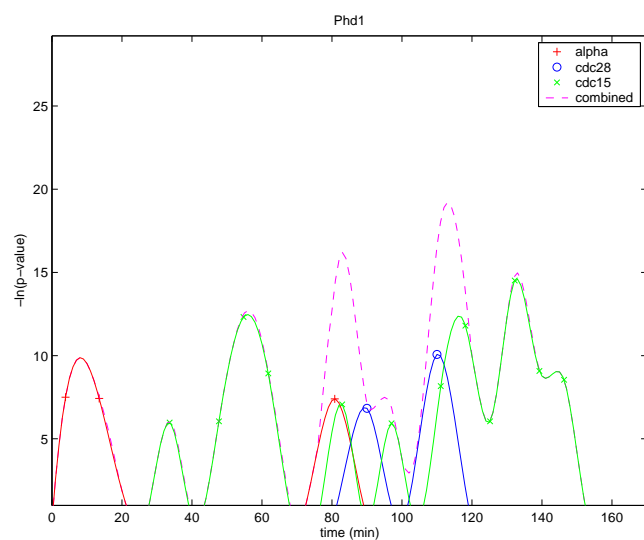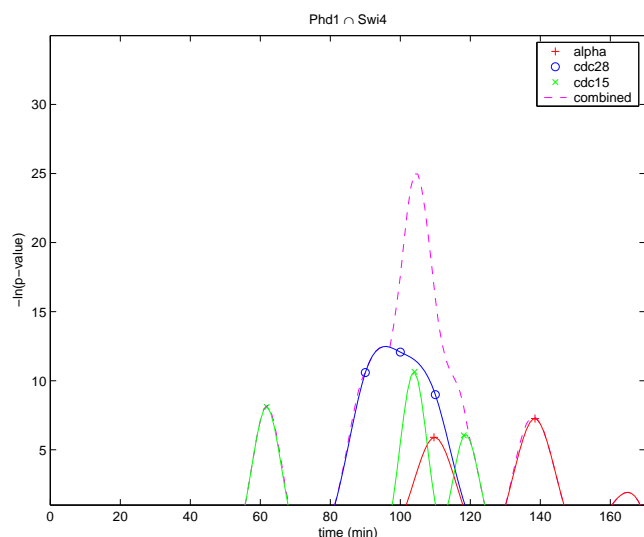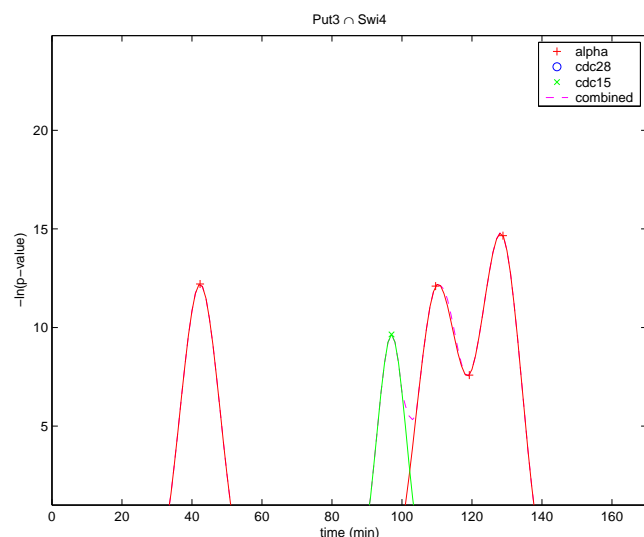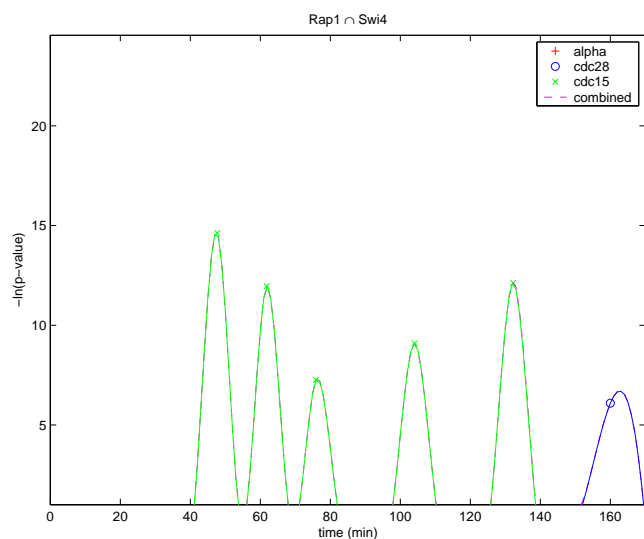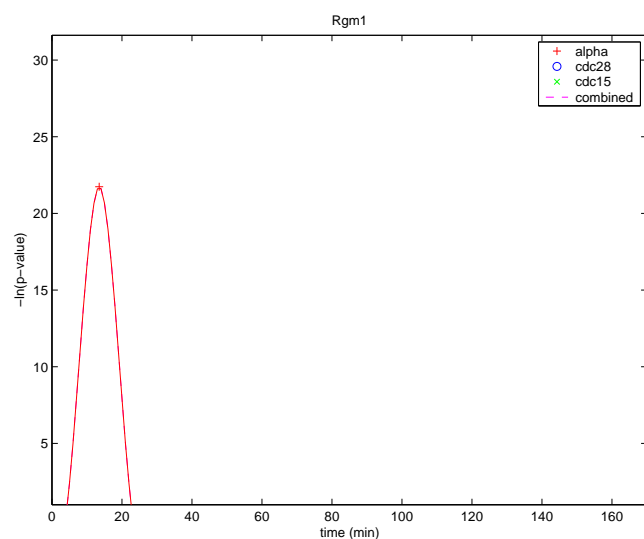

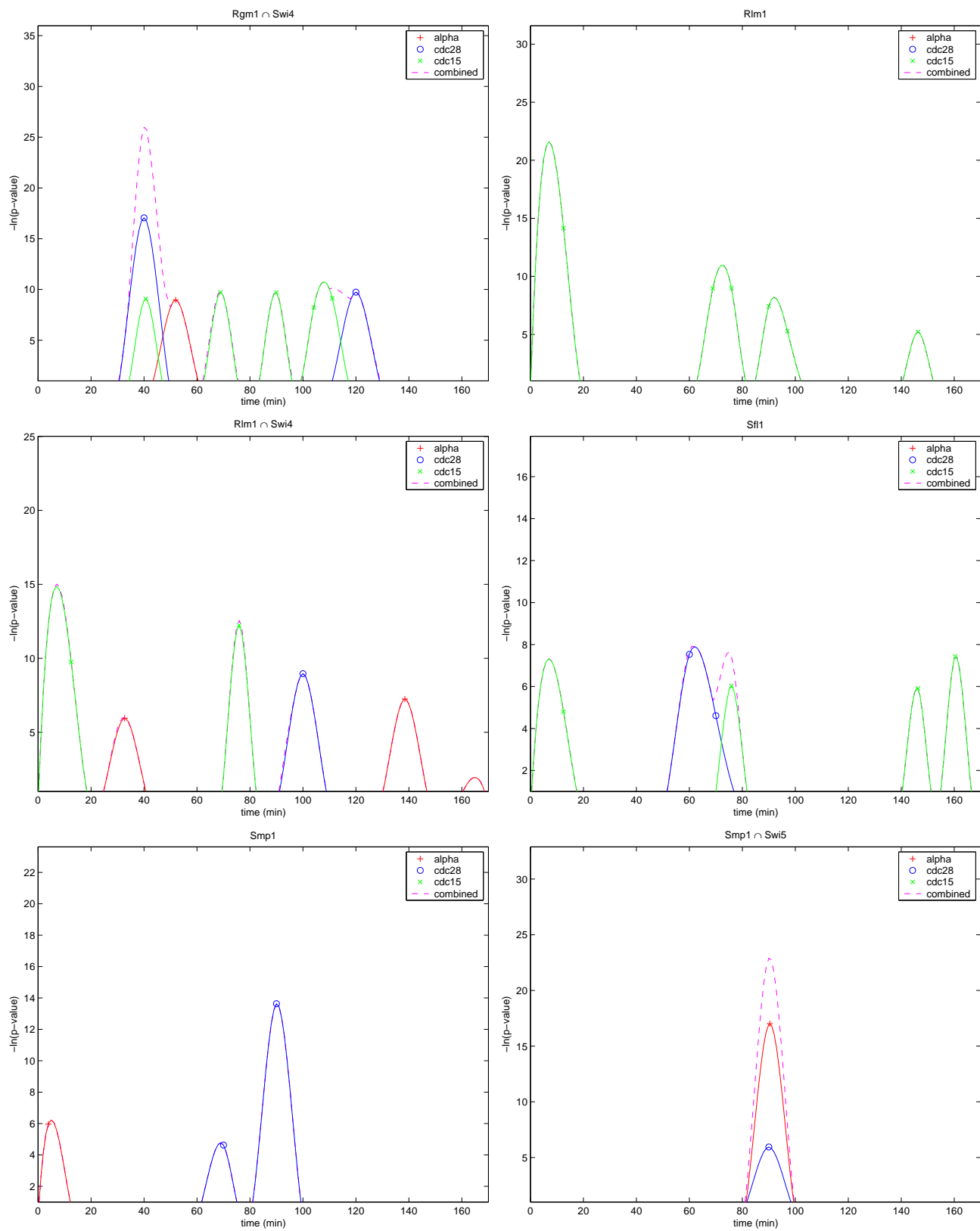

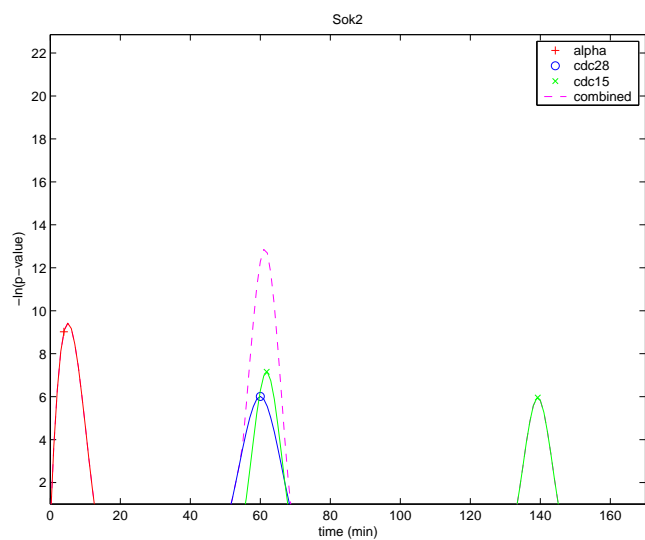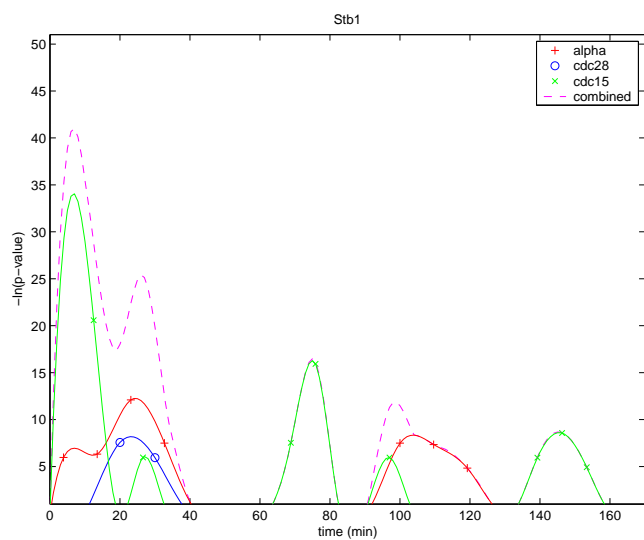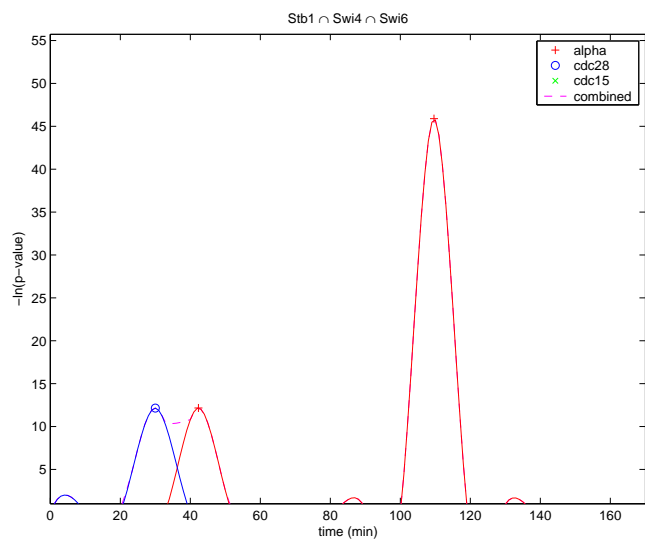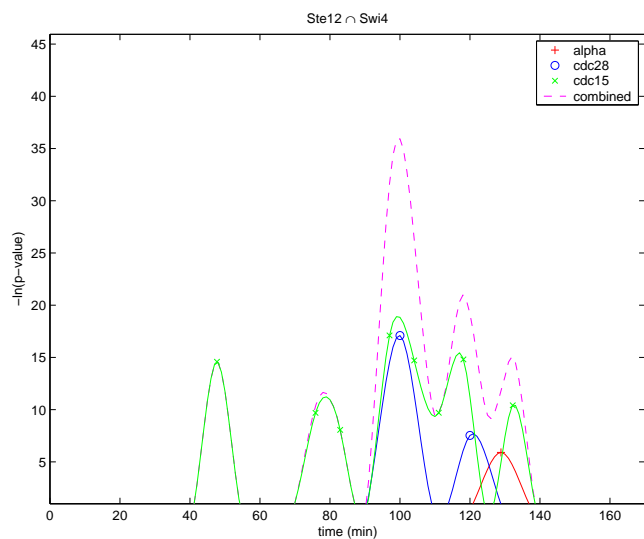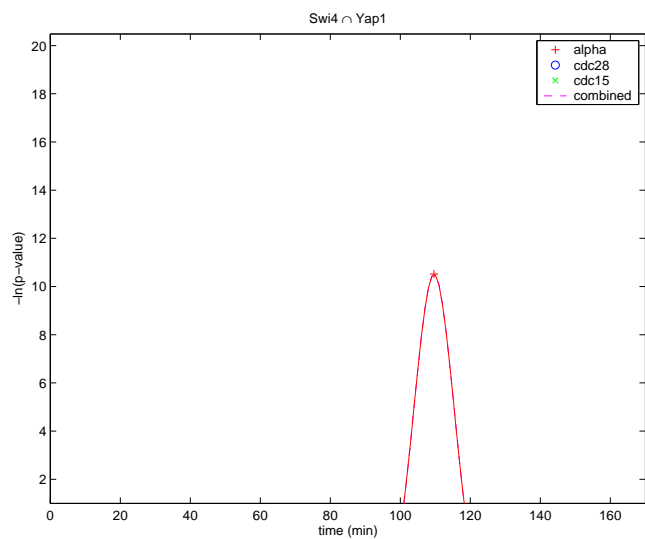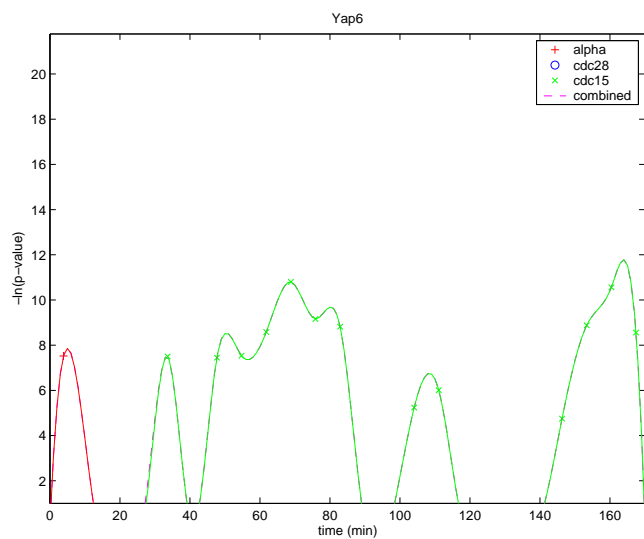

Supplement: Additional file 4 — This PDF file contains the integrated rule profiles that show a cell-cycle dependency. [file 1471-2164-10-S1-S8-S4.pdf]
