# Supplementary material for: An ensemble learning approach to reverse-engineering transcriptional regulatory networks from time-series gene expression data
Source: BMC Genomics. 2009 Jul 7;10(Suppl 1):S8. doi: 10.1186/1471-2164-10-S1-S8 (PMC2709269; doi:10.1186/1471-2164-10-S1-S8)
Supplement: Additional file 5 — This PDF file contains the integrated rule profiles that do not show a clear cell-cycle dependency. [file 1471-2164-10-S1-S8-S5.pdf]

Supplementary Table 3. Regulatory rules learned from CDC15 data set.

30min	p
Swi4 $\geq$ 3.61 $\cap$ Swi6 $\geq$ 3.08	8.42E-12
Swi4 $\geq$ 2.34	4.93E-10
Stb1 $\geq$ 3.91	1.15E-9
Stb1 $\geq$ 3.91 $\cap$ Swi6 $\geq$ 2.32	1.85E-9
Stb1 $\geq$ 2.73 $\cap$ Swi4 $\geq$ 3.61	1.85E-9
Mbp1 $\geq$ 2.85	2.95E-9
Swi6 $\geq$ 5.71	1.57E-8
Ace2 $\geq$ 17.83	3.48E-7
Mbp1 $\geq$ 3.12 $\cap$ Swi6 $\geq$ 2.56	6.54E-7
Rlm1 $\geq$ 2.38	7.09E-7
Mbp1 $\geq$ 2.3 $\cap$ Swi4 $\geq$ 3.61	3.65E-6
Fkh1 $\geq$ 3.51	1.96E-5
Rlm1 $\geq$ 2.3 $\cap$ Swi4 $\geq$ 3.61	5.83E-5
Cin5 $\geq$ 4.34	1.01E-4
Cin5 $\geq$ 2.3 $\cap$ Mbp1 $\geq$ 2.85	1.01E-4
Fkh1 $\geq$ 2.66 $\cap$ Rlm1 $\geq$ 2.3	1.06E-4
Ace2 $\geq$ 4.42 $\cap$ Fkh1 $\geq$ 2.56	1.06E-4
Cin5 $\geq$ 4.34 $\cap$ Yap6 $\geq$ 3.17	2.69E-4
Ino4 $\geq$ 2.3 $\cap$ Swi4 $\geq$ 3.61	2.77E-4
Cin5 $\geq$ 2.3 $\cap$ Rlm1 $\geq$ 2.38	5.05E-4
Sfl1 $\geq$ 2.32 $\cap$ Stb1 $\geq$ 3.47	6.89E-4

50min	p
Swi4 $\geq$ 3.77 $\cap$ Swi6 $\geq$ 3.08	6.66E-18
Swi4 $\geq$ 6.73	2.61E-15
Swi6 $\geq$ 2.56	9.67E-15
Mbp1 $\geq$ 2.3	6.3E-12
Mbp1 $\geq$ 2.72 $\cap$ Swi6 $\geq$ 2.56	9.19E-11
Mbp1 $\geq$ 2.3 $\cap$ Swi4 $\geq$ 3.77	1.13E-10
Mal13 $\geq$ 2.39 $\cap$ Swi4 $\geq$ 3.77	1.53E-10
Stb1 $\geq$ 2.43 $\cap$ Swi6 $\geq$ 2.32	6.59E-10
Ash1 $\geq$ 2.73 $\cap$ Swi6 $\geq$ 2.56	2.25E-8
Stb1 $\geq$ 2.43 $\cap$ Swi4 $\geq$ 3.77	2.44E-8
Fkh2 $\geq$ 2.4 $\cap$ Swi6 $\geq$ 2.32	1.5E-7
Fkh2 $\geq$ 4.79 $\cap$ Mbp1 $\geq$ 2.38	5.13E-6
Fkh1 $\geq$ 3.65 $\cap$ Fkh2 $\geq$ 4.79	5.89E-5
Fkh2 $\geq$ 7.49	6.07E-5
Hir2 $\geq$ 5.2	2.72E-4
Hir1 $\geq$ 3.65	5.11E-4
Fkh1 $\geq$ 2.66 $\cap$ Swi6 $\geq$ 2.56	5.31E-4

70min	p
Swi4 $\geq$ 2.33	3.54E-13
Ash1 $\geq$ 2.8 $\cap$ Swi6 $\geq$ 2.32	1.63E-11
Swi6 $\geq$ 2.32	5.51E-10
Mal13 $\geq$ 2.39 $\cap$ Swi4 $\geq$ 3.73	2.62E-9
Swi4 $\geq$ 2.33 $\cap$ Swi6 $\geq$ 2.35	6.64E-9
Ino4 $\geq$ 3.1	1.3E-8
Hsf1 $\geq$ 3.32 $\cap$ Swi4 $\geq$ 3.73	2.49E-8
Ash1 $\geq$ 2.83 $\cap$ Swi4 $\geq$ 5.12	3.42E-8
Mbp1 $\geq$ 2.3 $\cap$ Swi4 $\geq$ 2.33	3.64E-8
Ino4 $\geq$ 2.3 $\cap$ Swi4 $\geq$ 2.33	1.93E-7
Ash1 $\geq$ 2.8 $\cap$ Mal13 $\geq$ 2.39 $\cap$ Swi4 $\geq$ 2.33	4.3E-7
Ash1 $\geq$ 2.8 $\cap$ Mbp1 $\geq$ 2.81	9.47E-7
Ino4 $\geq$ 2.39 $\cap$ Met4 $\geq$ 2.7	9.96E-7
Fkh2 $\geq$ 2.33 $\cap$ Swi4 $\geq$ 2.33	5.08E-6
Hir2 $\geq$ 3.1 $\cap$ Swi4 $\geq$ 2.33	5.29E-6
Met4 $\geq$ 5.08 $\cap$ Swi4 $\geq$ 2.33	5.4E-6
Hir2 $\geq$ 3.1 $\cap$ Met4 $\geq$ 5.08	5.45E-6
Gat1 $\geq$ 2.41 $\cap$ Met4 $\geq$ 2.7	1.13E-5
Met4 $\geq$ 2.7	2.37E-5
Gat1 $\geq$ 2.4 $\cap$ Ino4 $\geq$ 3.35	3.01E-5
Sko1 $\geq$ 2.42 $\cap$ Swi4 $\geq$ 3.73	6.19E-5
Mth1 $\geq$ 2.3 $\cap$ Swi4 $\geq$ 2.33 $\cap$ Swi6 $\geq$ 3.19	6.38E-5
Ash1 $\geq$ 2.8 $\cap$ Swi4 $\geq$ 2.33 $\cap$ Swi6 $\geq$ 3.08	6.38E-5
Hir2 $\geq$ 3.06	1.16E-4
Gat1 $\geq$ 2.4 $\cap$ Msn2 $\geq$ 2.42	2.92E-4
Ash1 $\geq$ 2.8 $\cap$ Fkh2 $\geq$ 2.8 $\cap$ Swi4 $\geq$ 2.33	2.92E-4
Msn2 $\geq$ 2.42 $\cap$ Msn4 $\geq$ 2.3	5.7E-4
Gat1 $\geq$ 2.4 $\cap$ Ino2 $\geq$ 2.45	5.7E-4
Fkh2 $\geq$ 2.33 $\cap$ Mbp1 $\geq$ 3.3	5.7E-4
Gat1 $\geq$ 2.4 $\cap$ Ino2 $\geq$ 2.8 $\cap$ Ino4 $\geq$ 3.35	8.17E-4
Ino4 $\geq$ 2.39 $\cap$ Swi6 $\geq$ 2.32	8.42E-4

80min	p
Swi4 $\geq$ 4.61	1.92E-13
Hsf1 $\geq$ 3.19 $\cap$ Swi4 $\geq$ 2.33	3.33E-9
Ash1 $\geq$ 3.04	3.53E-8
Fkh2 $\geq$ 4.07	1.87E-7
Swi4 $\geq$ 4.61 $\cap$ Swi6 $\geq$ 3.08	2.41E-7
Fkh2 $\geq$ 4.07 $\cap$ Swi4 $\geq$ 4.61	2.48E-7
Grf10(pho2) $\geq$ 2.44 $\cap$ Swi4 $\geq$ 4.61	5.08E-6
Mal13 $\geq$ 3.54 $\cap$ Swi4 $\geq$ 4.61	5.13E-6
Mal13 $\geq$ 3.17	6.07E-5
Ino4 $\geq$ 3.08 $\cap$ Swi4 $\geq$ 2.33	1.17E-4
Met4 $\geq$ 2.72	1.2E-4
Met4 $\geq$ 3.1 $\cap$ Rgm1 $\geq$ 2.3	2.8E-4
Ash1 $\geq$ 2.83 $\cap$ Met4 $\geq$ 2.33	5.42E-4
Yap6 $\geq$ 3.0	5.53E-4
Mal13 $\geq$ 2.55 $\cap$ Met4 $\geq$ 2.33	5.53E-4
Gat1 $\geq$ 2.5 $\cap$ Met4 $\geq$ 2.3	5.53E-4
Gat1 $\geq$ 2.39 $\cap$ Msn4 $\geq$ 2.3	5.53E-4
Fkh1 $\geq$ 3.41	5.53E-4
Cin5 $\geq$ 2.63 $\cap$ Met4 $\geq$ 2.3	6.56E-4

90min	p
Swi4 $\geq$ 2.7	1.32E-8
Ash1 $\geq$ 2.63	1.59E-8
Hsf1 $\geq$ 3.19 $\cap$ Swi4 $\geq$ 4.61	3.53E-8
Fkh2 $\geq$ 3.24	3.56E-8
Fkh2 $\geq$ 4.07 $\cap$ Swi4 $\geq$ 2.33	2.71E-7
Ndd1 $\geq$ 4.14	3.55E-7
Ash1 $\geq$ 2.63 $\cap$ Fkh2 $\geq$ 3.24	4.38E-7
Ino4 $\geq$ 2.3 $\cap$ Swi4 $\geq$ 2.7	9.47E-7
Ash1 $\geq$ 2.63 $\cap$ Swi4 $\geq$ 2.7	9.47E-7
Cin5 $\geq$ 2.3	1.59E-6
Ash1 $\geq$ 2.63 $\cap$ Swi6 $\geq$ 2.35	4.64E-6
Ash1 $\geq$ 2.83 $\cap$ Mal13 $\geq$ 3.17	5.29E-6
Fkh2 $\geq$ 2.58 $\cap$ Hsf1 $\geq$ 3.19 $\cap$ Swi4 $\geq$ 4.66	5.34E-6
Mal13 $\geq$ 2.39	2.21E-5
Mth1 $\geq$ 2.92 $\cap$ Swi4 $\geq$ 4.61	2.98E-5
Mal13 $\geq$ 2.39 $\cap$ Swi4 $\geq$ 2.7	6.31E-5
Fkh2 $\geq$ 4.07 $\cap$ Ste12 $\geq$ 2.65	6.31E-5
Grf10(pho2) $\geq$ 3.73 $\cap$ Swi4 $\geq$ 4.61	6.38E-5
Dal82 $\geq$ 2.59 $\cap$ Ecm22 $\geq$ 2.47 $\cap$ Mth1 $\geq$ 2.35	6.38E-5
Rgm1 $\geq$ 2.3 $\cap$ Swi4 $\geq$ 2.33	1.14E-4
Cin5 $\geq$ 2.78 $\cap$ Swi4 $\geq$ 2.33	1.14E-4
Ash1 $\geq$ 2.75 $\cap$ Ino4 $\geq$ 2.3	1.19E-4
Ecm22 $\geq$ 2.4 $\cap$ Swi4 $\geq$ 2.33	2.86E-4
Cin5 $\geq$ 2.63 $\cap$ Met4 $\geq$ 2.3	5.64E-4
Ash1 $\geq$ 2.83 $\cap$ Ecm22 $\geq$ 2.4	5.64E-4
Swi4 $\geq$ 2.33 $\cap$ Swi6 $\geq$ 2.83	5.81E-4
Dal82 $\geq$ 2.54	5.93E-4
Mcm1 $\geq$ 4.85 $\cap$ Ndd1 $\geq$ 4.14	7.17E-4
Ixr1 $\geq$ 2.65 $\cap$ Mth1 $\geq$ 2.3	7.32E-4
Fkh1 $\geq$ 8.25 $\cap$ Fkh2 $\geq$ 4.07	7.32E-4
Dal82 $\geq$ 2.59 $\cap$ Mss11 $\geq$ 3.3	7.32E-4
Cin5 $\geq$ 2.63 $\cap$ Hsf1 $\geq$ 3.65	7.32E-4
Ash1 $\geq$ 2.75 $\cap$ Cbf1 $\geq$ 5.47	7.32E-4
Fkh2 $\geq$ 3.24 $\cap$ Mcm1 $\geq$ 8.47	7.39E-4
Cin5 $\geq$ 2.67 $\cap$ Mal13 $\geq$ 3.17	7.39E-4
Cha4 $\geq$ 3.17 $\cap$ Fkh2 $\geq$ 2.58	7.39E-4
Hir2 $\geq$ 2.31 $\cap$ Yap6 $\geq$ 3.0	7.47E-4
Fkh1 $\geq$ 8.25	7.47E-4

100min	p
Swi4 $\geq$ 4.91	4.03E-9
Fkh2 $\geq$ 4.07	8.28E-9
Ndd1 $\geq$ 3.24	1.81E-8
Fkh2 $\geq$ 4.07 $\cap$ Swi4 $\geq$ 2.73	2.46E-8
Fkh2 $\geq$ 5.17 $\cap$ Ndd1 $\geq$ 4.34	3.64E-8
Ash1 $\geq$ 2.44 $\cap$ Swi4 $\geq$ 2.73	2.18E-7
Rap1 $\geq$ 2.51 $\cap$ Swi4 $\geq$ 4.91	4.43E-7
Ste12 $\geq$ 2.53 $\cap$ Swi4 $\geq$ 4.91	4.61E-7
Ino4 $\geq$ 2.47 $\cap$ Swi4 $\geq$ 4.91	1.13E-6
Ndd1 $\geq$ 3.24 $\cap$ Swi6 $\geq$ 3.19	5.29E-6
Ash1 $\geq$ 2.63 $\cap$ Swi6 $\geq$ 2.3	5.34E-6
Ash1 $\geq$ 2.54 $\cap$ Ino4 $\geq$ 2.47 $\cap$ Swi4 $\geq$ 4.91	6.38E-5
Met4 $\geq$ 2.32 $\cap$ Rgm1 $\geq$ 2.3	1.22E-4
Fkh2 $\geq$ 3.24 $\cap$ Rap1 $\geq$ 2.47	1.22E-4
Cin5 $\geq$ 2.3 $\cap$ Swi4 $\geq$ 2.73	1.22E-4
Sum1 $\geq$ 2.3	1.42E-4
Hsf1 $\geq$ 2.92 $\cap$ Swi4 $\geq$ 5.12	2.92E-4
Fkh2 $\geq$ 4.07 $\cap$ Mcm1 $\geq$ 4.61	2.92E-4
Cin5 $\geq$ 2.67	5E-4
Mcm1 $\geq$ 4.61	5.7E-4
Hap3 $\geq$ 2.44 $\cap$ Sum1 $\geq$ 2.38	5.7E-4
Fkh2 $\geq$ 3.24 $\cap$ Rgm1 $\geq$ 2.33	5.7E-4
Ash1 $\geq$ 4.07	5.7E-4
Yap6 $\geq$ 3.0	5.81E-4
Fkh1 $\geq$ 3.27 $\cap$ Fkh2 $\geq$ 3.24	5.81E-4
Cin5 $\geq$ 2.76 $\cap$ Ndd1 $\geq$ 2.53	5.81E-4
Mcm1 $\geq$ 4.61 $\cap$ Ndd1 $\geq$ 2.53	7.17E-4
Rim101 $\geq$ 2.3 $\cap$ Sum1 $\geq$ 2.3	8.33E-4

110min	p
Ndd1 $\geq$ 3.65	2.44E-8
Mcm1 $\geq$ 4.61 $\cap$ Ndd1 $\geq$ 4.14	3.77E-7
Ash1 $\geq$ 2.63 $\cap$ Ndd1 $\geq$ 3.65	3.89E-7
Ash1 $\geq$ 2.63	1.15E-6
Fkh2 $\geq$ 2.6	1.4E-6
Cin5 $\geq$ 2.3	1.47E-6
Fkh2 $\geq$ 2.6 $\cap$ Ndd1 $\geq$ 2.53	4.2E-6
Phd1 $\geq$ 2.6	4.46E-6
Swi4 $\geq$ 2.33	7.07E-6
Mcm1 $\geq$ 4.61	2.19E-5
Hsf1 $\geq$ 2.67 $\cap$ Swi4 $\geq$ 2.7	9.05E-5
Cin5 $\geq$ 2.67 $\cap$ Met4 $\geq$ 2.3	1.11E-4
Ash1 $\geq$ 2.83 $\cap$ Met4 $\geq$ 2.33	1.11E-4
Met4 $\geq$ 2.3 $\cap$ Rgm1 $\geq$ 2.3	1.15E-4
Met4 $\geq$ 2.33 $\cap$ Ndd1 $\geq$ 4.14	2.77E-4
Ino4 $\geq$ 2.47 $\cap$ Swi4 $\geq$ 2.7	2.86E-4
Yap6 $\geq$ 3.0	5.37E-4
Nrg1 $\geq$ 2.48	6.43E-4
Aro80 $\geq$ 2.94 $\cap$ Met4 $\geq$ 2.96	7.17E-4
Cin5 $\geq$ 4.65 $\cap$ Ndd1 $\geq$ 3.65	7.25E-4
Cin5 $\geq$ 3.12 $\cap$ Hsf1 $\geq$ 3.65	7.25E-4
Cin5 $\geq$ 2.67 $\cap$ Rgm1 $\geq$ 2.3	7.25E-4
Dal82 $\geq$ 2.54 $\cap$ Ndd1 $\geq$ 3.65	7.54E-4

120min	p
Ndd1 $\geq$ 3.54 $\cap$ Swi4 $\geq$ 2.33	1.35E-9
Fkh2 $\geq$ 4.72 $\cap$ Swi4 $\geq$ 2.73	3.47E-9
Ndd1 $\geq$ 11.18	3.5E-9
Swi4 $\geq$ 2.33	6.07E-9
Ash1 $\geq$ 4.2	4.14E-8
Fkh2 $\geq$ 4.72	5.87E-8
Cup9 $\geq$ 2.3 $\cap$ Swi4 $\geq$ 2.33	5.04E-7
Fkh2 $\geq$ 4.72 $\cap$ Ndd1 $\geq$ 2.53	1.33E-6
Ash1 $\geq$ 2.59 $\cap$ Phd1 $\geq$ 2.67	1.33E-6
Ino4 $\geq$ 2.47 $\cap$ Swi4 $\geq$ 2.33	1.37E-6
Met4 $\geq$ 3.51 $\cap$ Skn7 $\geq$ 3.96 $\cap$ Swi4 $\geq$ 2.33	5.73E-6
Hir2 $\geq$ 2.65 $\cap$ Swi4 $\geq$ 2.33	5.73E-6
Rap1 $\geq$ 3.3 $\cap$ Swi4 $\geq$ 2.53	6.39E-6
Cin5 $\geq$ 2.78 $\cap$ Swi4 $\geq$ 2.53	2.95E-5
Ace2 $\geq$ 2.43 $\cap$ Swi4 $\geq$ 2.33	2.95E-5
Sum1 $\geq$ 2.38	4.63E-5
Cin5 $\geq$ 2.78 $\cap$ Cup9 $\geq$ 2.3 $\cap$ Swi4 $\geq$ 2.33	6.44E-5
Ash1 $\geq$ 4.2 $\cap$ Ecm22 $\geq$ 2.4	6.44E-5
Phd1 $\geq$ 5.08	1.32E-4
Hap3 $\geq$ 2.31 $\cap$ Sum1 $\geq$ 2.81	1.32E-4
Dot6 $\geq$ 2.3 $\cap$ Sum1 $\geq$ 2.81	1.32E-4
Ash1 $\geq$ 3.38 $\cap$ Swi4 $\geq$ 2.33	1.32E-4
Met4 $\geq$ 3.51 $\cap$ Swi4 $\geq$ 2.33	1.35E-4
Hap3 $\geq$ 2.31 $\cap$ Rtg3 $\geq$ 2.3	1.35E-4
Yap6 $\geq$ 3.0	1.88E-4
Phd1 $\geq$ 5.4 $\cap$ Swi4 $\geq$ 2.33	3.01E-4
Rtg3 $\geq$ 2.3 $\cap$ Sum1 $\geq$ 2.38	5.99E-4
Rim101 $\geq$ 2.53 $\cap$ Rtg3 $\geq$ 3.61	5.99E-4
Rim101 $\geq$ 2.43 $\cap$ Sum1 $\geq$ 2.43	5.99E-4
Fkh1 $\geq$ 4.93	5.99E-4
Ecm22 $\geq$ 2.34 $\cap$ Swi4 $\geq$ 2.33	5.99E-4
Swi4 $\geq$ 2.33 $\cap$ Swi6 $\geq$ 2.83	6.11E-4
Fkh1 $\geq$ 3.22 $\cap$ Swi4 $\geq$ 2.53	6.11E-4
Sok2 $\geq$ 4.34	7.77E-4

130min	p
Fkh2 $\geq$ 2.4	3.84E-6
Cin5 $\geq$ 2.56 $\cap$ Met4 $\geq$ 2.44	4.88E-6
Met4 $\geq$ 2.65	5.79E-6
Cin5 $\geq$ 2.56	5.79E-6
Swi4 $\geq$ 2.7	5.9E-6
Yap6 $\geq$ 3.3	2.04E-5
Met4 $\geq$ 2.3 $\cap$ Rgm1 $\geq$ 2.3	2.14E-5
Ino4 $\geq$ 2.58 $\cap$ Swi4 $\geq$ 2.7	2.65E-5
Ace2 $\geq$ 19.26	5.77E-5
Swi4 $\geq$ 2.7 $\cap$ Swi6 $\geq$ 2.83	5.89E-5
Rgm1 $\geq$ 2.3 $\cap$ Swi4 $\geq$ 2.7	6.01E-5
Hsf1 $\geq$ 2.4 $\cap$ Swi4 $\geq$ 2.7	1.04E-4
Rlm1 $\geq$ 2.41	1.28E-4
Cin5 $\geq$ 3.12 $\cap$ Hsf1 $\geq$ 2.4 $\cap$ Swi4 $\geq$ 2.7	2.75E-4
Swi6 $\geq$ 2.32	3.71E-4
Ash1 $\geq$ 2.72	3.71E-4
Fkh2 $\geq$ 2.94 $\cap$ Met4 $\geq$ 2.44	4.95E-4
Ash1 $\geq$ 2.72 $\cap$ Met4 $\geq$ 2.65	5.16E-4
Stb1 $\geq$ 2.73	5.37E-4
Aro80 $\geq$ 2.94 $\cap$ Met4 $\geq$ 2.75	7.03E-4
Rgm1 $\geq$ 2.51 $\cap$ Swi6 $\geq$ 2.32	7.1E-4
Cin5 $\geq$ 2.67 $\cap$ Rgm1 $\geq$ 2.3	7.1E-4
Cin5 $\geq$ 2.56 $\cap$ Fkh1 $\geq$ 3.65	7.1E-4

140min	p
Swi4 $\geq$ 2.7	2.93E-13
Swi4 $\geq$ 3.61 $\cap$ Swi6 $\geq$ 2.35	1.03E-10
Swi6 $\geq$ 2.32	1.17E-9
Stb1 $\geq$ 2.73 $\cap$ Swi4 $\geq$ 2.7	1.94E-9
Stb1 $\geq$ 2.96	1.21E-7
Fkh2 $\geq$ 4.07	6.88E-7
Mbp1 $\geq$ 2.3 $\cap$ Swi4 $\geq$ 2.7	3.73E-6
Hsf1 $\geq$ 2.4 $\cap$ Swi4 $\geq$ 2.7	4.46E-6
Ace2 $\geq$ 17.83	4.74E-6
Cin5 $\geq$ 3.12 $\cap$ Swi4 $\geq$ 2.7	4.79E-6
Rlm1 $\geq$ 2.3 $\cap$ Swi4 $\geq$ 2.7	4.88E-6
Ste12 $\geq$ 2.44 $\cap$ Swi4 $\geq$ 2.7	6.13E-5
Yap6 $\geq$ 3.38	1.05E-4
Fkh1 $\geq$ 2.66 $\cap$ Rlm1 $\geq$ 2.58	1.05E-4
Cin5 $\geq$ 4.34	1.11E-4
Rlm1 $\geq$ 2.59	1.25E-4
Cin5 $\geq$ 4.34 $\cap$ Yap6 $\geq$ 3.65	2.77E-4
Ash1 $\geq$ 2.72 $\cap$ Swi4 $\geq$ 2.7	5.21E-4
Cin5 $\geq$ 2.56 $\cap$ Met4 $\geq$ 2.44	5.37E-4
Ace2 $\geq$ 2.36 $\cap$ Rlm1 $\geq$ 2.59	5.37E-4
Ace2 $\geq$ 2.36 $\cap$ Fkh2 $\geq$ 2.94	5.37E-4
Rlm1 $\geq$ 2.59 $\cap$ Sfl1 $\geq$ 3.35	6.89E-4
Ino4 $\geq$ 3.1 $\cap$ Ste12 $\geq$ 2.44 $\cap$ Swi4 $\geq$ 2.7	6.89E-4
Rox1 $\geq$ 2.6 $\cap$ Swi4 $\geq$ 2.7	6.96E-4
Sfl1 $\geq$ 2.32 $\cap$ Stb1 $\geq$ 3.47	7.03E-4
Rap1 $\geq$ 2.51 $\cap$ Swi4 $\geq$ 4.42	7.03E-4

150min	p
Swi4 $\geq$ 4.34 $\cap$ Swi6 $\geq$ 3.24	5.78E-17
Swi4 $\geq$ 4.34	3.96E-14
Stb1 $\geq$ 2.43 $\cap$ Swi4 $\geq$ 4.34	1.99E-11
Met4 $\geq$ 2.92	2.66E-7
Hsf1 $\geq$ 2.86 $\cap$ Swi4 $\geq$ 4.34	2.66E-7
Mal13 $\geq$ 2.39 $\cap$ Swi4 $\geq$ 4.34	1.24E-6
Fkh2 $\geq$ 5.74 $\cap$ Mbp1 $\geq$ 2.85	5.67E-6
Mal13 $\geq$ 2.36 $\cap$ Met4 $\geq$ 3.1	2.73E-5
Cin5 $\geq$ 2.56 $\cap$ Met4 $\geq$ 2.44	2.73E-5
Cin5 $\geq$ 5.43	2.81E-5
Ash1 $\geq$ 2.83 $\cap$ Met4 $\geq$ 2.65	2.81E-5
Cin5 $\geq$ 3.22 $\cap$ Yap6 $\geq$ 3.0	2.89E-5
Hsf1 $\geq$ 3.32 $\cap$ Mal13 $\geq$ 2.43 $\cap$ Swi4 $\geq$ 4.34	6.44E-5
Ace2 $\geq$ 17.83	6.57E-5
Fkh2 $\geq$ 5.74	1.28E-4
Fkh2 $\geq$ 2.4 $\cap$ Swi4 $\geq$ 4.34	1.31E-4
Cin5 $\geq$ 2.56 $\cap$ Fkh1 $\geq$ 2.66	1.31E-4
Yap6 $\geq$ 3.0	1.48E-4
Ste12 $\geq$ 2.44 $\cap$ Swi4 $\geq$ 4.34	3.13E-4
Nrg1 $\geq$ 2.5 $\cap$ Yap6 $\geq$ 3.0	5.99E-4
Cin5 $\geq$ 2.56 $\cap$ Mbp1 $\geq$ 2.85	5.99E-4
Cbf1 $\geq$ 4.74	5.99E-4
Mbp1 $\geq$ 2.85 $\cap$ Met4 $\geq$ 2.44	6.11E-4
Fkh2 $\geq$ 2.94 $\cap$ Met4 $\geq$ 2.44	6.11E-4
Ash1 $\geq$ 3.38	6.76E-4
Phd1 $\geq$ 6.44	8.5E-4
Met4 $\geq$ 2.65 $\cap$ Sko1 $\geq$ 2.3	8.59E-4

160min	p
Swi4 $\geq$ 4.66 $\cap$ Swi6 $\geq$ 2.35	2.33E-17
Swi4 $\geq$ 4.66	3.14E-17
Swi6 $\geq$ 2.56	1.29E-10
Mal13 $\geq$ 2.43 $\cap$ Swi4 $\geq$ 4.66	2.01E-10
Stb1 $\geq$ 2.73 $\cap$ Swi4 $\geq$ 4.66	2.05E-10
Ino4 $\geq$ 2.63 $\cap$ Swi4 $\geq$ 4.66	2.13E-10
Fkh2 $\geq$ 5.91	5.18E-6
Fkh2 $\geq$ 4.07 $\cap$ Swi4 $\geq$ 4.66	6.07E-5
Ace2 $\geq$ 17.83	6.07E-5
Rgm1 $\geq$ 2.38 $\cap$ Swi4 $\geq$ 4.66	6.13E-5
Hir2 $\geq$ 2.72	1.17E-4
Fkh2 $\geq$ 5.91 $\cap$ Mbp1 $\geq$ 2.85	1.17E-4
Fkh1 $\geq$ 3.65	5.53E-4
Fkh1 $\geq$ 3.27 $\cap$ Mbp1 $\geq$ 2.85	5.53E-4
Fkh1 $\geq$ 2.3 $\cap$ Rlm1 $\geq$ 2.59	5.53E-4
Fkh1 $\geq$ 3.27 $\cap$ Fkh2 $\geq$ 5.91	5.7E-4
Rlm1 $\geq$ 2.59	5.93E-4
Hsf1 $\geq$ 3.44 $\cap$ Swi4 $\geq$ 4.66	8.01E-4

170min	p
Swi4 $\geq$ 4.42	3.79E-20
Swi4 $\geq$ 4.42 $\cap$ Swi6 $\geq$ 3.08	3.33E-17
Swi6 $\geq$ 3.24	6.78E-13
Stb1 $\geq$ 2.43 $\cap$ Swi4 $\geq$ 5.12	1.39E-12
Ash1 $\geq$ 2.73 $\cap$ Swi4 $\geq$ 2.54	1.47E-9
Mbp1 $\geq$ 2.72 $\cap$ Swi4 $\geq$ 2.34	1.57E-9
Hsf1 $\geq$ 2.67 $\cap$ Swi4 $\geq$ 2.34	7.95E-9
Ste12 $\geq$ 2.32 $\cap$ Swi4 $\geq$ 3.32	3.71E-8
Fkh2 $\geq$ 4.98 $\cap$ Mbp1 $\geq$ 2.38	2.88E-7
Met4 $\geq$ 2.65 $\cap$ Swi4 $\geq$ 2.34	1.13E-6
Mal13 $\geq$ 2.43 $\cap$ Swi4 $\geq$ 3.32	1.19E-6
Fkh1 $\geq$ 2.66 $\cap$ Mbp1 $\geq$ 3.73	3.02E-6
Fkh2 $\geq$ 4.98	3.54E-6
Hir2 $\geq$ 2.72 $\cap$ Met4 $\geq$ 5.13	5.56E-6
Met4 $\geq$ 5.08	5.62E-6
Hir2 $\geq$ 2.51 $\cap$ Swi4 $\geq$ 2.34	2.7E-5
Ino4 $\geq$ 2.49 $\cap$ Swi4 $\geq$ 2.34	3.99E-5
Put3 $\geq$ 2.45 $\cap$ Swi4 $\geq$ 3.32	6.44E-5
Gat1 $\geq$ 2.5 $\cap$ Met4 $\geq$ 5.3	6.44E-5
Mbp1 $\geq$ 4.34	1.25E-4
Fkh1 $\geq$ 2.66 $\cap$ Swi4 $\geq$ 2.34	1.25E-4
Hir2 $\geq$ 2.72	1.28E-4
Hir1 $\geq$ 2.39 $\cap$ Swi4 $\geq$ 2.34	1.28E-4
Fkh1 $\geq$ 3.51 $\cap$ Fkh2 $\geq$ 2.94	1.28E-4
Hir2 $\geq$ 2.31 $\cap$ Ino4 $\geq$ 2.49	5.81E-4
Fkh2 $\geq$ 3.35 $\cap$ Swi4 $\geq$ 2.34	5.93E-4
Fkh1 $\geq$ 2.86 $\cap$ Swi6 $\geq$ 3.08	5.93E-4
Ash1 $\geq$ 3.96	5.93E-4
Ino4 $\geq$ 2.39 $\cap$ Met4 $\geq$ 2.65	7.17E-4

180min	p
Swi4 $\geq$ 4.66 $\cap$ Swi6 $\geq$ 2.35	1.11E-13
Swi4 $\geq$ 4.66	2.61E-12
Swi6 $\geq$ 2.81	4.82E-9
Ash1 $\geq$ 2.8 $\cap$ Swi6 $\geq$ 2.35	4.35E-8
Ste12 $\geq$ 2.32 $\cap$ Swi4 $\geq$ 4.66	4.05E-7
Stb1 $\geq$ 2.43 $\cap$ Swi4 $\geq$ 4.34	4.13E-7
Mbp1 $\geq$ 3.15 $\cap$ Swi6 $\geq$ 2.35	1.07E-6
Mbp1 $\geq$ 2.72 $\cap$ Swi4 $\geq$ 4.34	1.07E-6
Fkh2 $\geq$ 4.07 $\cap$ Swi4 $\geq$ 4.34	2.68E-6
Mal13 $\geq$ 3.54 $\cap$ Swi4 $\geq$ 4.34	4.93E-6
Ash1 $\geq$ 3.04 $\cap$ Swi4 $\geq$ 4.66	5.13E-6
Phd1 $\geq$ 2.67 $\cap$ Swi4 $\geq$ 4.34	2.39E-5
Grf10(pho2) $\geq$ 2.44 $\cap$ Swi4 $\geq$ 4.34	2.39E-5
Cin5 $\geq$ 2.47 $\cap$ Met4 $\geq$ 2.33	2.44E-5
Mth1 $\geq$ 2.42 $\cap$ Swi4 $\geq$ 4.34	2.49E-5
Ace2 $\geq$ 4.02 $\cap$ Fkh1 $\geq$ 3.41	5.77E-5
Cin5 $\geq$ 2.47	9.14E-5
Rap1 $\geq$ 2.51 $\cap$ Swi4 $\geq$ 4.34	1.13E-4
Cin5 $\geq$ 2.41 $\cap$ Gat1 $\geq$ 2.5	1.15E-4
Ace2 $\geq$ 4.42	1.16E-4
Rgm1 $\geq$ 2.33 $\cap$ Swi4 $\geq$ 4.66	2.69E-4
Fkh2 $\geq$ 9.12	2.72E-4
Hir2 $\geq$ 2.72	4.48E-4
Met4 $\geq$ 4.51	5.26E-4
Hir2 $\geq$ 3.1 $\cap$ Ino4 $\geq$ 2.49	5.26E-4
Cin5 $\geq$ 2.47 $\cap$ Fkh1 $\geq$ 2.81	5.26E-4
Cha4 $\geq$ 2.3 $\cap$ Sum1 $\geq$ 2.54	5.26E-4
Rtg1 $\geq$ 2.35 $\cap$ Sfp1 $\geq$ 2.9	5.31E-4
Ndd1 $\geq$ 2.76 $\cap$ Swi4 $\geq$ 4.34	5.31E-4
Abf1 $\geq$ 2.42 $\cap$ Ace2 $\geq$ 3.15	5.42E-4
Sko1 $\geq$ 2.72 $\cap$ Sok2 $\geq$ 2.65	5.76E-4
Sum1 $\geq$ 3.3	6.69E-4
Ash1 $\geq$ 2.8 $\cap$ Mth1 $\geq$ 2.42 $\cap$ Swi4 $\geq$ 4.66	7.47E-4

190min	p
Swi4 $\geq$ 2.33	3.59E-11
Hsf1 $\geq$ 3.32 $\cap$ Swi4 $\geq$ 2.33	4.75E-11
Ash1 $\geq$ 2.63 $\cap$ Swi4 $\geq$ 4.61	1.69E-10
Hsf1 $\geq$ 3.32	2.44E-8
Ash1 $\geq$ 2.63	2.53E-7
Mal13 $\geq$ 3.04 $\cap$ Swi4 $\geq$ 3.06	3.89E-7
Swi4 $\geq$ 2.33 $\cap$ Swi6 $\geq$ 2.35	4.37E-6
Swi6 $\geq$ 3.19	4.64E-6
Hir2 $\geq$ 3.1 $\cap$ Swi4 $\geq$ 2.33	4.98E-6
Grf10(pho2) $\geq$ 5.15 $\cap$ Swi4 $\geq$ 2.33	4.98E-6
Ino4 $\geq$ 2.3 $\cap$ Swi4 $\geq$ 3.06	2.14E-5
Gcn4 $\geq$ 3.08 $\cap$ Sum1 $\geq$ 3.27	6.01E-5
Ste12 $\geq$ 2.65 $\cap$ Swi4 $\geq$ 2.33	6.07E-5
Mth1 $\geq$ 2.92 $\cap$ Rgm1 $\geq$ 2.3 $\cap$ Swi4 $\geq$ 2.33	6.07E-5
Met4 $\geq$ 3.51 $\cap$ Swi4 $\geq$ 2.33	6.13E-5
Rgm1 $\geq$ 2.3 $\cap$ Swi4 $\geq$ 2.33	1.08E-4
Ash1 $\geq$ 3.04 $\cap$ Mal13 $\geq$ 2.39 $\cap$ Swi4 $\geq$ 2.33	1.13E-4
Ash1 $\geq$ 2.8 $\cap$ Swi4 $\geq$ 2.33 $\cap$ Swi6 $\geq$ 3.19	1.13E-4
Met4 $\geq$ 3.19	1.45E-4
Phd1 $\geq$ 5.08	2.83E-4
Cin5 $\geq$ 2.63	4.39E-4
Fkh2 $\geq$ 4.07 $\cap$ Swi4 $\geq$ 2.33	5.11E-4
Hir2 $\geq$ 2.72	5.31E-4
Gcn4 $\geq$ 3.08	5.48E-4
Met4 $\geq$ 3.19 $\cap$ Reb1 $\geq$ 6.38	7.1E-4
Gat1 $\geq$ 2.5 $\cap$ Phd1 $\geq$ 5.08	7.1E-4
Mal13 $\geq$ 3.04 $\cap$ Phd1 $\geq$ 2.94 $\cap$ Swi4 $\geq$ 2.33	7.17E-4
Gcn4 $\geq$ 3.08 $\cap$ Swi4 $\geq$ 2.33	7.25E-4
Cin5 $\geq$ 2.3 $\cap$ Mal13 $\geq$ 3.32	7.32E-4
Met4 $\geq$ 2.33 $\cap$ Phd1 $\geq$ 5.08	7.47E-4

200min	p
Swi4 $\geq$ 2.33	7.58E-10
Swi4 $\geq$ 4.66 $\cap$ Swi6 $\geq$ 2.86	2.35E-9
Fkh2 $\geq$ 4.07 $\cap$ Swi4 $\geq$ 4.66	2.95E-8
Ash1 $\geq$ 2.63 $\cap$ Swi4 $\geq$ 3.73	3.29E-8
Ash1 $\geq$ 2.8 $\cap$ Swi6 $\geq$ 2.35	1.71E-7
Ste12 $\geq$ 2.32 $\cap$ Swi4 $\geq$ 4.66	3.7E-7
Ash1 $\geq$ 3.04	3.89E-7
Met4 $\geq$ 2.72	4.89E-7
Hsf1 $\geq$ 2.86 $\cap$ Swi4 $\geq$ 2.33	9.38E-7
Ash1 $\geq$ 2.8 $\cap$ Swi4 $\geq$ 4.66 $\cap$ Swi6 $\geq$ 2.86	2.42E-6
Ash1 $\geq$ 2.8 $\cap$ Mbp1 $\geq$ 3.12	4.37E-6
Fkh2 $\geq$ 4.07	4.55E-6
Phd1 $\geq$ 4.02	7.5E-6
Ndd1 $\geq$ 3.24 $\cap$ Swi4 $\geq$ 2.33	2.17E-5
Mbp1 $\geq$ 3.12 $\cap$ Swi4 $\geq$ 2.33	2.17E-5
Ino4 $\geq$ 3.06 $\cap$ Swi4 $\geq$ 2.33	2.17E-5
Met4 $\geq$ 2.33 $\cap$ Phd1 $\geq$ 5.08	5.55E-5
Grf10(pho2) $\geq$ 5.15 $\cap$ Swi4 $\geq$ 4.66	5.55E-5
Ash1 $\geq$ 2.8 $\cap$ Mth1 $\geq$ 2.92 $\cap$ Swi4 $\geq$ 4.66	5.55E-5
Mth1 $\geq$ 2.42 $\cap$ Swi4 $\geq$ 4.66	1.05E-4
Met4 $\geq$ 3.51 $\cap$ Swi4 $\geq$ 2.33	1.05E-4
Ash1 $\geq$ 2.63 $\cap$ Met4 $\geq$ 3.27	1.05E-4
Ash1 $\geq$ 3.08 $\cap$ Phd1 $\geq$ 5.08	2.59E-4
Ino2 $\geq$ 3.61	5.05E-4
Ino2 $\geq$ 2.31 $\cap$ Swi4 $\geq$ 2.33	5.05E-4
Gat1 $\geq$ 2.38 $\cap$ Met4 $\geq$ 2.72	5.05E-4
Grf10(pho2) $\geq$ 2.44 $\cap$ Met4 $\geq$ 2.72	5.16E-4
Ash1 $\geq$ 2.63 $\cap$ Ino4 $\geq$ 2.47	5.26E-4
Cin5 $\geq$ 2.58	6.43E-4

210min	p
Swi4 $\geq$ 3.06	1.92E-8
Ndd1 $\geq$ 3.24	1.21E-7
Fkh2 $\geq$ 2.8	1.21E-7
Ash1 $\geq$ 2.8	2.11E-7
Ash1 $\geq$ 2.63 $\cap$ Ndd1 $\geq$ 3.51	3.25E-7
Hsf1 $\geq$ 3.38	1.83E-5
Ndd1 $\geq$ 2.53 $\cap$ Swi4 $\geq$ 2.33	1.92E-5
Hsf1 $\geq$ 2.65 $\cap$ Swi4 $\geq$ 3.06	1.92E-5
Ash1 $\geq$ 2.63 $\cap$ Swi4 $\geq$ 3.06	1.92E-5
Met4 $\geq$ 3.1	2.75E-5
Fkh2 $\geq$ 3.24 $\cap$ Mbp1 $\geq$ 3.17	5.44E-5
Gat1 $\geq$ 2.65 $\cap$ Swi4 $\geq$ 3.06	5.49E-5
Cin5 $\geq$ 2.78 $\cap$ Swi4 $\geq$ 3.06	5.49E-5
Mcm1 $\geq$ 5.08 $\cap$ Ndd1 $\geq$ 3.51	5.6E-5
Fkh2 $\geq$ 4.51 $\cap$ Swi4 $\geq$ 3.73	5.77E-5
Mal13 $\geq$ 3.04	9.42E-5
Ino4 $\geq$ 3.06 $\cap$ Swi4 $\geq$ 3.06	9.42E-5
Fkh1 $\geq$ 3.41	9.42E-5
Mth1 $\geq$ 2.3 $\cap$ Swi4 $\geq$ 2.33	9.81E-5
Fkh2 $\geq$ 3.73 $\cap$ Ndd1 $\geq$ 2.53	1.02E-4
Mth1 $\geq$ 3.27	4.86E-4
Fkh1 $\geq$ 4.93 $\cap$ Fkh2 $\geq$ 2.8	4.86E-4
Ash1 $\geq$ 2.8 $\cap$ Cbf1 $\geq$ 2.44	4.86E-4
Ndd1 $\geq$ 3.24 $\cap$ Swi6 $\geq$ 3.65	5.05E-4
Mbp1 $\geq$ 3.17	5.7E-4
Pho4 $\geq$ 9.35	6.49E-4
Ash1 $\geq$ 2.47 $\cap$ Yap3 $\geq$ 2.76	6.49E-4
Swi4 $\geq$ 3.06 $\cap$ Yap3 $\geq$ 2.65	6.62E-4
Grf10(pho2) $\geq$ 5.15 $\cap$ Swi4 $\geq$ 3.06	6.62E-4
Cin5 $\geq$ 2.3 $\cap$ Fkh2 $\geq$ 3.24	6.62E-4
Hap4 $\geq$ 2.42 $\cap$ Ino4 $\geq$ 3.08	6.82E-4
Ecm22 $\geq$ 2.4 $\cap$ Swi4 $\geq$ 3.73	6.89E-4

220min	p
Swi4 $\geq$ 4.66	1.2E-10
Fkh2 $\geq$ 4.07 $\cap$ Swi4 $\geq$ 4.66	3.01E-9
Ash1 $\geq$ 2.42	1.81E-8
Ndd1 $\geq$ 3.24 $\cap$ Swi4 $\geq$ 3.06	3.71E-8
Ash1 $\geq$ 2.42 $\cap$ Swi4 $\geq$ 3.73	4.14E-8
Fkh2 $\geq$ 3.27	8.94E-8
Ndd1 $\geq$ 3.65	2.31E-7
Grf10(pho2) $\geq$ 2.44 $\cap$ Swi4 $\geq$ 4.66	4.43E-7
Met4 $\geq$ 2.96	4.52E-7
Phd1 $\geq$ 2.6	4.99E-7
Ash1 $\geq$ 2.42 $\cap$ Fkh2 $\geq$ 3.27	5.24E-6
Rap1 $\geq$ 2.47 $\cap$ Swi4 $\geq$ 3.73	5.45E-6
Swi4 $\geq$ 2.33 $\cap$ Swi6 $\geq$ 2.35	5.62E-6
Hsf1 $\geq$ 3.38 $\cap$ Swi4 $\geq$ 3.06	2.54E-5
Ste12 $\geq$ 2.32 $\cap$ Swi4 $\geq$ 4.66	2.98E-5
Ash1 $\geq$ 2.8 $\cap$ Swi4 $\geq$ 4.66 $\cap$ Swi6 $\geq$ 2.35	3.04E-5
Hap4 $\geq$ 3.04 $\cap$ Hap5 $\geq$ 3.3	6.25E-5
Ash1 $\geq$ 2.63 $\cap$ Met4 $\geq$ 2.33	1.21E-4
Ash1 $\geq$ 2.42 $\cap$ Mal13 $\geq$ 3.32	1.21E-4
Phd1 $\geq$ 2.94 $\cap$ Rap1 $\geq$ 2.72	5.76E-4
Met4 $\geq$ 2.58 $\cap$ Rgm1 $\geq$ 2.3	5.76E-4
Grf10(pho2) $\geq$ 5.15	5.76E-4
Gcr1 $\geq$ 2.54	5.76E-4
Cin5 $\geq$ 2.47 $\cap$ Mal13 $\geq$ 2.3	5.76E-4
Ash1 $\geq$ 2.63 $\cap$ Phd1 $\geq$ 5.08	5.76E-4
Ash1 $\geq$ 2.3 $\cap$ Ino2 $\geq$ 2.66	5.76E-4
Met4 $\geq$ 2.58 $\cap$ Put3 $\geq$ 3.17	5.93E-4
Ash1 $\geq$ 2.63 $\cap$ Ino4 $\geq$ 3.1	7.32E-4
Cin5 $\geq$ 2.47	8.17E-4

230min	p
Hsf1 $\geq$ 6.81	2.38E-7
Hap4 $\geq$ 2.75 $\cap$ Ino4 $\geq$ 2.47	4.01E-7
Ndd1 $\geq$ 3.24	9.01E-7
Mal13 $\geq$ 2.53 $\cap$ Mth1 $\geq$ 3.02	2.32E-5
Ino2 $\geq$ 2.58 $\cap$ Ino4 $\geq$ 2.47	2.32E-5
Swi4 $\geq$ 3.32	2.44E-5
Hap4 $\geq$ 2.75	2.44E-5
Phd1 $\geq$ 4.51	1.14E-4
Ash1 $\geq$ 3.08	1.14E-4
Hsf1 $\geq$ 2.88 $\cap$ Swi4 $\geq$ 2.47	1.19E-4
Ash1 $\geq$ 2.54 $\cap$ Ndd1 $\geq$ 3.24	1.19E-4
Ino4 $\geq$ 2.47 $\cap$ Mac1 $\geq$ 4.07	2.77E-4
Hsf1 $\geq$ 6.81 $\cap$ Msn4 $\geq$ 2.31	2.83E-4
Mth1 $\geq$ 3.02 $\cap$ Rgm1 $\geq$ 2.3	5.48E-4
Mal13 $\geq$ 2.78 $\cap$ Ndd1 $\geq$ 3.24	5.48E-4
Ino4 $\geq$ 2.47 $\cap$ Swi4 $\geq$ 3.32	5.48E-4
Ino4 $\geq$ 2.47 $\cap$ Phd1 $\geq$ 3.41	5.48E-4
Cin5 $\geq$ 2.73 $\cap$ Hsf1 $\geq$ 2.88	5.48E-4
Ash1 $\geq$ 2.47 $\cap$ Ino4 $\geq$ 2.47	5.48E-4
Ash1 $\geq$ 2.44 $\cap$ Mth1 $\geq$ 3.27	5.48E-4
Cin5 $\geq$ 4.65	5.64E-4
Ino4 $\geq$ 3.86	6.62E-4
Fkh1 $\geq$ 3.41	6.62E-4
Ino4 $\geq$ 2.63 $\cap$ Rtg1 $\geq$ 2.69	7.17E-4
Ino4 $\geq$ 2.47 $\cap$ Swi6 $\geq$ 2.86	7.17E-4
Abf1 $\geq$ 4.02 $\cap$ Ndd1 $\geq$ 3.24	7.17E-4
Ino4 $\geq$ 2.47 $\cap$ Ndd1 $\geq$ 3.24 $\cap$ Phd1 $\geq$ 3.41	7.25E-4
Ndd1 $\geq$ 3.24 $\cap$ Phd1 $\geq$ 6.5	7.32E-4
Ino4 $\geq$ 2.83 $\cap$ Ndd1 $\geq$ 3.24	8.01E-4
Mcm1 $\geq$ 2.62 $\cap$ Ndd1 $\geq$ 3.24	8.17E-4

240min	p
Swi4 $\geq$ 4.61	2.85E-7
Cin5 $\geq$ 2.47 $\cap$ Met4 $\geq$ 2.3	1.36E-6
Cin5 $\geq$ 3.12	1.66E-6
Hsf1 $\geq$ 4.14	3.14E-6
Cin5 $\geq$ 2.47 $\cap$ Ecm22 $\geq$ 2.4	5.67E-6
Cin5 $\geq$ 2.47 $\cap$ Ndd1 $\geq$ 2.53	6.21E-6
Cin5 $\geq$ 2.73 $\cap$ Ino4 $\geq$ 2.58	6.33E-6
Cin5 $\geq$ 2.47 $\cap$ Yap6 $\geq$ 2.69	6.33E-6
Cin5 $\geq$ 2.47 $\cap$ Mth1 $\geq$ 2.72	6.33E-6
Ash1 $\geq$ 2.3 $\cap$ Cin5 $\geq$ 2.47	6.33E-6
Cin5 $\geq$ 2.47 $\cap$ Ino2 $\geq$ 2.31	6.46E-6
Cin5 $\geq$ 2.47 $\cap$ Phd1 $\geq$ 2.56	2.87E-5
Cin5 $\geq$ 2.47 $\cap$ Hsf1 $\geq$ 2.65	2.87E-5
Ash1 $\geq$ 3.38	4.63E-5
Cin5 $\geq$ 2.47 $\cap$ Fkh2 $\geq$ 5.17	6.51E-5
Gat1 $\geq$ 2.65 $\cap$ Swi4 $\geq$ 4.61	6.57E-5
Cin5 $\geq$ 2.47 $\cap$ Fkh2 $\geq$ 2.6 $\cap$ Rap1 $\geq$ 3.0	6.57E-5
Cin5 $\geq$ 2.47 $\cap$ Fkh1 $\geq$ 3.41	6.57E-5
Swi4 $\geq$ 5.12 $\cap$ Swi6 $\geq$ 2.86	1.03E-4
Cin5 $\geq$ 2.47 $\cap$ Gat1 $\geq$ 2.6	1.32E-4
Ino4 $\geq$ 3.24 $\cap$ Swi4 $\geq$ 3.73	1.35E-4
Hsf1 $\geq$ 4.14 $\cap$ Swi4 $\geq$ 3.73	1.35E-4
Hsf1 $\geq$ 4.14 $\cap$ Met4 $\geq$ 3.73	1.35E-4
Gat1 $\geq$ 2.6 $\cap$ Ino4 $\geq$ 3.06	1.35E-4
Ndd1 $\geq$ 2.53 $\cap$ Swi4 $\geq$ 3.73	1.36E-4
Ndd1 $\geq$ 3.58	1.88E-4
Stb1 $\geq$ 2.73	1.9E-4
Hap4 $\geq$ 3.47	1.9E-4
Phd1 $\geq$ 4.51	1.94E-4
Ace2 $\geq$ 2.36 $\cap$ Cin5 $\geq$ 2.47	3.07E-4
Ash1 $\geq$ 3.0 $\cap$ Swi4 $\geq$ 2.48	6.11E-4
Ash1 $\geq$ 3.04 $\cap$ Ecm22 $\geq$ 2.4	6.11E-4
Nrg1 $\geq$ 4.34	6.17E-4
Mss11 $\geq$ 2.45 $\cap$ Sig1 $\geq$ 3.77	6.17E-4
Gat1 $\geq$ 2.65 $\cap$ Ndd1 $\geq$ 2.53	6.17E-4
Cin5 $\geq$ 2.73 $\cap$ Rgm1 $\geq$ 2.3	6.17E-4
Fkh2 $\geq$ 5.17	7.77E-4
Cbf1 $\geq$ 3.91	7.77E-4

250min	p
Hsf1 $\geq$ 3.41	8.92E-7
Hap4 $\geq$ 2.72 $\cap$ Ino4 $\geq$ 2.58	4.69E-6
Hsf1 $\geq$ 3.41 $\cap$ Msn4 $\geq$ 3.41	4.74E-6
Hsf1 $\geq$ 2.39 $\cap$ Swi4 $\geq$ 2.7	2.1E-5
Ace2 $\geq$ 19.26	5.6E-5
Cad1 $\geq$ 3.08 $\cap$ Pho4 $\geq$ 3.02	5.77E-5
Ino4 $\geq$ 2.98	8.03E-5
Ace2 $\geq$ 4.42 $\cap$ Fkh2 $\geq$ 2.94	8.96E-5
Hsf1 $\geq$ 2.39 $\cap$ Mal13 $\geq$ 2.32	1.15E-4
Yap6 $\geq$ 3.3	1.39E-4
Fkh1 $\geq$ 3.41	1.46E-4
Ndd1 $\geq$ 2.63	1.51E-4
Mal13 $\geq$ 2.81	3.9E-4
Pho4 $\geq$ 3.02	5.31E-4
Hir2 $\geq$ 2.72	5.31E-4
Fkh1 $\geq$ 3.41 $\cap$ Mbp1 $\geq$ 2.3	5.48E-4
Cin5 $\geq$ 3.22 $\cap$ Yap6 $\geq$ 3.3	5.48E-4
Swi4 $\geq$ 3.06	5.59E-4

270min	p
Fkh2 $\geq$ 2.49	4.09E-7
Fkh1 $\geq$ 3.51 $\cap$ Fkh2 $\geq$ 2.49	1.09E-6
Fkh1 $\geq$ 3.51 $\cap$ Mbp1 $\geq$ 2.3	2.79E-6
Hap4 $\geq$ 2.72 $\cap$ Ino4 $\geq$ 2.58	5.34E-6
Ino4 $\geq$ 2.98	8.05E-6
Fkh1 $\geq$ 3.51	8.72E-6
Mal13 $\geq$ 3.22	2.49E-5
Hsf1 $\geq$ 2.65 $\cap$ Swi4 $\geq$ 3.06	2.49E-5
Yap6 $\geq$ 3.0	2.59E-5
Hsf1 $\geq$ 2.65	2.81E-5
Swi4 $\geq$ 2.7	3.14E-5
Cin5 $\geq$ 3.12	3.75E-5
Ace2 $\geq$ 3.96 $\cap$ Fkh2 $\geq$ 2.94	6.07E-5
Mal13 $\geq$ 2.81 $\cap$ Swi4 $\geq$ 3.12	6.38E-5
Gat1 $\geq$ 2.73 $\cap$ Ino4 $\geq$ 2.58	6.38E-5
Ace2 $\geq$ 12.68	6.51E-5
Skn7 $\geq$ 2.8	1.52E-4
Sig1 $\geq$ 2.44	3.32E-4
Ino4 $\geq$ 2.83 $\cap$ Mal13 $\geq$ 2.43	5.53E-4
Cin5 $\geq$ 3.22 $\cap$ Yap6 $\geq$ 3.0	5.53E-4
Swi6 $\geq$ 4.27	5.7E-4
Mth1 $\geq$ 2.42 $\cap$ Rtg1 $\geq$ 2.69	5.7E-4
Mbp1 $\geq$ 4.61	5.7E-4
Gat1 $\geq$ 2.81 $\cap$ Mal13 $\geq$ 2.43	5.7E-4
Gat1 $\geq$ 2.56 $\cap$ Rtg1 $\geq$ 2.69	5.7E-4
Fkh2 $\geq$ 2.49 $\cap$ Mbp1 $\geq$ 4.61	5.7E-4
Cin5 $\geq$ 2.56 $\cap$ Met4 $\geq$ 2.3	5.7E-4
Sfl1 $\geq$ 3.22	5.87E-4
Ino4 $\geq$ 2.58 $\cap$ Skn7 $\geq$ 2.62	5.87E-4
Ash1 $\geq$ 2.54 $\cap$ Mal13 $\geq$ 3.04	5.87E-4
Ndd1 $\geq$ 2.53	6.11E-4
Hap4 $\geq$ 2.72	7.17E-4

290min	p
Hsf1 $\geq$ 2.39 $\cap$ Mal13 $\geq$ 2.32	9.33E-9
Hsf1 $\geq$ 2.39	2.29E-7
Gat1 $\geq$ 2.56 $\cap$ Ino4 $\geq$ 2.48	1.27E-6
Mal13 $\geq$ 3.04	2.6E-6
Mal13 $\geq$ 2.32 $\cap$ Swi4 $\geq$ 5.12	3.08E-6
Ace2 $\geq$ 3.96	5.67E-6
Hsf1 $\geq$ 2.39 $\cap$ Swi4 $\geq$ 3.06	5.84E-6
Hap4 $\geq$ 2.72 $\cap$ Ino4 $\geq$ 2.48	5.9E-6
Gat1 $\geq$ 2.66 $\cap$ Mal13 $\geq$ 2.32	6.27E-6
Swi4 $\geq$ 4.61	1.03E-5
Ino4 $\geq$ 3.41	1.09E-5
Ace2 $\geq$ 3.96 $\cap$ Fkh1 $\geq$ 3.41	3.2E-5
Swi6 $\geq$ 3.02	4.59E-5
Pho4 $\geq$ 9.35	6.51E-5
Mal13 $\geq$ 2.32 $\cap$ Pho4 $\geq$ 4.99	6.64E-5
Ino4 $\geq$ 2.48 $\cap$ Yap3 $\geq$ 2.55	6.71E-5
Hsf1 $\geq$ 3.41 $\cap$ Mal13 $\geq$ 2.32 $\cap$ Swi4 $\geq$ 5.12	6.71E-5
Ino4 $\geq$ 2.66 $\cap$ Mal13 $\geq$ 2.32	1.27E-4
Mal13 $\geq$ 2.32 $\cap$ Ndd1 $\geq$ 2.76	1.3E-4
Ino4 $\geq$ 2.48 $\cap$ Skn7 $\geq$ 2.62	1.3E-4
Hsf1 $\geq$ 2.39 $\cap$ Skn7 $\geq$ 3.86	1.3E-4
Mal13 $\geq$ 2.32 $\cap$ Skn7 $\geq$ 3.32	1.34E-4
Ace2 $\geq$ 3.96 $\cap$ Swi6 $\geq$ 2.56	1.34E-4
Ecm22 $\geq$ 2.3 $\cap$ Ino4 $\geq$ 2.48	1.36E-4
Gat1 $\geq$ 2.56 $\cap$ Ino2 $\geq$ 2.31 $\cap$ Ino4 $\geq$ 2.54	1.39E-4
Skn7 $\geq$ 3.19	1.45E-4
Yap6 $\geq$ 3.0	1.92E-4
Gat1 $\geq$ 2.73 $\cap$ Ino4 $\geq$ 2.48 $\cap$ Mal13 $\geq$ 2.43	3.01E-4
Ace2 $\geq$ 4.42 $\cap$ Fkh2 $\geq$ 2.94	3.13E-4
Gcn4 $\geq$ 3.08	5.42E-4
Mal13 $\geq$ 2.32 $\cap$ Swi6 $\geq$ 4.27	5.87E-4
Gat1 $\geq$ 2.56 $\cap$ Ino4 $\geq$ 2.98 $\cap$ Mth1 $\geq$ 2.35	5.99E-4
Fkh2 $\geq$ 2.6 $\cap$ Mal13 $\geq$ 2.32	5.99E-4
Ash1 $\geq$ 2.63 $\cap$ Ino4 $\geq$ 2.48	5.99E-4
Ino2 $\geq$ 2.31 $\cap$ Ino4 $\geq$ 2.48	6.11E-4
Cbf1 $\geq$ 5.65	6.11E-4
Mal13 $\geq$ 2.32 $\cap$ Rme1 $\geq$ 2.3	6.24E-4
Grf10(pho2) $\geq$ 2.44 $\cap$ Mal13 $\geq$ 2.32	6.24E-4
Ino2 $\geq$ 3.15	6.82E-4
